# Virtual sensing to enable real-time monitoring of inaccessible locations & unmeasurable parameters

**DOI:** 10.1038/s41467-026-77463-7

**Published:** 2026-09-19

**Authors:** Kazuma Kobayashi, Farid Ahmed, Jaewan Park, Subhankar Sarkar, Seid Koric, Souvik Chakraborty, Syed Bahauddin Alam

**Affiliations:** 1 Nuclear, Plasma, & Radiological Engineering Department, The Grainger College of Engineering, University of Illinois Urbana-Champaign, Urbana, IL USA; 2 Mechanical Science and Engineering Department, The Grainger College of Engineering, University of ILlinois Urbana-Champaign, Urbana, IL USA; 3 National Center for Supercomputing Applications, University of Illinois Urbana-Champaign, Urbana, IL USA; 4 Department of Applied Mechanics, Indian Institute of Technology Delhi, Delhi, India; 5 Yardi School of Artificial Intelligence, Indian Institute of Technology Delhi, Delhi, India

**Keywords:** Nuclear fusion and fission, Energy infrastructure

## Abstract

Real-time monitoring of safety-critical interior states remains an open problem across energy, environmental, and industrial systems where direct instrumentation is infeasible. Existing approaches based on governing equations, discrete state vectors, or fixed sensor locations fail to provide mesh-independent, field-level reconstructions at arbitrary interior coordinates under real-time constraints. Here we introduce neural operator-based virtual sensing as a general framework for recovering inaccessible interior fields from sparse, heterogeneous measurements and operating conditions, instantiated with MIMONet, a multi-input, multi-output neural operator that fuses heterogeneous inputs and decodes coupled physical fields through a shared latent representation. Across three engineering-grade evaluations of escalating complexity, from confined recirculating flows to pressurized water reactor subchannels and compact power-system heat exchangers, MIMONet achieves ≤5% relative reconstruction error, while generating full-field predictions with sub-millisecond inference on an NVIDIA H200, at least five orders of magnitude faster than traditional high-fidelity CFD simulations, with calibrated uncertainty and noise resilience demonstrated on the heat-exchanger case. We validate the framework on three independent real-world datasets spanning electrochemical energy, atmospheric science, and physical oceanography: current-density mapping in a hydrogen fuel cell, wind-speed profiling on instrumented meteorological towers, and North Atlantic ocean-state from a global ocean reanalysis. MIMONet improves over classical virtual-sensing baselines by 72–84%: 84% on fuel-cell internal-field recovery from 10% sensor coverage and 83% on hub-height wind-speed recovery from lower-level tower sensors. Most notably, it recovers sea-surface height, a field with zero direct sensors, from its learned coupling to observed temperature and salinity at 4.1 × lower error than the best classical predictor, a cross-modal field recovery beyond the reach of classical interpolation. These results establish operator-based virtual sensing as a practical route to real-time field observability in systems where the states that matter most cannot be directly measured.

## Abstract (web summary)

The authors introduce an operator-learning framework for real-time virtual sensing that reconstructs inaccessible interior physical fields from sparse, heterogeneous measurements and operating conditions.

## Introduction

Across energy, environmental, and industrial systems, safety-critical physical states are often located where sensors cannot reach: behind radiation shielding, within electrochemical layers, across atmospheric columns, or beneath ocean surfaces. Recovering these interior fields from sparse accessible measurements is therefore central to reactor monitoring, fuel-cell diagnostics, wind-energy assessment, and geophysical observation. The challenge is most acute in next-generation nuclear reactors, whose compact, increasingly autonomous operation demands real-time estimates of internal pressure, temperature, and flow precisely where radiation, thermal limits, and geometric confinement preclude durable instrumentation^1–4^. The same inaccessibility, in a different physical form, recurs across the electrochemical, atmospheric, and oceanographic systems examined here. Although high-fidelity simulation provides indispensable reference fields for design and safety analysis, resolving the coupled governing equations can require hundreds of seconds per solution and is therefore unsuitable for continuous monitoring. This motivates virtual sensing: reconstructing unmeasurable interior states from limited boundary observations. Rather than producing isolated point estimates, an effective virtual sensor must recover coupled fields and derived quantities, such as wall shear and pressure gradients, at arbitrary inaccessible locations while providing millisecond-scale inference and calibrated uncertainty^5–7^. Such capability can restore the physical observability required by digital twins^8,9^.

Virtual sensing encompasses model-based and data-driven approaches^5,8,10^. Model-based methods include Luenberger and unknown-input observers^11^, Kalman-filter families^5,12,13^, moving-horizon estimation^14,15^, Gaussian-process latent-force models^16^, and spatial estimators such as kriging^17^. These methods are effective when the governing dynamics or covariance structure is known. Importantly, classical PDE observers can reconstruct spatially distributed states from boundary measurements; their limitation is therefore not an inability to recover fields in principle. Rather, they recursively propagate a state under an explicit governing model and observation operator, typically using a prescribed grid or spatial basis whose coefficients are estimated. Consequently, evaluation at new spatial coordinates depends on that representation, and the governing model remains part of online inference. Data-driven virtual sensors instead learn input–output relationships directly from measurements, using models ranging from support vector machines and recurrent networks to graph architectures that capture sensor interactions^5,8,10,18–21^. Their established implementations typically return scalar or low-dimensional outputs at a predetermined set of locations fixed during training. The problem considered here occupies a different deployment regime: learning a map from sparse, heterogeneous measurements on a sensing region to a spatially continuous, coupled physical field over a potentially disjoint reconstruction region. Once trained, the model must evaluate this field at arbitrary query coordinates through a single forward pass, without recursively evolving an explicit PDE model or retraining for each operating condition. The distinction is therefore not between methods that can and cannot estimate hidden states, but between online model-based state propagation and amortized, coordinate-conditioned field reconstruction. Achieving the latter requires learning mappings between input and output functions. The question addressed here is therefore whether operator-based virtual sensing can reconstruct spatially continuous, coupled interior fields at arbitrary inaccessible coordinates from sparse, heterogeneous measurements while providing real-time inference and calibrated uncertainty.

Scientific machine learning offers two distinct approaches to field reconstruction. Physics-informed neural networks (PINNs) represent the solution of an individual PDE instance by embedding the governing equations, boundary conditions, and available observations in the training objective^22–25^. Their mesh-free formulation has enabled forward and inverse analysis across complex physical systems, but standard PINNs require instance-specific optimization when boundary conditions or system parameters change. Neural operators address a fundamentally different problem: rather than approximating a single solution function, they learn an operator ${{\mathcal{G}}}$ between input and output function spaces across a family of admissible conditions^26^. This amortizes the training cost and enables rapid inference for previously unseen operating states. Physics-informed neural operators (PINOs) combine these paradigms by imposing governing-equation residuals during operator training, thereby reducing dependence on labelled solution data while retaining operator-level generalization^27–29^. Coordinate-conditioned operators such as DeepONet are particularly suited to virtual sensing because they evaluate reconstructed fields at arbitrary query points without retraining or regridding. Other operator families provide complementary representations of the underlying functional map. FNO captures global interactions through Fourier-domain kernel integration^30^; WNO uses spatially and spectrally localized wavelet representations to resolve multiscale solution features^31^; and GeoFNO extends spectral learning to irregular geometries through a learned correspondence with a structured computational domain^32,33^. Graph-based operators directly accommodate irregular domains and unstructured discretizations: GNO performs kernel integration over domain graphs^34^, while Sp^2^GNO combines spatial message passing with spectral graph representations to capture both localized interactions and long-range dependencies^35^. Attention-based and nonlinear-decoder architectures, including OFormer, NOMAD, and CoDA-NO, further improve the representation of complex operator maps^36–38^. These developments establish a broad foundation for learning PDE solution operators, but their published formulations were designed primarily for PDE surrogacy and do not jointly address the combination required for field-level virtual sensing: sparse and heterogeneous scalar- and function-valued observations, geometrically separated sensing and reconstruction domains, and simultaneous recovery of multiple coupled physical fields. This regime requires an operator that fuses heterogeneous observations into a shared physical representation and decodes interdependent fields at arbitrary inaccessible coordinates.

To address this gap, we introduce MIMONet, a multi-input, multi-output branch–trunk neural operator designed for field-level virtual sensing. Multiple-input operator learning was established by MIONet^39^, which extends DeepONet to several input function spaces through a tensor-product coupling of the branch representations. MIMONet adopts this multi-input construction and extends it to the multi-output, coordinate-conditioned setting required for virtual sensing: separate branch networks encode heterogeneous observations, including scalar operating conditions, spatially distributed functions, and time-dependent sensor histories, and a shared trunk network maps arbitrary query coordinates to channel-specific basis representations that decode multiple physical fields from the same fused latent state. Sharing the latent representation across outputs enables the model to capture dependencies among coupled quantities such as pressure, velocity, temperature, and turbulent kinetic energy, rather than treating each field as an independent regression target. The resulting operator maps sparse measurements to spatially continuous, multi-field reconstructions at arbitrary coordinates, including locations geometrically separated from the sensing region, without requiring regridding or online solution of the governing equations. Combined with stochastic inference and conformal calibration, the framework also provides uncertainty intervals with prescribed empirical coverage. MIMONet thus unifies heterogeneous sensor fusion, cross-domain field reconstruction, coupled-output decoding, and uncertainty-aware real-time inference within a single operator architecture.

The problem class can be stated precisely. This work learns an operator ${{\mathcal{G}}}:{{\mathcal{U}}}\to {L}^{2}({{\mathcal{Y}}};{{\mathbb{R}}}^{m})$ that maps sparse observations acquired on a sensing region ${{\mathcal{X}}}$ to continuous, coupled fields over a potentially disjoint reconstruction region ${{\mathcal{Y}}}$. Unlike classical state observers, it does not recursively propagate a state using an explicit governing model during inference; unlike regression-based virtual sensors, its output coordinates are not fixed during training. Throughout this Article, real-time denotes amortized forward-pass evaluation within a monitoring loop, rather than temporal forecasting or recursive state estimation.

We evaluate MIMONet through two complementary validation stages. First, three engineering-grade thermal–fluid configurations of increasing complexity, lid-driven cavity flow, a pressurized-water-reactor subchannel, and a compact power-system heat exchanger, test whether operator-based virtual sensing remains accurate as geometric confinement, multiphysics coupling, and sensor inaccessibility increase. Across these cases, MIMONet is compared with GeoFNO, NOMAD, and the learned-kriging model KCN^40^, and achieves relative reconstruction errors of at most 5% and sub-millisecond inference throughout, with calibrated uncertainty and robustness under substantial sensor corruption characterized in detail on the heat-exchanger case. Second, we assess practical performance on three independent real-world datasets: internal current-density reconstruction in a proton-exchange-membrane fuel cell^41^, hub-height wind-speed reconstruction from lower-level measurements in the IEA-Wind tall-tower dataset^42^, and North Atlantic ocean-state reconstruction from sparse, disjoint observations in GLORYS12^43^. These problems progress from sparse internal-field recovery to extrapolation beyond the instrumented range and, finally, to cross-modal field recovery: reconstructing sea-surface height, a field that no sensor observes, from its learned coupling to the observed temperature and salinity fields. MIMONet reduces error by 84% for the fuel-cell field and 83% for hub-height wind speed relative to the best practical classical baselines, while reducing sea-surface-height error by a factor of 4.1. Together, the reference-field and real-world studies establish operator-based virtual sensing as a general framework for real-time field observability whenever the physical states of interest are inaccessible, uninstrumented, or not directly measurable, with the nuclear thermal–fluid case as its most safety-critical instance.

## Results

The evaluation proceeds in two stages, distinguished by how the reference fields are obtained. In the first, the interior field is known everywhere because it is computed, so accuracy can be quantified across the whole domain rather than only where instrumentation reaches. This permits a controlled test across a lid-driven cavity, a pressurized-water-reactor subchannel, and a compact power-system heat exchanger (Problems 1–3), together with calibrated uncertainty, comparative accuracy, robustness under degraded sensing, local parameter extrapolation, and inference efficiency. The second stage is a real-world validation on measured and observation-constrained data, in which the operator is compared with the methods practitioners deploy on such data (Problems 4–6). The first establishes what the formulation recovers when the target is fully characterized; the second, whether that capability survives the sparsity and incomplete coverage of real measurement.

### Operator formulation and evaluation on reference thermal-fluid fields

#### Operator-based virtual-sensing framework

**Fig. 1:**
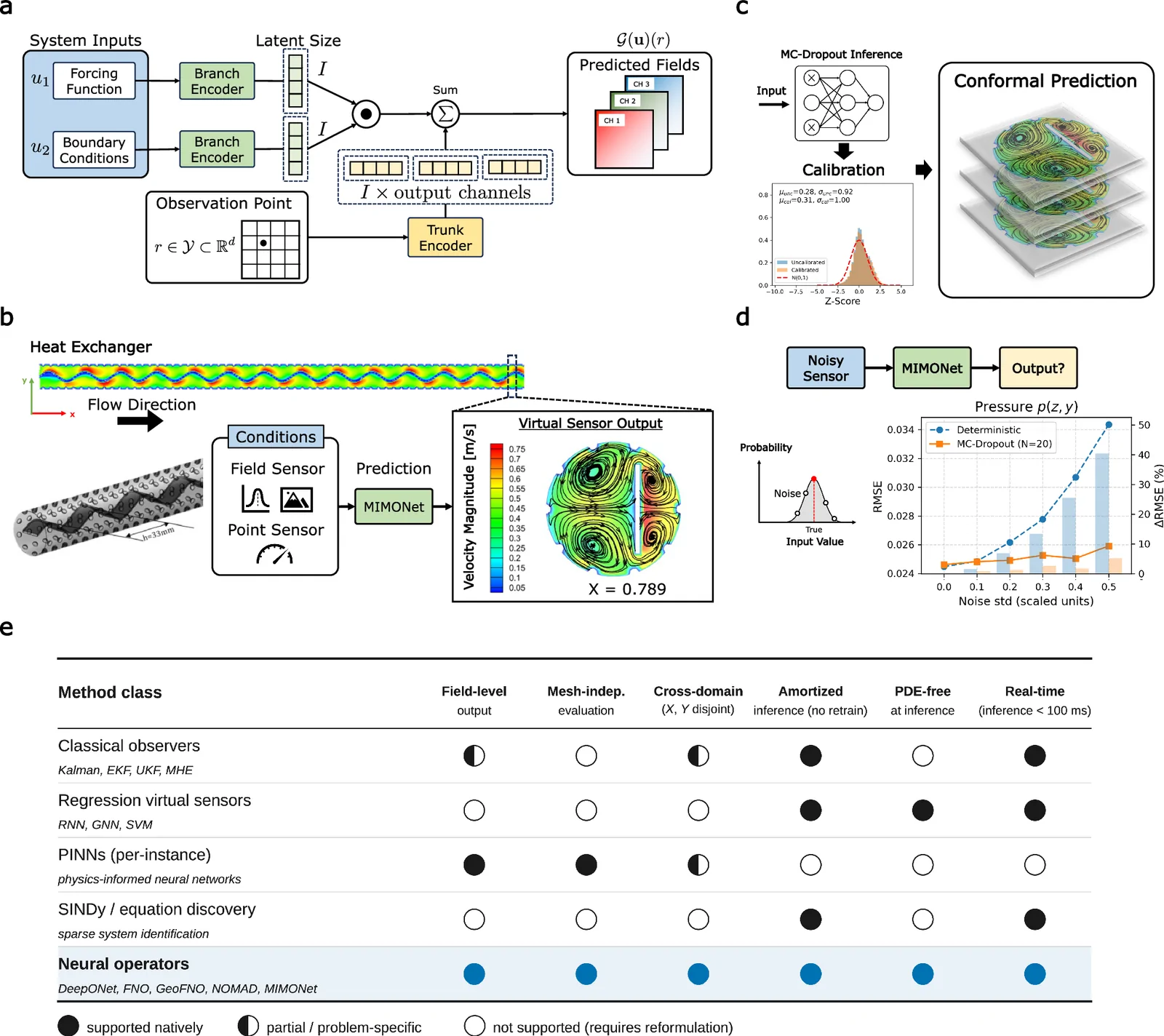
MIMONet for full-field virtual sensing from sparse, heterogeneous measurements. **a** Modality-specific branch encoders map system inputs ***u*** = (*u*_1_, …, *u*_*n*_) into a shared latent representation through multiplicative fusion; a trunk network decodes it at arbitrary query coordinates ${{\bf{r}}}\in {{\mathcal{Y}}}$. **b** The trained operator reconstructs coupled pressure, temperature and velocity fields in inaccessible regions from accessible sensors and operating conditions. **c** MC-dropout predictive intervals are rescaled on a held-out conformal-calibration set to a nominal coverage of 95%. **d** Robustness is assessed by injecting Gaussian noise into the scaled sensor inputs. **e** Capability comparison across virtual-sensing method classes; filled, half-filled and open circles denote native, partial and unsupported capability.

We formulate virtual sensing as an amortized operator that maps accessible observations to spatially continuous interior fields. Unlike recursive state estimation, the governing model is not solved during deployment; throughout this work, real-time therefore refers to forward-pass latency within a monitoring loop rather than temporal forecasting or recursive filtering. Where an input carries a temporal history, the operator consumes the complete signal rather than the causal segment available up to the current instant, so its estimation setting is analogous to fixed-interval smoothing rather than to causal filtering. The real-time comparison is accordingly made against the seconds-to-minutes cost of the high-fidelity solver that would otherwise supply the field, not against the contemporaneous update of a recursive state estimator. Let ${{\boldsymbol{u}}}=({u}_{1},\ldots,{u}_{n})\in {{\mathcal{U}}}$ denote the available heterogeneous observations, where each component ${u}_{i}\in {{{\mathcal{F}}}}_{i}$ may be a scalar operating condition, a spatially distributed function, or a time-dependent sensor history. The target is an *m*-channel physical field ${{\bf{s}}}({{\bf{r}}})\in {{\mathbb{R}}}^{m}$ evaluated at query coordinates **r** on a reconstruction set ${{\mathcal{Y}}}\subset D\subset {{\mathbb{R}}}^{d}$. Field-level virtual sensing therefore amounts to learning the forward operator

1 $${{\mathcal{G}}}:{{\mathcal{U}}}\to {{\mathcal{S}}},\qquad {{\mathcal{G}}}({{\boldsymbol{u}}})({{\bf{r}}})={{\bf{s}}}({{\bf{r}}}),\qquad {{\bf{r}}}\in {{\mathcal{Y}}},$$

where ${{\mathcal{S}}}={L}^{2}({{\mathcal{Y}}};{{\mathbb{R}}}^{m})$. When observations are acquired over a sensing region ${{\mathcal{X}}}$ that is disjoint from ${{\mathcal{Y}}}$, the formulation describes cross-domain inference, ${{\mathcal{X}}}\cap {{\mathcal{Y}}}={{\emptyset}}$. Inaccessible locations remain within the spatial domain represented during training; they are inaccessible to physical instrumentation at deployment rather than necessarily outside the training distribution. Because **r** is an explicit input to the decoder, the trained operator can be queried at coordinate sets different from those used during training, although accuracy at previously unsampled coordinates remains an empirical generalization property. MIMONet instantiates the operator ${{\mathcal{G}}}$ through modality-specific branch encoders and a coordinate-conditioned trunk network (Fig. 1a). Each branch encoder ${\phi }_{i}:{{{\mathcal{F}}}}_{i}\to {{\mathbb{R}}}^{I}$ maps an input component *u*_*i*_ to a latent vector of width *I*. The encoded inputs are fused element-wise into a shared latent representation,

2 $${\psi }_{j}={\prod }_{i=1}^{n}{[{\phi }_{i}({u}_{i})]}_{j},\qquad j=1,\ldots,I.$$

A trunk network maps each query coordinate ${{\bf{r}}}\in {{\mathbb{R}}}^{d}$ to per-channel decoding bases, $\tau :{{\mathbb{R}}}^{d}\to {{\mathbb{R}}}^{I\times m}$, with components *τ*_*j*,*o*_(**r**). The prediction for output channel *o* is

3 $${\hat{s}}_{o}({{\bf{r}}},\{{u}_{i}\})={\sum }_{j=1}^{I}{\tau }_{j,o}({{\bf{r}}}){\prod }_{i=1}^{n}{\phi }_{i,j}({u}_{i})+{\beta }_{o},$$

where ${\phi }_{i,j}({u}_{i})={[{\phi }_{i}({u}_{i})]}_{j}$ and *β*_*o*_ is a channel-specific bias. The product over the encoded inputs introduces explicit cross-modal interactions before coordinate-conditioned decoding, while all output channels share the same fused representation ***ψ***. Channel-specific trunk bases retain the flexibility required to represent physical quantities with different spatial structures and scales.

This construction extends the branch–trunk formulation in three respects: separate branch networks accommodate heterogeneous scalar- and function-valued observations; multiplicative fusion introduces interactions among the encoded modalities; and shared-latent decoding reconstructs multiple coupled fields without learning an independent regressor for every field and spatial location. The shared representation enables the model to capture dependencies among quantities such as pressure, velocity, temperature, and turbulent kinetic energy. It does not, however, explicitly enforce incompressibility, momentum balance, energy conservation, or other governing constraints; these properties remain empirical rather than architectural guarantees. For a batch of *B* inputs evaluated at *P* coordinates, the branch–trunk contraction scales as ${{\mathcal{O}}}(BPIm)$, supporting dense field reconstruction within real-time latency budgets. Figure 1e positions this formulation relative to established virtual-sensing and state-estimation classes. Classical observers support real-time recursive estimation when an explicit governing model is available, while regression-based virtual sensors provide rapid model-free estimates at locations generally fixed during training. Standard PINNs recover continuous fields but require instance-specific optimization for new boundary conditions or parameter configurations. Equation-discovery methods identify an explicit dynamical model rather than directly reconstructing inaccessible fields. At the method-class level, neural operators can jointly support continuous field output, coordinate-independent evaluation, cross-domain reconstruction, and amortized inference. This capability comparison motivates quantitative evaluation against methods operating in a compatible field-reconstruction regime: GeoFNO, NOMAD, and the learned-kriging model KCN.

#### Boundary-only reconstruction of confined recirculating flow

**Fig. 2:**
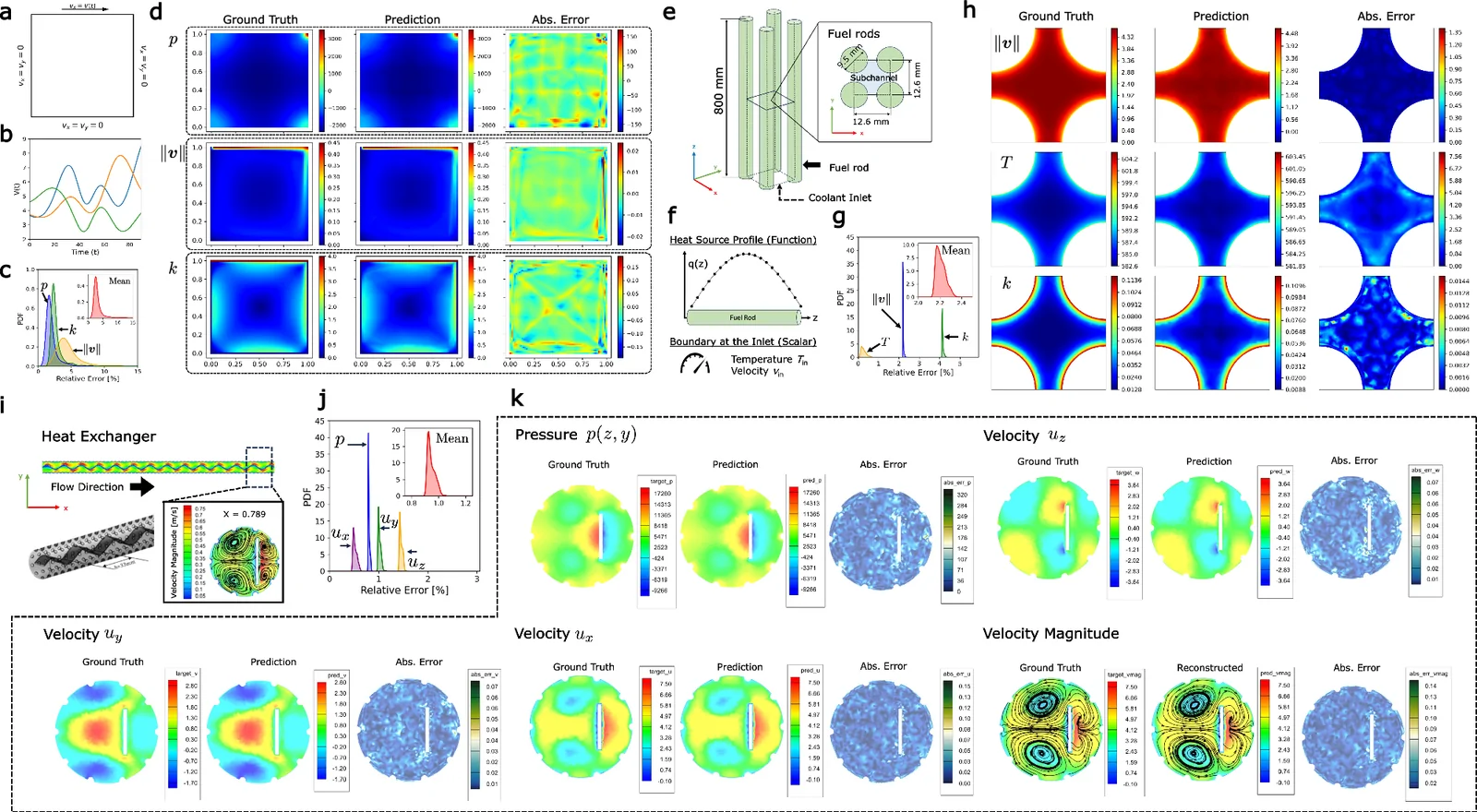
Full-field virtual sensing across three case studies. **a**–**d** Lid-driven cavity. **e**–**h** PWR subchannel. **i**–**k** Periodic compact heat exchanger. **a** Cavity configuration and boundary conditions, with the top wall actuated by the time-dependent lid velocity *V*(*t*). **b** Representative lid-velocity histories sampled from the dataset. **e** Subchannel geometry, fuel rods and transverse reconstruction plane, driven by an axial heat-source profile and scalar inlet conditions. **f** Representative axial heat-source functions *q*(*z*) and the accessible inlet measurements. **i** Heat-exchanger geometry, target plane at *x* = 0.789 and representative internal flow structure. **c**, **g**, **j** Test-set distributions of the relative *ℓ*_2_ error for each reconstructed field; insets show the distribution of the sample-wise mean across fields. **d**, **h**, **k** Ground truth, MIMONet prediction and pointwise absolute error for a representative test sample, taken at the median sample-wise mean relative *ℓ*_2_ error (**d**, **h**) and at the lowest mean error (**k**). **k** additionally shows the velocity magnitude with superimposed streamlines.

The lid-driven cavity (LDC), defined on the unit square Ω = [0, 1] × [0, 1], provides a controlled test of field-level virtual sensing under a boundary-only information constraint. Although geometrically simple, the flow develops a primary vortex, secondary corner recirculation, a high-shear layer beneath the moving lid, and spatially localized turbulent-kinetic-energy production (Fig. 2a). These structures make the cavity a useful first test of whether boundary actuation contains sufficient information to reconstruct spatially distributed interior quantities. The CFD data are generated using the incompressible RANS equations with a *k*–*ε* turbulence closure, as described in Supplementary.

A time-dependent velocity is imposed on the top wall:

4 $${v}_{x}(x,1,t)=V(t),\qquad {v}_{y}(x,1,t)=0,\qquad x\in [0,1],$$

while the remaining walls satisfy the no-slip condition, ***v*** = (0, 0). The system is initialized with zero velocity and uniform pressure, ***v***(*x*, *y*, 0) = (0, 0), *p*(*x*, *y*, 0) = *p*_0_. Consequently, the lid-velocity history *V*(*t*) is the only observable input, while the complete interior flow state remains unavailable to direct measurement. The input function *V*(*t*) is randomly generated and sampled at 90 time levels over the simulation interval (Fig. 2b), giving ${{{\mathcal{U}}}}_{{{\rm{ldc}}}}=\left\{V\in {{\mathbb{R}}}^{90}\right\}$. The reconstruction targets are the final-time pressure *p*, velocity magnitude $\parallel {{\boldsymbol{v}}}\parallel=\sqrt{{v}_{x}^{2}+{v}_{y}^{2}}$, and turbulent kinetic energy *k*, with output space ${{{\mathcal{S}}}}_{{{\rm{ldc}}}}={\left({L}^{2}({{\Omega }})\right)}^{3}$. The corresponding boundary-to-field operator is

5 $${{{\mathcal{G}}}}_{{{\rm{ldc}}}}(V)(x,y)=\left[\begin{array}{c}p(x,y)\\ \parallel {{\boldsymbol{v}}}(x,y)\parallel \\ k(x,y)\end{array}\right],\qquad (x,y)\in {{\Omega }}.$$

The complete lid-velocity history is supplied as an input function, and the target is the spatial field at the prescribed final time. The task is therefore boundary-to-field reconstruction rather than causal forecasting or recursive temporal filtering.

The dataset comprises 4937 independently generated boundary-input/field-output pairs on a 4225-node unstructured mesh. It was divided at the trajectory level into 3159 training, 790 validation, and 988 held-out test samples, ensuring that complete lid-velocity histories remain confined to individual splits and preventing temporal leakage. A single-branch MIMONet is used for this case. The 90-dimensional lid-velocity input is encoded by a fully connected branch network with layer widths [90, 512, 512, 512, 256], while the spatial coordinates (*x*, *y*) are encoded by a trunk network with widths [2, 256, 256, 256, 256 × 3]. The branch and trunk share a latent width of *I* = 256, and their contraction produces the three outputs {*p*, ∥***v***∥, *k*}. ReLU activations are used throughout, with no additional output-transform head. The model is trained using an equally weighted mean-squared-error loss across the three output channels and the Adam optimizer. The initial learning rate is 10^−3^ with a weight decay of 10^−7^; the learning rate is reduced by a factor of 0.5 when the validation loss plateaus. Training proceeds for at most 500 epochs with a batch size of 8 and early stopping after 10 consecutive epochs without validation improvement. The branch inputs are min–max normalized to [−1, 1]. Each output channel is independently transformed to the same range using scaling parameters fitted exclusively on the training set, preventing differences in physical units and magnitudes from causing one field to dominate the loss.

MIMONet achieves mean relative *ℓ*_2_ errors of 2.1% for pressure, 5.0% for velocity magnitude, and 2.8% for turbulent kinetic energy, corresponding to a channel-mean error of 3.3% (Fig. 2c; complete per-channel results for all four models in Supplementary Table S11). It attains the lowest error in every output channel. The corresponding channel-mean errors are 25.0% for KCN, 41.9% for NOMAD, and 68.0% for GeoFNO, making the MIMONet error approximately 7.6–20.6 × lower across the three baselines. The comparatively larger error in ∥***v***∥ is consistent with the sensitivity of the thin shear layer and recirculation boundaries to small spatial displacements. The test-set distributions in Fig. 2c are concentrated at low error, and the sample-wise mean is unimodal without a pronounced high-error tail. Performance is therefore not driven by a small subset of favourable lid histories. In the median-error example (Fig. 2d), MIMONet recovers the primary recirculating vortex, secondary corner structures, and the shear layer beneath the moving lid without visible spurious oscillations. Residual errors concentrate near the moving wall, corner recirculation regions, and zones of elevated turbulent kinetic energy, where the fields exhibit their strongest spatial gradients. Errors in *p*, ∥***v***∥, and *k* concentrate around the same dynamically sensitive structures. This behaviour is consistent with the shared-latent decoder representing common flow features across the output fields, although it does not establish exact satisfaction of the governing conservation laws. Overall, the cavity study demonstrates that spatially distributed interior fields can be reconstructed from boundary actuation alone before introducing the geometric confinement and multimodal inputs of the reactor-subchannel and heat-exchanger cases.

#### Coupled-field reconstruction in a PWR subchannel

The pressurized-water-reactor (PWR) subchannel introduces geometric confinement, distributed thermal forcing, and coupled thermal–hydraulic outputs beyond the boundary-only cavity problem. Coolant passes through narrow, instrument-inaccessible regions between the fuel rods, where heating and wall confinement generate steep temperature gradients, secondary flow, and localized turbulence (Fig. 2e). The objective is to reconstruct the transverse temperature, velocity, and turbulent-kinetic-energy fields from the known axial power distribution and accessible inlet conditions, without measurements on the target cross-section. The input comprises the axial heat-source distribution

6 $$q(z)=A\sin \left(\frac{\pi z}{H}\right),\qquad z\in [0,H],$$

together with the scalar inlet temperature *T*_in_ and inlet velocity *v*_in_ (Fig. 2f). The heat-source amplitude *A* controls the magnitude of the distributed thermal forcing, while the domain height is fixed at *H* = 800 mm. The resulting mixed functional-parametric input space is ${{{\mathcal{U}}}}_{{{\rm{sub}}}}=\left\{(q,{T}_{{{\rm{in}}}},{v}_{{{\rm{in}}}}):q\in {L}^{2}([0,H]),\ {T}_{{{\rm{in}}}},{v}_{{{\rm{in}}}}\in {\mathbb{R}}\right\}$. The operating envelope is generated by independently sampling $A \sim {{\mathcal{U}}}(540,660)\ [{{\rm{kW}}}/{{{\rm{m}}}}^{2}]$, ${T}_{{{\rm{in}}}} \sim {{\mathcal{U}}}(512.1,625.9)\ [{{\rm{K}}}]$,and ${v}_{{{\rm{in}}}} \sim {{\mathcal{U}}}(4.05,4.95)\ [{{\rm{m/s}}}]$. The response is evaluated on a transverse plane ${{{\mathcal{Y}}}}_{{{\rm{sub}}}}\subset {{\mathbb{R}}}^{2}$ at a fixed axial position *z* = *z*_0_. The reconstruction targets are the temperature *T*(*x*, *y*), turbulent kinetic energy *k*(*x*, *y*), and velocity magnitude ∥***v***(*x*, *y*)∥, giving ${{{\mathcal{S}}}}_{{{\rm{sub}}}}={\left({L}^{2}({{{\mathcal{Y}}}}_{{{\rm{sub}}}})\right)}^{3}$. The corresponding operator is

7 $${{{\mathcal{G}}}}_{{{\rm{sub}}}}(q,{T}_{{{\rm{in}}}},{v}_{{{\rm{in}}}})(x,y)=\left[\begin{array}{c}T(x,y)\\ k(x,y)\\ \parallel {{\boldsymbol{v}}}(x,y)\parallel \end{array}\right],\qquad (x,y)\in {{{\mathcal{Y}}}}_{{{\rm{sub}}}}.$$

This formulation requires the operator to combine distributed heating with inlet-driven advection to recover three coupled fields on a geometrically inaccessible plane.

The dataset contains 5000 independently generated operating conditions, divided into 3200 training, 800 validation, and 1000 held-out test samples. Each input is paired with the three output fields evaluated on a 1733-node unstructured mesh, giving a discretized output in ${{\mathbb{R}}}^{1733\times 3}$. The three-dimensional ANSYS Fluent configuration, governing thermal–hydraulic equations, mesh, turbulence closure, and convergence requirements, is described in Supplementary Methods. The subchannel model follows the MIMONet configuration used for the LDC case, with problem-specific modifications for its mixed functional-parametric input. The heat-source profile *q*(*z*), discretized at 100 axial locations, is encoded by a branch network with widths [100, 512, 512, 512, 256], while the scalar pair (*T*_in_, *v*_in_) is encoded by a second branch with widths [2, 512, 512, 512, 256]. The two latent representations are fused multiplicatively before coordinate-conditioned decoding. The trunk architecture, [2, 256, 256, 256, 256 × 3], latent width *I* = 256, and ReLU activations are unchanged from the LDC configuration. The three decoded outputs are {*T*, *k*, ∥***v***∥}. Unless otherwise stated, the optimization and output-scaling procedures are also the same as in the LDC case: training uses an equally weighted mean-squared-error loss, the Adam optimizer with an initial learning rate of 10^−3^, a learning-rate reduction factor of 0.5, at most 500 epochs, and independent output scaling to [−1, 1]. For the subchannel case, the weight decay is 10^−6^, the learning rate is reduced after ten consecutive epochs without validation-loss improvement, and the batch size is 4. Unlike the min–max-normalized LDC input, the heat-source and inlet-condition inputs are standardized using means and variances fitted on the subchannel training split.

MIMONet achieves mean relative *ℓ*_2_ errors of 0.27% for temperature, 2.2% for velocity magnitude, and 4.2% for turbulent kinetic energy, corresponding to a channel-mean error of 2.2% (Fig. 2g; complete per-channel results for all four models in Supplementary Table S11). It attains the lowest error for every output channel. The corresponding channel-mean errors are 53.0% for KCN, 67.8% for NOMAD, and 72.2% for GeoFNO, making the MIMONet error ~24–33 × lower across the three baselines. The test-set distributions in Fig. 2g are sharply concentrated at low error. The sample-wise mean distribution is centred near 2.2% with a narrow spread and no pronounced high-error tail, indicating consistent performance across unseen combinations of heat-source amplitude and inlet conditions. The error hierarchy is also physically interpretable: temperature is comparatively smooth and diffusion-regularized, velocity is more sensitive to advective transport and wall confinement, and turbulent kinetic energy contains sharper localized structures and consequently exhibits the largest error. In the median-error example (Fig. 2h), MIMONet recovers the high-speed interior flow, its attenuation near the no-slip fuel-rod surfaces, the wall-to-core temperature variation, and the localized turbulent-kinetic-energy peaks adjacent to heated boundaries. Residual errors remain low through most of the flow interior and increase primarily near wall-adjacent shear layers and steep thermal gradients, where small spatial discrepancies produce larger pointwise errors. No obvious systematic directional bias is visible in the residual maps. The co-location of residuals across *T*, ∥***v***∥, and *k* is consistent with the shared latent representation capturing common thermal-hydraulic structures, although it does not establish exact satisfaction of the governing equations. Overall, the subchannel study shows that heterogeneous, distributed, and scalar inputs can reconstruct coupled fields on an inaccessible cross-section, extending the boundary-only result to a reactor-relevant geometry with multimodal forcing.

#### Pressure–velocity reconstruction in a compact heat exchanger

The third configuration considers a periodic compact heat exchanger, in which geometric confinement and elevated operating conditions preclude direct instrumentation of the internal pressure and velocity fields^44^. Compared with the PWR subchannel, this problem introduces a fully three-dimensional velocity field, stronger pressure–velocity coupling, and recirculation generated by an internal fin. The virtual-sensing task is to reconstruct these coupled fields on an inaccessible interior plane using only the applied wall heat flux and the scalar inlet temperature and velocity.

As in the subchannel case, the axial heat-source profile is prescribed as

8 $$q(z)=A\sin \left(\frac{\pi z}{H}\right),\qquad z\in [0,H],$$

where *A* denotes the heat-flux amplitude. This profile is extruded uniformly along the streamwise coordinate *x* to define the wall forcing,

9 $${q}_{{{\rm{wall}}}}(x,z): \!=q(z),\qquad x\in [0,L].$$

Together with the scalar inlet temperature *T*_in_ and axial velocity magnitude *u*_in_, the wall profile defines the mixed functional-parametric input ${{{\boldsymbol{u}}}}_{{{\rm{hx}}}}=\left({q}_{{{\rm{wall}}}},{T}_{{{\rm{in}}}},{u}_{{{\rm{in}}}}\right)\in {{{\mathcal{U}}}}_{{{\rm{hx}}}}$. The response is evaluated on a vertical plane ${{{\mathcal{Y}}}}_{{{\rm{hx}}}}\subset {{\mathbb{R}}}^{2}$ at the fixed transverse location *x* = *x*_0_, spanning the (*z*, *y*) coordinates. The output comprises the pressure and all three velocity components, ${{{\mathcal{S}}}}_{{{\rm{hx}}}}={\left({L}^{2}({{{\mathcal{Y}}}}_{{{\rm{hx}}}})\right)}^{4}$, and the corresponding operator is

10 $${{{\mathcal{G}}}}_{{{\rm{hx}}}}\left({q}_{{{\rm{wall}}}},{T}_{{{\rm{in}}}},{u}_{{{\rm{in}}}}\right)(z,y)=\left[\begin{array}{c}p(z,y)\\ {u}_{x}(z,y)\\ {u}_{y}(z,y)\\ {u}_{z}(z,y)\end{array}\right],\qquad (z,y)\in {{{\mathcal{Y}}}}_{{{\rm{hx}}}}.$$

The model must therefore propagate a distributed thermal input and scalar inlet conditions into a spatially resolved pressure field and three-component velocity field on a domain disjoint from the accessible inputs.

For each operating condition, the converged CFD solution was sampled on the plane *x* = 0.789, producing a two-dimensional unstructured mesh containing 3977 nodes. The plane intersects developing-flow regions, near-wall shear layers, and the recirculation zones surrounding the internal fin (Fig. 2i). The dataset comprises 1546 simulations generated by varying the wall-flux amplitude, inlet temperature, and inlet axial velocity. Of these, 1236 samples were used for training and 310 were retained as a held-out test set; a validation subset was drawn from the training data during optimization. Each input is paired with an output array in ${{\mathbb{R}}}^{3977\times 4}$ containing {*p*, *u*_*x*_, *u*_*y*_, *u*_*z*_}. The governing equations, turbulence treatment, boundary conditions, and CFD solution procedure are provided in Supplementary. The MIMONet architecture mirrors that used for the PWR subchannel, with the output trunk expanded from three to four channels. The 100-point wall-flux profile is encoded by a functional branch with widths [100, 512, 512, 512, 256], while the inlet pair (*T*_in_, *u*_in_) is encoded by a scalar branch [2, 512, 512, 512, 256]. Coordinates (*z*, *y*) on the target plane are processed by a trunk network [2, 256, 256, 256, 256 × 4]. The two 256-dimensional branch representations are fused multiplicatively and contracted with the trunk representation to predict {*p*, *u*_*x*_, *u*_*y*_, *u*_*z*_} simultaneously. The optimization and normalization procedures were otherwise the same as for the PWR subchannel. The model was trained using an equally weighted mean-squared error over the four output channels and the Adam optimizer, with an initial learning rate of 10^−3^ and a weight decay of 10^−6^. The learning rate was reduced by a factor of 0.5 after ten epochs without improvement in the validation loss, and training continued for at most 500 epochs with a batch size of 4. The functional and scalar inputs were standardized using statistics computed from the training set, and each output channel was independently mapped to [−1, 1] using training-set extrema.

The test-error distributions in Fig. 2j are sharply concentrated at low values for all four outputs. The mean relative *ℓ*_2_ errors are 0.82% for pressure, 1.45% for *u*_*z*_, 1.02% for *u*_*y*_, and 0.52% for *u*_*x*_, giving a channel-averaged error of 0.95%. The corresponding distribution of sample-wise mean errors is narrow and centred near 1%, indicating consistent performance across the sampled combinations of wall heating and inlet conditions. The representative fields in Fig. 2k show that MIMONet recovers the pressure variation and the distinct spatial structures of all three velocity components. Residuals remain small over most of the target plane and increase primarily near the internal fin, geometric junctions, and regions containing steep shear gradients. Their localization in these regions is consistent with the greater spatial complexity and sensitivity of the associated flow structures. The reconstructed velocity magnitude and streamlines in Fig. 2k preserve the two dominant counter-rotating recirculation cells adjacent to the internal fin, together with the secondary structures near the upper and lower junctions. The predicted vortex cores, separatrices, and stagnation regions closely follow those in the reference solution, while the largest residuals remain localized near the fin and regions of elevated shear. The agreement between the component fields and the reconstructed streamline topology indicates that the low field-wise errors also preserve the principal internal flow organization, which is relevant for identifying maldistribution and flow-induced loading in locations that cannot be instrumented directly.

#### Calibrated uncertainty for inaccessible-field reconstruction

Pointwise reconstruction accuracy does not, by itself, indicate whether an individual field prediction should be trusted. This distinction is particularly important in virtual sensing because the inferred quantities cannot be checked against interior measurements during operation. We therefore augment MIMONet with spatially resolved predictive uncertainty and calibrate the resulting intervals on held-out data. For the heat-exchanger case, dropout layers with probability *p* = 0.10 were added to every hidden layer of the two-branch networks and the trunk network. All other architectural and optimization settings were retained from the deterministic model. The dataset was repartitioned into 1082 training samples, 154 calibration samples, and the unchanged 310-sample test set; a validation subset was drawn from the training partition. During inference, dropout remains active and *N* = 20 stochastic forward evaluations are performed for each input. For output channel *c*, the resulting operator realizations are

11 $${s}_{{c}}^{(e)}({{\bf{r}}})={{{\mathcal{G}}}}_{{c}}^{(e)}({{\boldsymbol{u}}})({{\bf{r}}}),\qquad e=1,\ldots,N,$$

from which the predictive mean and variance are estimated as

12 $${\bar{s}}_{{c}}({{\bf{r}}})=\frac{1}{N}{\sum }_{e=1}^{N}{s}_{{c}}^{(e)}({{\bf{r}}}),$$

13 $${\sigma }_{{{\mathcal{G}}},{c}}^{2}({{\bf{r}}})=\frac{1}{N-1}{\sum }_{e=1}^{N}{\left[{s}_{{c}}^{(e)}({{\bf{r}}})-{\bar{s}}_{{c}}({{\bf{r}}})\right]}^{2}.$$

The stochastic dispersion ${\sigma }_{{{\mathcal{G}}},c}({{\bf{r}}})$ provides a spatially resolved proxy for model uncertainty. Its magnitude, however, is not intrinsically calibrated and therefore cannot be interpreted directly as a statistically valid prediction interval. Calibration is performed in two stages. First, normalized residuals are computed on the held-out calibration set

14 $${z}_{i,{c}}({{\bf{r}}})=\frac{{s}_{i,{c}}^{{{\rm{true}}}}({{\bf{r}}})-{\bar{s}}_{i,{c}}({{\bf{r}}})}{{\sigma }_{{{\mathcal{G}}},i,{c}}({{\bf{r}}})+\epsilon },\qquad i=1,\ldots,{N}_{{{\rm{cal}}}},$$

where *ϵ* > 0 prevents numerical instability in regions of vanishing ensemble variance. A channel-specific scale factor, ${\lambda }_{{c}}={\mathrm{std}}\,\,\left({z}_{i,{c}}\right)$ is then used to rescale the predictive standard deviation

15 $$\sigma ^{{\prime} }_{{{\mathcal{G}}},i,{c}}({{\bf{r}}})={\lambda }_{{c}}\,{\sigma }_{{{\mathcal{G}}},i,{c}}({{\bf{r}}}).$$

Second, the absolute normalized calibration scores are defined as

16 $${a}_{i,{c}}({{\bf{r}}})=\frac{\left\vert {s}_{i,{c}}^{{{\rm{true}}}}({{\bf{r}}})-{\bar{s}}_{i,{c}}({{\bf{r}}})\right\vert }{\sigma ^{{\prime} }_{{{\mathcal{G}}},i,{c}}({{\bf{r}}})+\epsilon }.$$

Let ${q}_{c}^{1-\alpha }$ denote the split-conformal quantile of these scores for channel *c*, with *α* = 0.05. The resulting 95% prediction interval is

17 $${{\mathcal{I}}}_{c}^{1-\alpha}({{\mathbf{r}}})=\left[\, {\bar{s}}_c({{\mathbf{r}}}) - {q}_{c}^{1-\alpha}\sigma'_{{{\mathcal{G}}},{c}} ({{\mathbf{r}}}),{\bar{s}}_c({{\mathbf{r}}})+{q}_{c}^{1-\alpha} \sigma'_{{{\mathcal{G}}},{c}}({{\mathbf{r}}}) \right]$$

This construction retains the spatial variation supplied by the stochastic operator while using distribution-free conformal calibration to control empirical marginal coverage under exchangeability^7^.

**Fig. 3:**
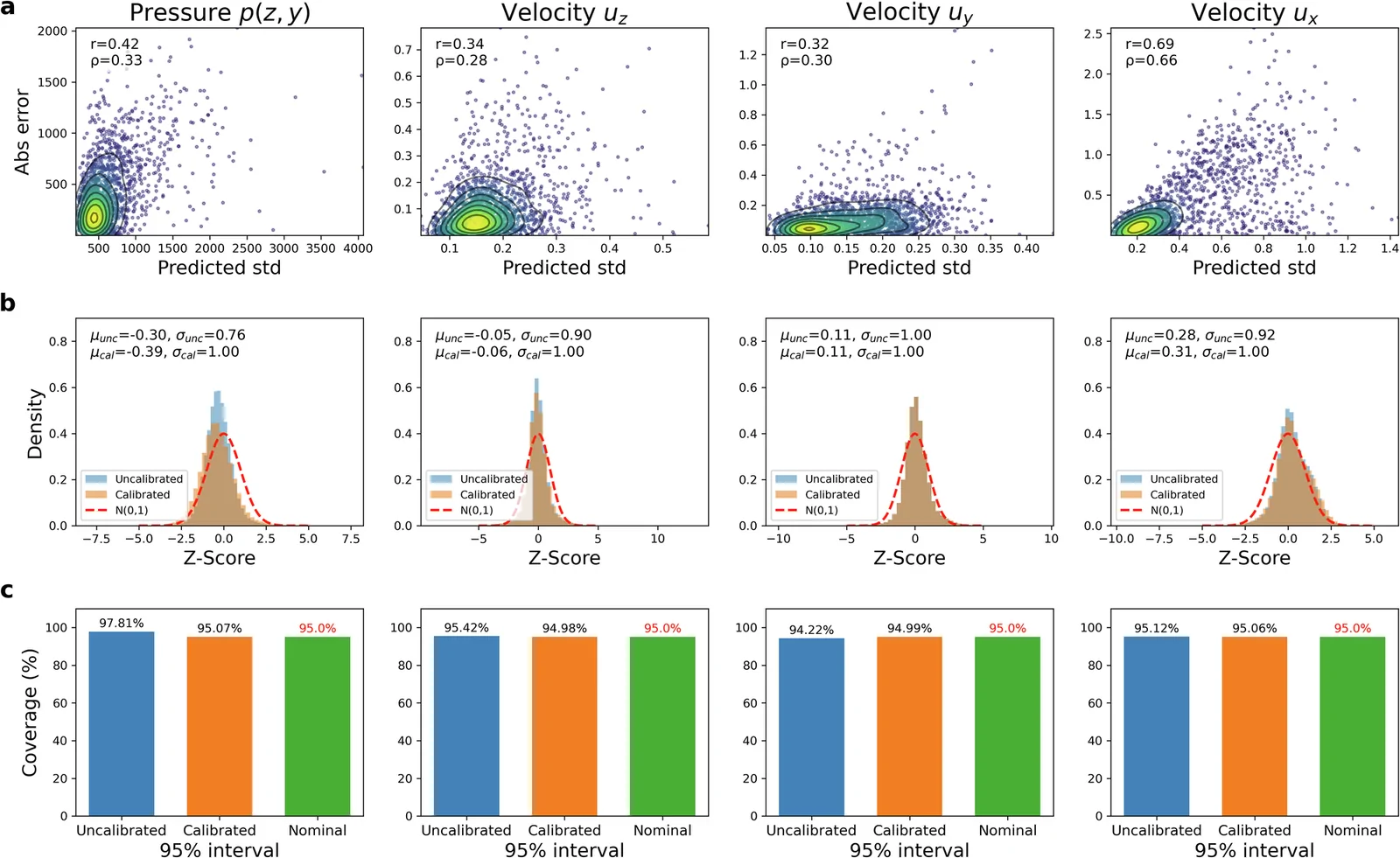
Uncertainty informativeness and calibration for heat-exchanger field reconstruction. **a** Predicted standard deviation versus absolute reconstruction error for pressure and the three velocity components, with density contours. Pearson *r* and Spearman *ρ* quantify the association between uncertainty magnitude and realized error. **b** Distributions of normalized residuals before and after calibration, compared with the standard normal density. The uncalibrated *z*-score distributions are narrower than ${{\mathcal{N}}}(0,1)$, indicating conservative raw MC-dropout intervals, whereas calibration brings their means and standard deviations close to 0 and 1, respectively. The normal density is included as a diagnostic reference and is not assumed by the conformal procedure. **c** Empirical coverage of nominal 95% prediction intervals before and after calibration. The uncalibrated intervals are conservative on pressure (97.8%) but close to nominal on the velocity channels (95.4%, 94.2% and 95.1%), so the miscalibration is channel-dependent rather than a uniform excess; conformal calibration brings all four within 0.25 percentage points of the 95% target.

Figure 3a examines uncertainty informativeness, namely, whether larger stochastic dispersion is associated with larger reconstruction error. Positive associations are observed across the four output channels, with the strongest relationship for *u*_*x*_ (Pearson *r* = 0.69, Spearman *ρ* = 0.66). Although the estimated uncertainty does not determine the pointwise error exactly, it provides a meaningful ranking of comparatively reliable and unreliable spatial predictions. The normalized-residual distributions in Fig. 3b show that the raw MC-dropout uncertainties are miscalibrated by channel rather than uniformly conservative. The normalized residual scale is below unity for pressure (0.77), *u*_*z*_ (0.90) and *u*_*x*_ (0.93), so the predicted standard deviations exceed the residual scale on those channels, but it is 1.00 for *u*_*y*_, where the raw spread is already correctly scaled. This is consistent with the uncalibrated 95% intervals over-covering on pressure (97.8%) while slightly under-covering on *u*_*y*_ (94.2%) (Fig. 3c). Channel-wise rescaling and conformal calibration reduce this excess width, bringing the normalized residuals close to unit scale and the empirical coverage close to the nominal 95% level. The calibrated intervals, therefore, satisfy two complementary requirements: their spatial variation is informative about reconstruction difficulty, and their aggregate coverage has a direct empirical interpretation. The calibration result applies to test conditions exchangeable with the calibration data and does not guarantee coverage under an arbitrary distribution shift. Within this scope, stochastic operator inference combined with conformal calibration provides quantitatively interpretable confidence bounds for fields reconstructed at locations where direct operational validation is unavailable. Having established both deterministic reconstruction accuracy and calibrated stochastic inference, we next compare MIMONet with alternative architectures in terms of nominal accuracy, model size, and robustness to degraded inputs.

#### Benchmarking against operator-learning baselines

Having established field-reconstruction accuracy across the three physical configurations and calibrated uncertainty for the heat-exchanger case, we next compare MIMONet with GeoFNO, NOMAD, and KCN in terms of nominal accuracy, model size, and robustness to degraded inputs. GeoFNO represents geometry-aware spectral operator learning, NOMAD provides a coordinate-conditioned neural-operator baseline, and KCN represents learned kriging on spatial graphs. All models were evaluated using the same case-wise training and test partitions, accessible input information, output fields, and channel-wise relative *ℓ*_2_ metric. Parameter counts vary across cases because the input branches and output dimensions are problem-dependent.

**Fig. 4:**
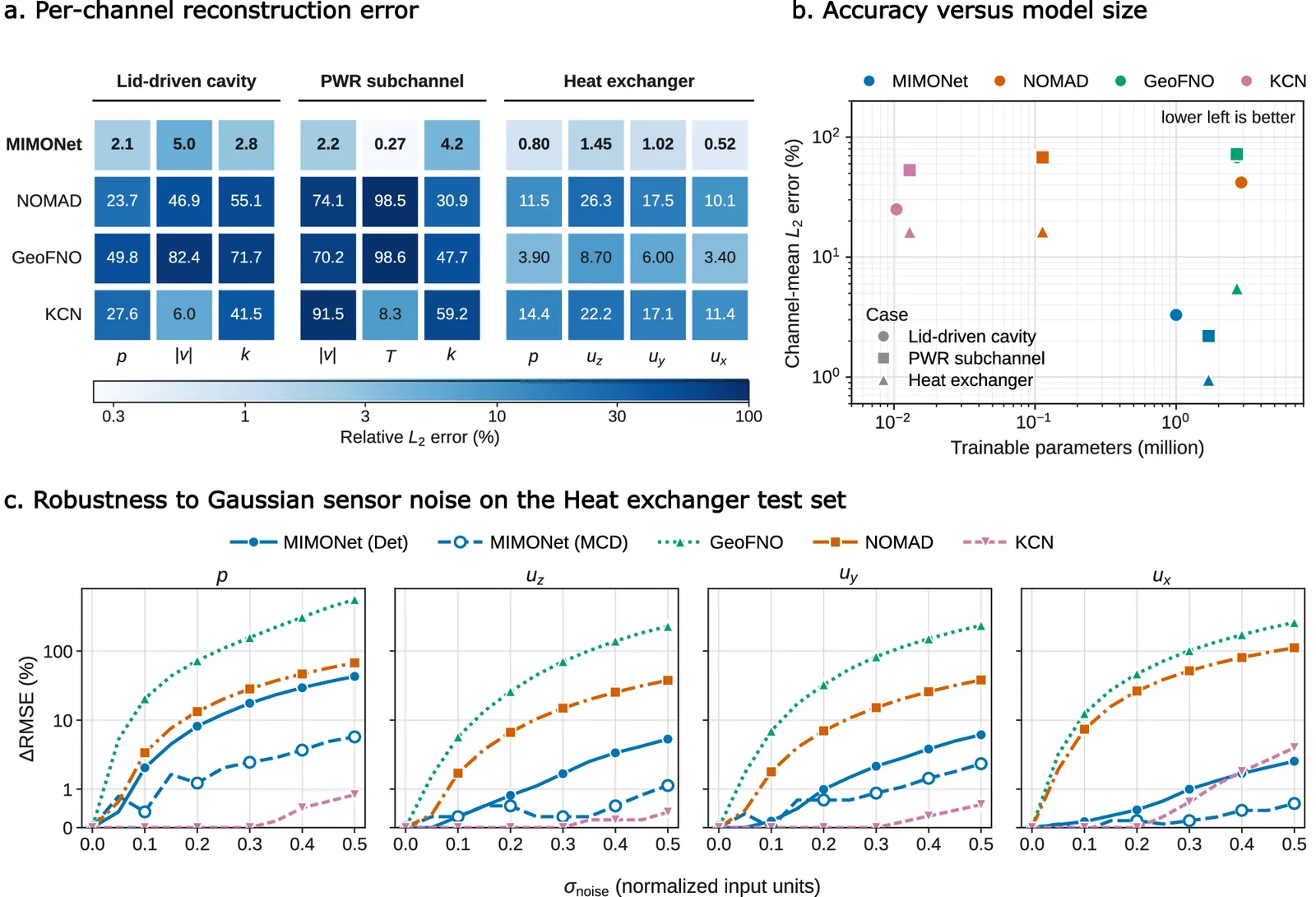
Benchmarking MIMONet against neural-operator and learned-interpolation baselines. **a** Mean relative *ℓ*_2_ error (%) for every reconstructed field in the lid-driven cavity, PWR subchannel and heat-exchanger test sets. The colour scale is logarithmic. **b** Channel-averaged error versus the number of trainable parameters; colour identifies the architecture and marker shape the case study. **c** Per-channel relative RMSE inflation over each model’s own noise-free baseline, under additive Gaussian noise applied to the normalized heat-exchanger inputs. All baselines use deterministic single-pass inference. MIMONet-MCD is the calibrated dropout-enabled variant of the same model, averaging *N* = 20 stochastic evaluations, and is shown to quantify the robustness gained through stochastic inference rather than as a separate architecture. The vertical axis is linear below 1% and logarithmic above.

MIMONet attains the lowest error for every reconstructed quantity in all three cases (Fig. 4a). Its channel-averaged errors are 3.3% for the lid-driven cavity, 2.2% for the PWR subchannel, and 0.95% for the heat exchanger. Relative to the strongest baseline in each case, these values correspond to reductions of approximately 7.6-fold, 24.1-fold, and 5.8-fold, respectively. The advantage is not limited to a particular physical quantity: MIMONet remains the most accurate method for pressure, temperature, velocity, and turbulent kinetic energy. The channel-resolved comparison also reveals an important distinction between isolated and coupled-field accuracy. KCN approaches MIMONet for velocity magnitude in the cavity (6.0% compared with 5.0%) and provides a comparatively low temperature error in the PWR subchannel (8.3%). However, its errors increase substantially for the remaining coupled quantities, reaching 27.6% and 41.5% for cavity pressure and turbulent kinetic energy and 91.5% and 59.2% for subchannel velocity and turbulent kinetic energy. GeoFNO and NOMAD exhibit similarly uneven performance across channels. In contrast, MIMONet maintains low errors across all jointly reconstructed outputs, indicating that its advantage arises from balanced multichannel reconstruction rather than exceptional performance on a single smooth field.

The accuracy differences cannot be explained by model size alone (Fig. 4b). MIMONet uses 1.0 million parameters for the cavity and 1.7 million for the subchannel and heat exchanger, compared with 2.7 million for GeoFNO. Despite its smaller parameter count, MIMONet reduces the channel-averaged error from 68.0% to 3.3% in the cavity, from 72.2% to 2.2% in the subchannel, and from 5.5% to 0.95% in the heat exchanger. Conversely, the substantially smaller NOMAD and KCN models do not provide a competitive accuracy-size trade-off. These results indicate that the performance gain is associated with the multimodal branch encoding, multiplicative latent fusion, and shared multichannel decoding rather than parameter count alone.

To examine whether this advantage persists under degraded measurements, Gaussian perturbations were applied to the concatenated heat-exchanger inputs

18 $$\widetilde{{{\boldsymbol{u}}}}={{\boldsymbol{u}}}+{{\boldsymbol{\eta }}},\qquad {{\boldsymbol{\eta }}} \sim {{\mathcal{N}}}\,\left({{\boldsymbol{0}}},{\sigma }_{{{\rm{noise}}}}^{2}{{\boldsymbol{I}}}\right),$$

where *σ*_noise_ ∈ [0, 0.5] is expressed in normalized input units. Robustness is measured through the channel-wise relative RMSE inflation

19 $${{\Delta }}{{\rm{RMSE}}}({\sigma }_{{{\rm{noise}}}})=100\frac{{{\rm{RMSE}}}({\sigma }_{{{\rm{noise}}}})-{{\rm{RMSE}}}(0)}{{{\rm{RMSE}}}(0)}\ (\%).$$

All architectures were evaluated on the same 310-sample heat-exchanger test set without retraining on noisy inputs. MIMONet-Det, GeoFNO, NOMAD, and KCN use one deterministic forward pass for each perturbed input. MIMONet-MCD uses the *N* = 20 stochastic evaluations. Because the MCD variant additionally performs posterior averaging, it is reported as an uncertainty-aware extension rather than as a directly capacity-matched single-pass baseline. At *σ*_noise_ = 0.5, deterministic MIMONet exhibits a channel-averaged RMSE inflation of ~14%, compared with ~64% for NOMAD and 319% for GeoFNO (Fig. 4c). Thus, among the deterministic architectures that propagate the perturbed branch inputs, MIMONet provides the lowest error inflation. The calibrated MIMONet-MCD variant further reduces the channel-averaged inflation to ~2.45%, with per-channel values of 5.71% for pressure and 1.13%, 2.33%, and 0.63% for *u*_*z*_, *u*_*y*_, and *u*_*x*_, respectively. This additional reduction reflects the benefit of stochastic averaging rather than a purely architectural single-pass advantage. The larger sensitivity of pressure is consistent with its globally coupled character, whereas the velocity components remain comparatively stable under the imposed perturbations. KCN displays an apparently small channel-averaged inflation of ~1.5%, but this behaviour does not imply superior sensing robustness. Its graph and edge weights are constructed from fixed mesh coordinates, so perturbations applied to the functional and scalar branch inputs are largely excluded from its inference pathway. The resulting insensitivity must therefore be considered together with its noise-free heat-exchanger error of 16.3%, compared with 0.95% for MIMONet.

To complement the finite-noise evaluation, we characterized the local sensitivity of MIMONet using an empirical Lipschitz proxy. For each output channel, *L*_99_ was defined as the 99th percentile of

20 $$\frac{\parallel {{{\mathcal{G}}}}_{\theta }({{\boldsymbol{u}}}+{{\boldsymbol{\delta }}})-{{{\mathcal{G}}}}_{\theta }({{\boldsymbol{u}}}){\parallel }_{2}}{\parallel {{\boldsymbol{\delta }}}{\parallel }_{2}}$$

over 1000 random perturbation directions at each of 20 magnitudes in normalized input space. The resulting values were approximately 0.88, 0.37, 0.64, and 1.02 for *p*, *u*_*z*_, *u*_*y*_, and *u*_*x*_, respectively. Thus, no large local amplification is observed over the sampled heat-exchanger test distribution, consistent with the controlled error growth under noisy inputs in Fig. 4c. These values are empirical quantiles over sampled inputs and perturbation directions, not global Lipschitz constants or formal stability guarantees; the complete sensitivity curves are provided in Supplementary Material. Taken together, the nominal and perturbed-input evaluations show that MIMONet consistently combines low multichannel reconstruction error with stable propagation of noisy boundary information into inaccessible interior fields.

#### Local out-of-distribution generalization

A virtual sensor may encounter operating conditions outside the range represented in its training data. We therefore evaluated whether the learned operator degrades gradually under moderate parameter extrapolation. For the heat-exchanger configuration, 50 additional ANSYS Fluent simulations were generated with the inlet velocity *u*_in_, inlet temperature *T*_in_, and wall heat-flux amplitude placed ~15% outside their respective training ranges. Twenty-five cases were sampled below the lower training boundary and 25 above the upper boundary. The geometry, solver configuration, convergence criteria, data-processing pipeline, and target plane at *x* = 0.789 were unchanged. All trained models were evaluated on these cases without retraining or fine-tuning.

**Fig. 5:**
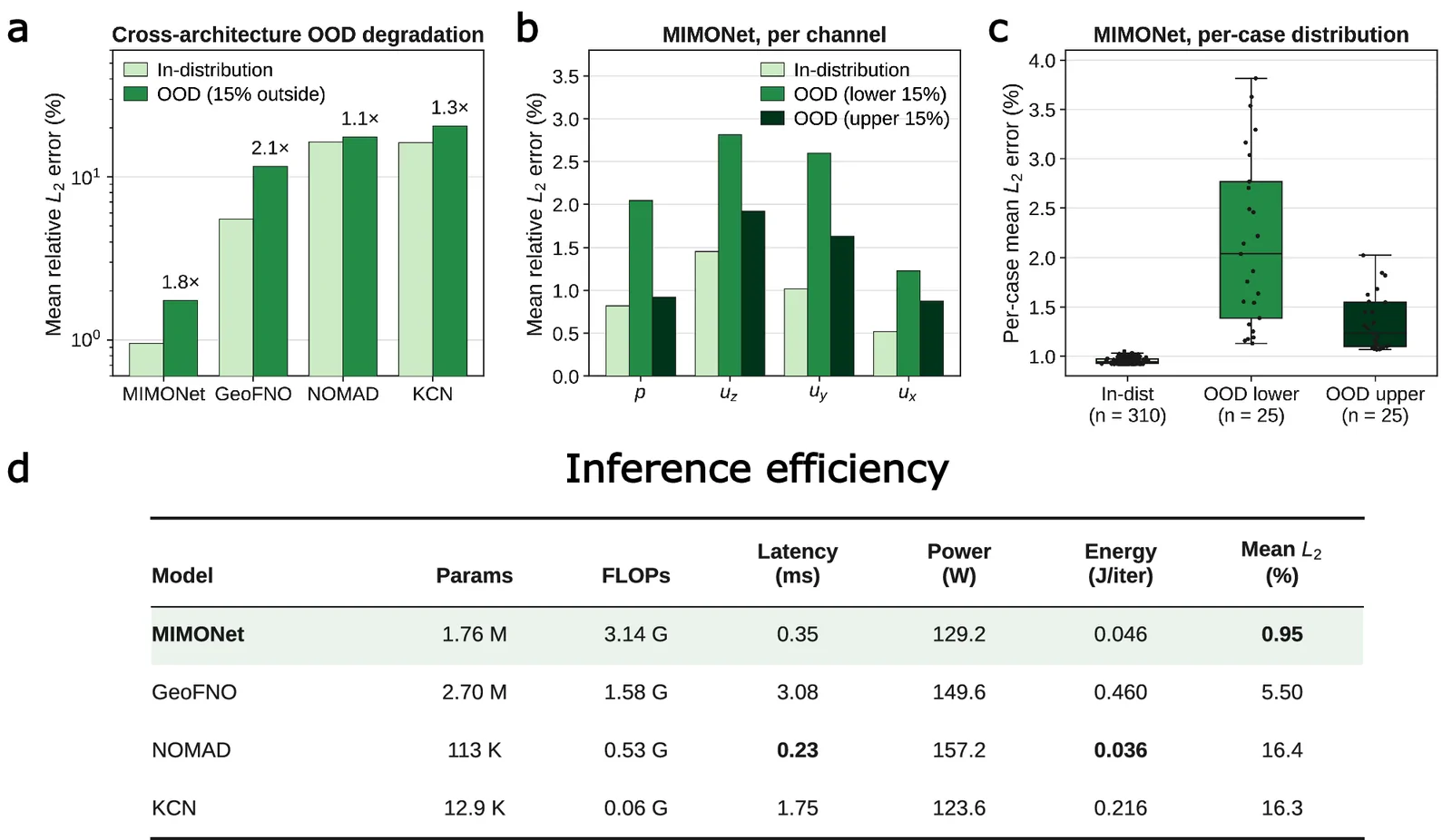
Local out-of-distribution generalization and inference efficiency on the heat exchanger. **a**–**c** MIMONet and the operator-learning baselines were evaluated on 50 additional CFD cases with operating parameters ~15% outside the training ranges (25 below and 25 above the training boundaries). **a** Channel-averaged relative *L*_2_ error under in-distribution and out-of-distribution conditions; labels give the OOD-to-ID error ratio. Note the logarithmic axis. **b** Per-channel MIMONet errors for the in-distribution test set and for operating conditions below and above the training boundaries. **c** Distribution of sample-wise MIMONet errors, with individual cases superimposed. Boxes show the median and interquartile range, and whiskers show the full range; the largest sample-wise OOD error is 3.82%. **d** Inference efficiency, profiled on a single NVIDIA H200 GPU with *B* = 1, *P* = 3977 query points, 1000 forward evaluations and float32 precision. Energy is calculated from total board power without idle-power subtraction. Bold entries mark the lowest latency, energy, and reconstruction error.

MIMONet’s channel-averaged relative *ℓ*_2_ error increases from 0.95% on the in-distribution test set to 1.75% on the OOD cases, corresponding to a 1.8-fold increase (Fig. 5a). The OOD errors are 1.48% for pressure, 2.37% for *u*_*z*_, 2.11% for *u*_*y*_, and 1.05% for *u*_*x*_ (Fig. 5b). Cases below the lower training boundary exhibit a larger channel-averaged error (2.17%) than cases above the upper boundary (1.34%), indicating asymmetric sensitivity to the direction of the parameter shift. MIMONet also retains the lowest OOD error among the evaluated architectures. GeoFNO increases from 5.5% to 11.6%, NOMAD from 16.4% to 17.6%, and KCN from 16.3% to 20.5%. MIMONet’s OOD error is therefore ~6.6–11.7 times lower than those of the three baselines. The relative degradation factors differ across architectures: GeoFNO exhibits the largest proportional increase, whereas NOMAD changes comparatively little but starts from a substantially higher in-distribution error.

The sample-wise distribution in Fig. 5c remains compact under the tested parameter shift. The largest OOD error is 3.82%, compared with 1.05% for the largest in-distribution error. Thus, the extrapolation cases increase both the typical error and its spread, but no abrupt loss of accuracy is observed within the evaluated 15% range. These results provide evidence of local parameter-extrapolation robustness under a fixed geometry, CFD solver, turbulence model, and preprocessing pipeline. They do not establish generalization to new geometries, different physical models, substantially larger parameter shifts, experimental data, or independently generated solver datasets. Cross-solver and experimental validation would therefore be required before interpreting this result as broad out-of-distribution generalization.

#### Real-time inference and energy cost

The operational value of virtual sensing depends not only on reconstruction accuracy but also on whether complete field estimates can be produced within the monitoring latency budget. We therefore profiled MIMONet, GeoFNO, NOMAD, and KCN on the heat-exchanger task using a single NVIDIA H200 GPU under an identical workload: batch size *B* = 1, *P* = 3977 query points, 1000 forward evaluations, and float32 precision. Ten warm-up evaluations were performed before measurement. Floating-point operations were measured using torch.profiler, and latency was recorded using time.perf_counter with torch.cuda.synchronize before and after each timed evaluation. Energy per inference was estimated as *E*_*i*_ = *P*_*i*_*t*_*i*_, where *P*_*i*_ is the mean NVML-reported board power and *t*_*i*_ is the measured inference latency. Because idle-board power was not subtracted, the energy values should be interpreted as workload-level comparative measurements on the same GPU rather than isolated model energy consumption. Reconstruction errors were evaluated on the same 310-sample heat-exchanger test set used in the section “Pressure–velocity reconstruction in a compact heat exchanger”.

MIMONet reconstructs all four fields at 3977 query points in 0.35 ms, with an estimated energy cost of 46 mJ per inference and a channel-averaged error of 0.95% (Fig. 5d). This latency is substantially below the millisecond-scale monitoring target considered here and is more than five orders of magnitude lower than the hundreds of seconds required by the corresponding high-fidelity CFD solution. The comparison concerns inference after offline training and does not include sensor acquisition, data transfer, or preprocessing latency. GeoFNO is the closest baseline in reconstruction accuracy, with a mean error of 5.5%, but requires 3.08 ms and 460 mJ per inference. Relative to GeoFNO, MIMONet is ~8.8 times faster and 10 times less energy-intensive while reducing the mean error by a factor of 5.8. The difference between the measured latency and reported FLOP counts also shows that FLOPs alone do not determine execution time; memory access, kernel structure, and hardware utilization contribute to the observed architecture-level performance. NOMAD attains the lowest latency (0.23 ms) and energy cost (36 mJ) but has a mean reconstruction error of 16.4%, ~17 times that of MIMONet. It therefore represents a different operating point on the accuracy–efficiency trade-off rather than being uniformly dominated by MIMONet. KCN combines the smallest parameter count and FLOP estimate with a latency of 1.75 ms, an energy cost of 216 mJ, and an error of 16.3%, illustrating that parameter count and nominal FLOPs do not translate directly into end-to-end inference efficiency.

The branch–trunk contraction used by MIMONet scales linearly with the number of query points, ${{\mathcal{O}}}(BPIm)$, where *I* is the latent width and *m* the number of output channels. This scaling supports dense field reconstruction while retaining sub-millisecond latency for the evaluated heat-exchanger mesh. Complementary profiling across NVIDIA A40, A100, H200, and GH200 GPUs is reported in Supplementary Fig. S9 and Supplementary Table S23, where MIMONet remains below 1 ms on the A40, A100 and H200 under the same workload; the slowest measurement anywhere is 1.17 ms on the GH200. The profiling results establish real-time computational feasibility for the evaluated hardware and query resolution. They should not be interpreted as a hardware-independent latency guarantee, because total deployment latency will additionally depend on sensor communication, preprocessing, batching, device scheduling, and the target computing platform.

### Real-world validation on measured and observation-constrained data

The preceding studies establish that operator-based virtual sensing reconstructs coupled fields under controlled, physically demanding conditions, with the reactor subchannel as the safety-critical case. Their reference fields are computed, so the assessment is complete but shares the assumptions of the model that produced it. We therefore turn to three datasets whose reference fields are instead measured or observation-constrained: two from physical instruments and one from an ocean reanalysis. Each retains interior values that can be withheld from the operator and recovered at evaluation, enabling direct comparison with the interpolation, extrapolation and learned sensing methods used in practice on such data. The three tasks progressively reduce direct observational support for the target. In the fuel-cell case, sparse measurements of both target quantities are available, but most interior locations are withheld. In the wind-tower case, the target lies above the highest input sensor and must be inferred by vertical extrapolation. In the ocean case, temperature and salinity are observed on disjoint sparse networks, whereas sea-surface height has no direct input observations and must be recovered through cross-modal coupling. This progression tests sparse interpolation, physical extrapolation, and zero-sensor cross-modal reconstruction within a common operator framework.

#### Sparse internal-field reconstruction in a fuel cell

A proton-exchange-membrane fuel cell (PEMFC) develops a spatially varying current-density field that governs local power generation, heat production, and degradation. Complete current-density maps can be obtained experimentally using a current-density-mapping (CDM) plate inserted between the cell layers, but this intrusive arrangement is unsuitable for routine operation. We therefore test whether the internal current-density and temperature fields can be reconstructed from measurements retained at only a small subset of spatial locations. We use the publicly available PEMFC dataset from the University of Seville^41^. The experiments were conducted on an ~50 cm^2^ parallel-serpentine cell under eight operating protocols comprising four gas inlet–outlet configurations and two load profiles: a polarization curve and a dynamic load cycle. Current density *J* was recorded on an 18 × 18 grid containing 324 locations, while temperature *T* was measured on a 9 × 9 grid, both at 1 Hz. Approximately 8000 measured states are retained. The temperature measurements are bilinearly mapped to the 18 × 18 current-density grid, and the states are partitioned into 70% training, 15% validation, and 15% test samples by a uniform random permutation of frame index. This is a random split rather than a chronological one, so a test frame can be temporally adjacent to a training frame; the reported errors accordingly characterize sparse-sensor reconstruction on operating states close to those seen in training, and are not a measure of temporal extrapolation.

Only 32 of the 324 grid locations are supplied to the model, corresponding to ~10% spatial coverage. Let ${{{\mathcal{S}}}}_{{{\rm{obs}}}}\subset {{{\Omega }}}_{{{\rm{grid}}}}$ denote this set of observed locations, with $| {{{\mathcal{S}}}}_{{{\rm{obs}}}}|=32$. The virtual-sensing operator is

21 $${{{\mathcal{G}}}}_{{{\rm{fc}}}}\left({\left\{{J}_{i},{T}_{i},{{{\bf{r}}}}_{i}\right\}}_{{{{\bf{r}}}}_{i}\in {{{\mathcal{S}}}}_{{{\rm{obs}}}}}\right)({{\bf{r}}})=\left[\begin{array}{c}J({{\bf{r}}})\\ T({{\bf{r}}})\end{array}\right],\qquad {{\bf{r}}}\in {{{\Omega }}}_{{{\rm{grid}}}}.$$

The complete fields are retained as reconstruction targets, but the remaining 292 locations receive no input observations. Because the sparse inputs are sampled from laboratory CDM maps, this experiment evaluates sparse reconstruction using measured physical data; it does not imply that the complete CDM apparatus is itself deployable.

A two-branch MIMONet is used. The first branch encodes the 32 observed current-density values together with Fourier representations of their coordinates, while the second encodes the corresponding temperature observations and coordinates. Their latent representations are fused multiplicatively, and a Fourier-feature trunk jointly decodes current density and temperature at arbitrary grid coordinates. MIMONet is evaluated against KCN and the classical IDW and *k*-nearest-neighbour (*k*NN) interpolants using identical sensor locations and data splits. KCN accepts scattered observations directly and provides a learned locality-based control. FNO is not treated as a native baseline because its spectral layers require a gridded input field; applying it to the 32 scattered observations would require a separate interpolation front end that is itself part of the sensing pipeline. Complete five-seed numerical results are reported in Supplementary Material.

**Fig. 6:**
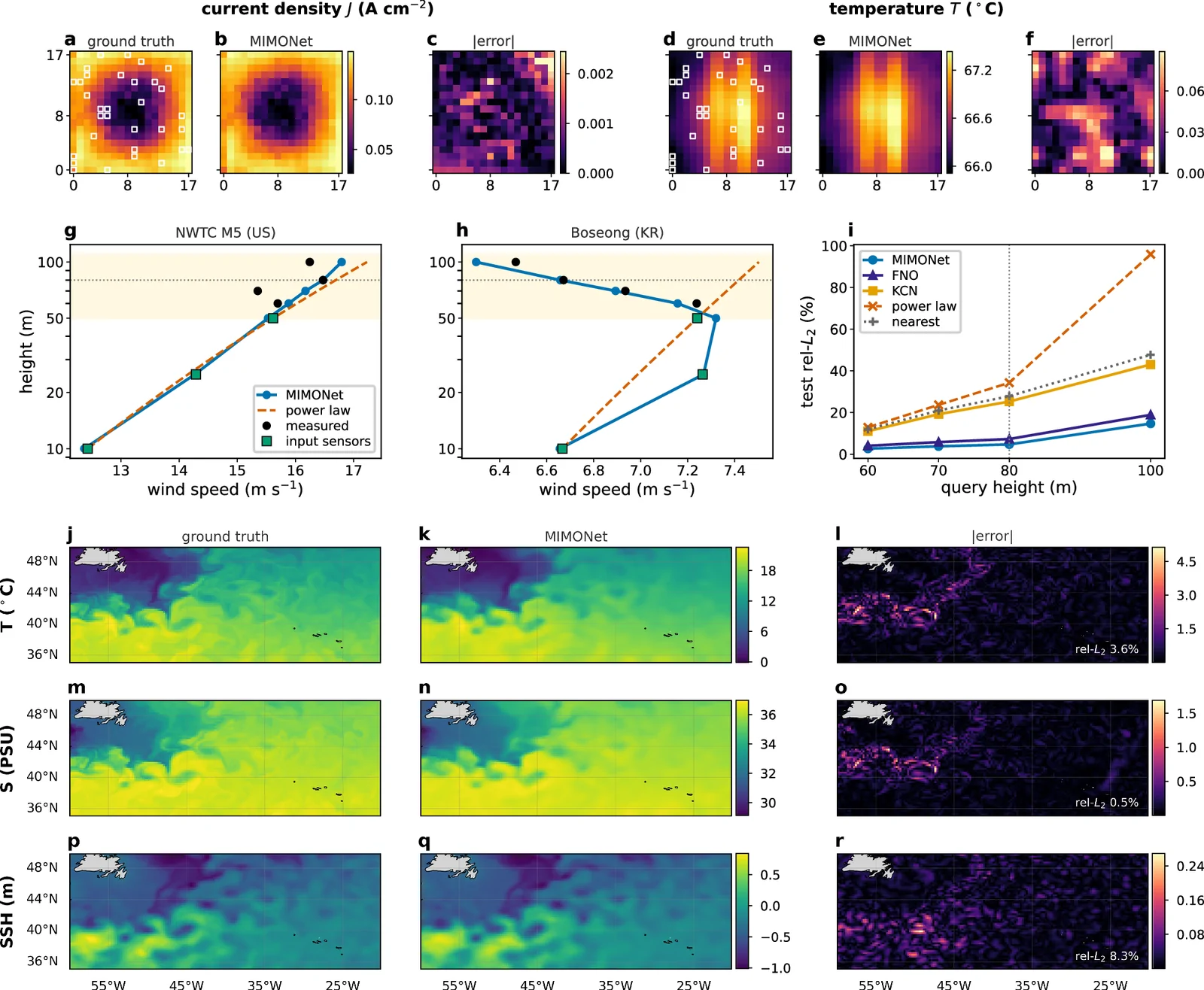
Operator-based virtual sensing on three real-world datasets. Each block reconstructs a field that no deployable instrument returns, from sparse observations of other quantities. **a**–**f** Proton-exchange-membrane fuel cell (Problem 4): current density *J* (**a**–**c**) and temperature *T* (**d**–**f**) for a held-out operating state, showing ground truth (**a,** **d**), MIMONet reconstruction (**b**, **e**) and absolute error (**c**, **f**). White squares mark the 32 of 324 observed segments of the 18 × 18 mapping grid; ground truth and reconstruction share a colour scale per channel. **g**–**i** IEA-Wind tall towers (Problem 5). **g**, **h** Vertical profiles on held-out timestamps: input sensors at 10/25/50 m (green), measurements (black), MIMONet (blue) and a power law fitted to the lowest and top sensors (dashed). The shaded band carries no input sensor. **i** Test relative *L*_2_ error against query height, five-seed mean; dotted line, hub height. **j**–**r** GLORYS12 North Atlantic (Problem 6): temperature *T* (**j**–**l**), salinity *S* (**m**–**o**) and sea-surface height (**p**–**r**) on a held-out day, from 400 temperature and 400 salinity sensors at disjoint locations. Ground truth (**j**, **m**, **p**) and reconstruction (**k**, **n**, **q**) share a colour scale; error panels (**l**, **o**, **r**) are inset with the per-field relative *L*_2_ error. Sea-surface height is unobserved in the input and is recovered from the learned temperature–salinity coupling. Land, grey.

Across five random seeds, MIMONet reconstructs the two-channel field with a channel-averaged relative *ℓ*_2_ error of 1.90 ± 0.51%, compared with 6.17 ± 1.96% for KCN, 11.67 ± 0.81% for IDW, and 11.99 ± 1.03% for *k*NN. Relative to IDW, the strongest classical baseline, this corresponds to an 84% reduction in channel-averaged error. The current-density field, the principal inaccessible quantity, is reconstructed with an error of 3.77 ± 1.02%, compared with 12.29 ± 3.93% for KCN, 23.10 ± 1.60% for IDW, and 23.74 ± 2.04% for *k*NN. Temperature is considerably smoother and is reconstructed with an error of 0.04 ± 0.00%. This exceptionally small temperature error should be interpreted in light of the original 9 × 9 measurements being bilinearly mapped to the finer grid; current density, therefore, provides the more discriminating assessment of sparse reconstruction. The representative result in Fig. 6a–f recovers the high-current peripheral region and the lower-current structure associated with the serpentine flow path. Residuals are concentrated near channel bends and regions containing sharper current-density gradients, whereas the smoother temperature field is reproduced almost uniformly. The measured-data experiment, therefore, shows that MIMONet reconstructs sparse internal energy-device fields substantially more accurately than classical interpolation and locality-based learned estimation.

#### Vertical extrapolation beyond wind-tower sensors

The second measured-data task introduces a different form of inaccessibility: the target lies above the highest available sensor. Wind-energy assessment requires wind speed near turbine hub height, typically around 80 m, whereas operational monitoring may rely on measurements from lower levels. Standard engineering methods use a power-law or logarithmic profile for this extrapolation, but their fixed-form assumptions can become inaccurate under atmospheric stratification, low-level jets, and site-dependent flow conditions. We therefore test whether an operator can infer the vertical profile above the instrumented range from lower-level measurements and contextual information. We use the BSC/IEA-Wind Tall Tower Dataset^42^, a quality-controlled collection of in-situ meteorological mast measurements. Seven towers containing near-surface measurements and instrumentation extending to at least 80 m are retained: Boseong (KR), Lindenberg (DE), the NWTC M2, M4, and M5 towers (US-CO), Hegyhátsál (HU), and Wallaby Creek (AU). The measurements are resampled to hourly resolution, yielding ~6 × 10^5^ vertical profiles spanning five climatic regions. Data from each tower is partitioned chronologically into 70% training, 15% validation, and 15% test samples. Only wind speeds at 10, 25, and 50 m, interpolated linearly from each tower's measured levels, are supplied as inputs. The operator reconstructs wind speed at query heights above the highest input sensor:

22 $${{{\mathcal{G}}}}_{{{\rm{wind}}}}\left({\{U({z}_{i}),{z}_{i}\}}_{{z}_{i}\in \{10,25,50\}{{\rm{m}}}},{{\bf{c}}}\right)(z)=U(z),\qquad z > 50\,{{\rm{m}}},$$

where ${{\bf{c}}}=[{{\rm{lat}}},{{\rm{lon}}},\sin {\rm {h}},\cos {\rm {h}}]$ contains the tower location and hour-of-day encoding. Performance is evaluated at 60, 70, 80, and 100 m; the 100 m evaluation uses the five towers with measurements reaching that height. Because no measurements above 50 m are included in the input, evaluation at these heights constitutes vertical extrapolation beyond the instrumented range.

A two-branch MIMONet is used. The first branch encodes the three lower-level wind-speed measurements together with their heights, while the second encodes the site and diurnal context vector **c**. These representations are fused multiplicatively, and a Fourier-feature trunk encodes the query height *z*. The context branch allows the operator to represent site- and time-dependent variations in vertical profile shape, although it does not explicitly measure atmospheric stability. MIMONet is compared with FNO, KCN, the power law, a two-point log-height extrapolation, and a nearest-sensor baseline that returns the wind speed measured at 50 m. Unlike the scattered-input fuel-cell case, FNO is directly applicable because the vertical wind profile forms an ordered one-dimensional sequence. The learned models are evaluated using the same chronological splits. They are not capacity-matched: MIMONet carries 207,617 parameters against 68,289 for FNO and 66,818 for KCN, so the two baselines are matched to each other while MIMONet is roughly three times larger. Complete results at all four query heights are reported in Supplementary Material.

At hub height (80 m), MIMONet achieves a relative *ℓ*_2_ error of 4.71 ± 0.15%, compared with 7.30 ± 1.16% for FNO, 25.28 ± 0.57% for KCN, 27.86% for the nearest-sensor baseline, 28.95% for the two-point log-height extrapolation, and 34.30% for the power law. Relative to the nearest-sensor estimate, the strongest classical baseline at this height, MIMONet, reduces the error by 83%. The error increases with extrapolation distance for every method. At 100 m, MIMONet attains 14.70 ± 0.59%, compared with 18.88 ± 1.00% for FNO, 43.06 ± 1.14% for KCN, 47.73% for the nearest-sensor estimate, 52.49% for the two-point log-height extrapolation, and 95.92% for the power law. The degradation of the fixed-form profiles reflects the application of idealized atmospheric-profile assumptions to heterogeneous measured conditions in which those assumptions need not hold.

The representative profiles in Fig. 6g, h show that MIMONet follows variations in measured profile shape within the uninstrumented region, including cases where the profile flattens or accelerates differently from the fitted power law. FNO is the strongest comparison method, confirming that operator learning is also effective when the input has a natural one-dimensional structure. MIMONet nevertheless retains lower error at every evaluated height (Fig. 6i), consistent with the benefit of jointly encoding the measured profile, site information, diurnal context, and continuous query coordinate. The results extend virtual sensing from spatial interpolation within an instrumented domain to reconstruction beyond the physical range of the available sensors.

#### Cross-modal ocean-state reconstruction from disjoint observations

The third task combines disjoint sensing, coupled multi-field reconstruction, and a target variable with no direct input observations. Temperature and salinity are supplied at different spatial locations, while sea-surface height (SSH) is withheld completely from the operator input. The model must therefore reconstruct the full temperature, salinity, and SSH fields from two sparse, non-overlapping observation networks. Unlike the preceding measurement-based cases, this experiment uses an observation-constrained ocean reanalysis and tests cross-modal reconstruction under a controlled sensor-withholding protocol.

We use the GLORYS12V1 global ocean reanalysis^43^, a 1/12^∘^-resolution data-assimilative product distributed by the Copernicus Marine Service. GLORYS12 combines an ocean circulation model with in-situ and satellite observations, including Argo profiles, satellite altimetry, and sea-surface-temperature retrievals. We extract potential temperature *T*, salinity *S*, and SSH over the North Atlantic (35–50°N, 60–20°W) and block-average the fields to ~0.25° resolution. The resulting dataset contains 730 daily snapshots over 9457 ocean cells, partitioned into 610 training, 60 validation and 60 test days by a random permutation of days. Because daily ocean fields are strongly autocorrelated, this protocol places most test days adjacent to training days; a forward-in-time split is therefore reported as a robustness check in Supplementary Material.

Two fixed, disjoint sensor networks are constructed: 400 temperature locations ${{{\mathcal{S}}}}_{T}$ and 400 salinity locations ${{{\mathcal{S}}}}_{S}$, with ${{{\mathcal{S}}}}_{T}\cap {{{\mathcal{S}}}}_{S}={{\emptyset}}$. No SSH values are supplied to the model. For day *d*, the cross-modal reconstruction operator is

23 $${{{\mathcal{G}}}}_{{{\rm{ocean}}}}\left({\{{T}_{i}^{(d)},{{{\bf{r}}}}_{i}\}}_{{{{\bf{r}}}}_{i}\in {{{\mathcal{S}}}}_{T}},{\{{S}_{j}^{(d)},{{{\bf{r}}}}_{j}\}}_{{{{\bf{r}}}}_{j}\in {{{\mathcal{S}}}}_{S}}\right)({{\bf{r}}})=\left[\begin{array}{c}{T}^{(d)}({{\bf{r}}})\\ {S}^{(d)}({{\bf{r}}})\\ {{{\rm{SSH}}}}^{(d)}({{\bf{r}}})\end{array}\right],\qquad {{\bf{r}}}\in {{{\Omega }}}_{{{\rm{ocean}}}}.$$

Temperature and salinity must therefore be reconstructed beyond their respective sparse networks, while SSH must be inferred entirely from relationships learned between the input *T* and *S* fields and the SSH target represented in the training data. Although SSH has zero coverage in the operator input, the GLORYS12 reference field itself is influenced by assimilated altimetry and should not be interpreted as independent in-situ ground truth.

For this case, we use MIMONet-NL, a nonlinear-decoder variant in which an MLP operating on the concatenated trunk and fused-branch representations replaces the linear contraction used in the preceding cases. One branch encodes the 400 temperature measurements and their Fourier-encoded coordinates, while the second encodes the 400 salinity measurements at the disjoint locations. The branch representations are fused multiplicatively, and a Fourier-feature trunk provides the query-coordinate representation for all three output fields. Temperature and salinity are learned as residual corrections to an IDW estimate, whereas SSH is predicted without an IDW prior because no SSH observations are supplied. MIMONet-NL is compared with KCN, IDW, *k*NN, and climatology. KCN accepts the two scattered networks directly and provides a learned locality-based comparison. IDW and *k*NN reconstruct the observed *T* and *S* channels independently. Because neither method can interpolate a variable with no observations, their SSH estimates are obtained by ridge regression from their reconstructed temperature and salinity fields. Climatology returns the grid-wise training mean and provides the strongest classical SSH predictor.

FNO is not treated as a native end-to-end baseline because the two scattered observation networks must first be mapped to a regular grid. As a reference, an IDW-gridded, capacity-matched FNO reconstructs all three channels with a channel-mean error of ~4.4% (3.10% for *T*, 0.47% for *S* and 9.58% for SSH), close to MIMONet-NL on every channel. The rasterization front end, not the accuracy, is why it is not treated as a native baseline here: it requires the scattered observations to be interpolated onto a regular grid before the operator sees them, which reintroduces exactly the spatial-interpolation step the operator formulation avoids.

Across five seeds, MIMONet-NL reconstructs the three fields with a channel-averaged relative *ℓ*_2_ error of 4.22 ± 0.06%, compared with 14.82 ± 0.15% for KCN, 23.35% for the IDW-based pipeline, 21.99% for the *k*NN-based pipeline, and 18.83% for climatology. Using the strongest applicable classical predictor for each channel gives an average error of 15.16%; MIMONet-NL reduces this error by 72%. For the directly observed modalities, MIMONet-NL attains errors of 3.10 ± 0.06% for temperature and 0.46 ± 0.01% for salinity, compared with the strongest classical results of 6.79 ± 0.32% and 0.99 ± 0.04%, respectively. KCN achieves 5.77 ± 0.23% for temperature and 0.86 ± 0.01% for salinity, showing that locality-based learning improves reconstruction when the corresponding target variable is represented among the inputs. The more distinctive result is SSH reconstruction. From temperature and salinity observations alone, MIMONet-NL achieves an SSH error of 9.11 ± 0.17%, compared with 37.7% for climatology, 37.84 ± 0.28% for KCN, and ~59% for ridge regression applied to the classically reconstructed *T* and *S* fields. This corresponds to a 4.1-fold reduction relative to climatology. KCN’s climatology-level SSH performance, despite its stronger results for temperature and salinity, indicates that local spatial reconstruction alone is insufficient for recovering the withheld channel. MIMONet-NL instead performs cross-modal field recovery, using the joint, nonlocal statistical and physical relationships among temperature, salinity, and SSH represented in the reanalysis data to reconstruct a channel that carries no direct observations. Figure 6j–r shows that the reconstructed fields preserve the Gulf Stream front and prominent mesoscale structures. Residuals are largest near frontal regions and sharp SSH gradients, where small spatial displacements produce comparatively large pointwise discrepancies. Within the stated reanalysis and sensor-withholding protocol, the results extend virtual sensing from sparse reconstruction and vertical extrapolation to recovery of a target field with no direct observations in the operator input.

## Discussion

This study establishes operator-based virtual sensing as a viable framework for reconstructing spatially continuous, coupled interior fields when direct instrumentation is sparse, physically restricted, or entirely unavailable for a target quantity. The reference-field studies demonstrate feasibility under controlled conditions across increasingly complex, reactor-relevant flow configurations, while the measurement-based and observation-constrained datasets show that the framework remains effective beyond solver-generated data. These latter applications span three distinct sensing regimes: reconstruction within a sparsely instrumented domain, extrapolation beyond the available sensor range, and cross-modal recovery of an unobserved target field. MIMONet reduces error by 72–84% relative to the strongest applicable classical baselines across these datasets. The most distinctive result is cross-modal field recovery: the reconstruction of sea-surface height, a field observed by no sensor, from temperature and salinity observations alone, achieving 9.11 ± 0.17% error compared with 37.7% for climatology. The overall evidence, therefore, supports operator learning not simply as a faster surrogate for numerical simulation, but as a general inference framework for recovering inaccessible physical states under progressively weaker observational support.

### Why the operator formulation enables virtual sensing

The observed performance follows from treating virtual sensing as an operator-learning problem rather than as pointwise interpolation or conventional field-to-field surrogacy. MIMONet assigns separate branch encoders to heterogeneous measurements, allowing functional inputs, scalar operating conditions, and observations collected on distinct sensor networks to be represented within a shared latent space. Multiplicative fusion introduces cross-modal interactions before decoding, while the coordinate-conditioned trunk evaluates the reconstructed fields at arbitrary query locations. The resulting map can therefore transfer information across measurement modalities and spatially separated regions instead of relying only on observations near each query point. This distinction becomes increasingly important as the task progresses from interpolation within a sparsely observed domain to extrapolation and zero-sensor reconstruction. FNO remains effective when the measurements possess a regular grid structure, as in the vertical wind profiles, but requires an additional gridding stage for scattered sensor networks. KCN accepts scattered observations directly and performs well for target quantities represented among its inputs, but its climatology-level SSH result indicates that its locality-based formulation does not recover the required cross-modal relationship under the present zero-sensor protocol. MIMONet-NL addresses the greater complexity of this task through nonlinear decoding while retaining the same multimodal, coordinate-conditioned operator structure. The formulation provides an inductive bias for coupled field reconstruction, but it does not explicitly enforce conservation laws, incompressibility, or other governing constraints; the physical consistency observed here is learned from the training data and evaluated empirically rather than guaranteed by the architecture.

### Real-time inference, uncertainty, and robustness

A second advantage of the operator formulation is that the computational cost of solving the inverse reconstruction problem is shifted to an offline training stage. Once trained, MIMONet evaluates the complete field through a single forward pass, reconstructing the heat-exchanger fields in 0.35 ms at an energy cost of 46 mJ on an H200 and retaining sub-millisecond inference on the evaluated A40, A100 and H200 platforms, with a worst case of 1.17 ms on the GH200. These measurements refer specifically to model inference; the latency of sensor acquisition, communication, preprocessing, and downstream decision-making would also contribute to the response time of a deployed monitoring system. Uncertainty estimation is therefore essential because rapid predictions are useful only when their reliability can be assessed. MC-Dropout provides spatially resolved predictive uncertainty, while conformal calibration adjusts the resulting intervals to attain the prescribed empirical coverage on exchangeable held-out data. The uncertainty maps generally widen in regions that are weakly constrained by the available measurements or contain sharper field gradients, providing an indicator of where reconstructions should be treated cautiously. Under the prescribed sensor-noise perturbations, the stochastic model exhibits <3% channel-mean RMSE inflation, with a maximum of 5.7% on the pressure channel, and is substantially less sensitive than its deterministic counterpart and the evaluated GeoFNO and NOMAD baselines. These results jointly support fast, uncertainty-aware inference under the tested conditions, although calibration and robustness under sustained distribution shift, sensor drift, or structured hardware failures remain to be established.

### Limitations and path to deployment

The present results define a promising but bounded validation envelope. The reactor-relevant studies use fields generated with a single CFD solver and RANS *k*–*ε* closure over fixed geometries and prescribed parameter ranges; performance under alternative solvers, turbulence closures, discretizations, or substantial geometric changes has not been established. Their targets are steady two-dimensional planes exported from three-dimensional simulations, rather than transient volumetric fields, and experimental interior thermal–hydraulic measurements were unavailable for independent validation. The three additional datasets provide important evidence beyond solver-generated training data, but they introduce their own qualifications. The PEMFC temperature field is mapped from a coarser measurement grid, the wind evaluation is limited to the retained towers and observed atmospheric conditions, and GLORYS12 is an observation-constrained reanalysis rather than independent ocean-sensor ground truth. Moreover, the experiments largely use fixed sensor configurations and do not fully represent sensor relocation, progressive calibration drift, correlated failures, or distribution shifts in operating conditions. Deployment, therefore, requires cross-solver verification, validation against controlled experiments, systematic testing under changing sensor networks, and evaluation of the complete monitoring loop rather than model inference alone. Extending the framework to spatiotemporal and geometry-aware operators would enable transient three-dimensional reconstruction across changing configurations, while physics-informed constraints and adaptive uncertainty calibration could improve consistency and reliability outside the training distribution. These developments would provide the necessary bridge from the demonstrated reconstruction capability to operational virtual sensing in energy and other safety-critical systems.

Taken together, the validated cases span a 50 cm^2^ electrochemical cell, atmospheric boundary layers at instrumented towers, and the North Atlantic basin, six orders of magnitude in spatial scale and four distinct governing-physics regimes. That a single operator formulation recovers inaccessible fields across this range, without architectural change and with the cross-modal recovery of an entirely unobserved channel, indicates that field-level observability is better viewed as a general inference capability than as a set of domain-specific techniques. Operator-based virtual sensing thus offers a route to real-time observability in precisely those systems where the states that matter most cannot be directly measured.

## Methods

This section describes the operator formulation, training and normalization procedures, uncertainty calibration and robustness evaluation, and the metrics and computational profiling used to assess reconstruction accuracy and real-time feasibility.

### Operator formulation and MIMONet architecture

Virtual sensing is formulated as learning an operator that maps available measurements and operating conditions to spatially continuous fields in regions where the target quantities cannot be measured directly. Let ${{\boldsymbol{u}}}=({u}_{1},\ldots,{u}_{n})\in {{\mathcal{U}}}$ denote *n* heterogeneous input modalities, which may include scalar operating parameters, sampled boundary functions, actuation histories, or sparse field measurements. The objective is to approximate

24 $${{\mathcal{G}}}:{{\mathcal{U}}}\to {L}^{2}({{\Omega }};{{\mathbb{R}}}^{m}),\qquad {{\mathcal{G}}}({{\boldsymbol{u}}})({{\bf{r}}})={{\bf{s}}}({{\bf{r}}})={\left[\begin{array}{ccc}{s}_{1}({{\bf{r}}})&\cdots &{s}_{m}({{\bf{r}}})\end{array}\right]}^{{\mathsf{T}}},$$

where **r** ∈ Ω is a query coordinate and **s**(**r**) contains the *m* coupled target quantities. Unlike fixed-output regression, the coordinate-conditioned formulation can be evaluated at arbitrary query locations within the represented physical domain, including locations not used as input sensors.

The proposed MIMONet implements Eq. (24) using modality-specific branch encoders, multiplicative latent fusion, and shared-latent multi-field decoding. Each input modality is encoded independently as

25 $${{{\bf{z}}}}_{i}={{{\mathcal{B}}}}_{i}({u}_{i};{{{\boldsymbol{\theta }}}}_{i})\in {{\mathbb{R}}}^{q},\qquad i=1,\ldots,n,$$

allowing inputs with different dimensions and physical meanings to be processed without first mapping them to a common grid. The branch representations are fused elementwise:

26 $${{\psi }}={\bigodot}_{i=1}^{n}{{{\bf{z}}}}_{i},\qquad {\psi }_{j}={\prod }_{i=1}^{n}{z}_{ij},\quad j=1,\ldots,q.$$

This multiplicative operation introduces interactions among the input modalities before spatial decoding and provides a separable inductive bias for learning coupled responses. It does not, by itself, impose the bilinear terms or conservation laws of the governing equations. A trunk network encodes the query coordinate, optionally after a Fourier-feature mapping, into channel-specific basis vectors ${{{\bf{t}}}}_{o}({{\bf{r}}})\in {{\mathbb{R}}}^{q}$. In the standard MIMONet decoder, the prediction for output channel *o* is

27 $${\widehat{s}}_{o}({{\bf{r}}})=\left\langle {{\boldsymbol{\psi }}},{{{\bf{t}}}}_{o}({{\bf{r}}})\right\rangle+{b}_{o},\qquad o=1,\ldots,m.$$

All target fields are therefore decoded from the same fused latent state ***ψ***, while the channel-specific basis vectors allow each output to retain its own spatial structure. Sharing the fused representation encourages coordinated multi-field prediction but does not guarantee incompressibility, conservation, or other forms of physical consistency; these properties are assessed empirically.

For the ocean reconstruction task, the greater complexity of predicting a channel with no direct observations motivated a nonlinear-decoder variant, denoted MIMONet-NL. It retains the same modality-specific branches, multiplicative fusion, and coordinate trunk, but replaces the linear contraction in Eq. (27) with

28 $$\widehat{{{\bf{s}}}}({{\bf{r}}})={{{\mathcal{D}}}}_{{{\rm{NL}}}}\left(\left[{{\boldsymbol{\psi }}};{{\mathcal{T}}}({{\bf{r}}})\right]\right),$$

where [ ⋅  ; ⋅ ] denotes concatenation and ${{{\mathcal{D}}}}_{{{\rm{NL}}}}$ is a multilayer perceptron. This decoder permits nonlinear interaction between the fused measurement representation and the query coordinate while preserving the common operator formulation used across the six evaluation problems. The formulation assumes that the supplied measurements and operating conditions contain sufficient information to identify the target fields over the evaluated data distribution. Observability is therefore assessed empirically through held-out reconstruction accuracy, uncertainty, and robustness experiments rather than asserted as a formal guarantee.

### Training, normalization, and baseline implementation

In all cases, models were trained to minimize the mean-squared error between predicted and reference fields over the training samples, query locations, and output channels:

29 $${{\mathcal{L}}}({{\boldsymbol{\theta }}})=\frac{1}{{N}_{{{\rm{tr}}}}Pm}{\sum }_{b=1}^{{N}_{{{\rm{tr}}}}}{\sum }_{p=1}^{P}{\sum }_{o=1}^{m}{\left[{\widehat{\widetilde{s}}}_{bpo}-{\widetilde{s}}_{bpo}\right]}^{2},$$

where ${N}_{{{\rm{tr}}}}$, *P*, and *m* denote the numbers of training samples, query points, and output channels, respectively. Model selection was performed using the validation loss, and the test partition was used only for final evaluation. MIMONet-NL was used only for the ocean reconstruction task; all other cases used the standard decoder in Eq. (27). Because the target fields have different physical units and numerical ranges, each output channel of the three simulation cases was scaled independently to [−1, 1]; for the three measured-data cases the targets were standardized with training-set statistics. For channel *o*, the transformation was

30 $${\widetilde{s}}_{bpo}=-1+2\,\frac{{s}_{bpo}-{s}_{o}^{\min }}{{s}_{o}^{\max }-{s}_{o}^{\min }+\varepsilon },\qquad {s}_{o}^{\min }={{\min }_{b,p}}\,{s}_{bpo},\quad {s}_{o}^{\max }={{\max }_{b,p}}\,{s}_{bpo},$$

where the extrema were calculated from the training partition only and *ε* prevents division by zero. The same transformation was applied unchanged to validation and test targets. Predictions were returned to physical units before calculating errors, physical residuals, or uncertainty intervals. Channel-wise scaling prevents fields with larger numerical magnitudes, such as pressure or temperature, from dominating the joint optimization objective. Learned baselines were trained using the same input information, target fields, and data partitions as MIMONet. Model capacities were matched where permitted by the respective architectures, and hyperparameters were selected using validation performance without access to the test partition. FNO was evaluated directly when the input possessed a regular ordered representation and through an explicitly reported gridding front end, where scattered observations required preprocessing. KCN was applied directly to scattered sensor inputs. Classical methods used the same retained sensor locations as the learned models and were fitted using only information available within the corresponding training or input protocol. Results from multiple initializations are reported as the mean and standard deviation, where applicable.

The models were implemented in PyTorch and trained on NVIDIA A40, A100, H200, or GH200 GPUs available through the DeltaAI computing system^45^. Architecture dimensions, parameter counts, optimizer settings, learning-rate schedules, batch sizes, stopping criteria, random seeds, and baseline-specific configurations are reported in Supplementary Material.

### Uncertainty calibration and robustness evaluation

Predictive uncertainty was estimated using Monte Carlo dropout (MC-Dropout). In inference, the trained weights were held fixed while dropout layers in the branch and trunk networks remained active with probability *p* = 0.10. For an input ***u***, *E* = 20 stochastic operator evaluations were performed. The predictive mean and variance at query coordinate **r** were estimated as

31 $$\widehat{{{\boldsymbol{\mu }}}}({{\bf{r}}})=\frac{1}{E}{\sum }_{e=1}^{E}{\widehat{{{\bf{s}}}}}^{(e)}({{\bf{r}}}),\qquad {\widehat{{{\boldsymbol{\sigma }}}}}^{2}({{\bf{r}}})=\frac{1}{E-1}{\sum }_{e=1}^{E}{\left[{\widehat{{{\bf{s}}}}}^{(e)}({{\bf{r}}})-\widehat{{{\boldsymbol{\mu }}}}({{\bf{r}}})\right]}^{2}.$$

The resulting spread provides a computationally tractable approximation of epistemic uncertainty induced by the stochastic subnetworks; it is not equivalent to exact Bayesian posterior inference and does not, by itself, represent all sources of measurement or model-form uncertainty.

Because raw MC-Dropout intervals need not attain their nominal coverage, they were calibrated using split conformal prediction. For output channel *o*, normalized nonconformity scores were calculated on a held-out calibration partition as

32 $${a}_{bpo}=\frac{\left\vert {s}_{bpo}-{\widehat{\mu }}_{bpo}\right\vert }{{\widehat{\sigma }}_{bpo}+\varepsilon },$$

where *ε* prevents instability at locations with negligible stochastic variance. Let ${q}_{o}^{(1-\alpha )}$ denote the finite-sample conformal quantile of the calibration scores for channel *o*. The calibrated interval at miscoverage level *α* is

33 $${{{\mathcal{C}}}}_{o}({{\bf{r}}})=\left[{\widehat{\mu }}_{o}({{\bf{r}}})-{q}_{o}^{(1-\alpha )}\left({\widehat{\sigma }}_{o}({{\bf{r}}})+\varepsilon \right),\ {\widehat{\mu }}_{o}({{\bf{r}}})+{q}_{o}^{(1-\alpha )}\left({\widehat{\sigma }}_{o}({{\bf{r}}})+\varepsilon \right)\right].$$

We used *α* = 0.05 to construct nominal 95% intervals. Coverage and interval width were evaluated only on the test partition. Under the standard exchangeability assumption between calibration and test samples, this procedure provides marginal finite-sample coverage rather than simultaneous coverage at every spatial location.

Robustness to sensor corruption was evaluated by perturbing the input measurements with zero-mean Gaussian noise. For modality *i*, the corrupted input was generated as

34 $${u}_{i}^{(\rho )}={u}_{i}+\rho \,{\sigma }_{{u}_{i}}{{\boldsymbol{\epsilon }}},\qquad {{\boldsymbol{\epsilon }}} \sim {{\mathcal{N}}}({{\bf{0}}},{{\bf{I}}}),$$

where ${\sigma }_{{u}_{i}}$ is the training-set standard deviation of the corresponding input and *ρ* controls the prescribed noise magnitude. The reference target fields were left unchanged, so the experiment measures sensitivity to input corruption rather than changes in the underlying physical state. Identical noisy inputs were supplied to deterministic MIMONet, stochastic MIMONet, GeoFNO, and NOMAD, and robustness was quantified through the relative increase in RMSE from the clean-input condition. The evaluated noise levels, calibration-set construction, conformal quantile convention, and channel-wise coverage results are reported in Supplementary materials.

### Evaluation metrics and computational profiling

Reconstruction accuracy was evaluated using the channel-wise relative *ℓ*_2_ error. For test sample *b* and output channel *o*, evaluated at query points ${\{{{{\bf{r}}}}_{p}\}}_{p=1}^{P}$, the error is

35 $${e}_{{{\rm{rel}}}}^{(b,o)}=\frac{{\left[{\sum }_{p=1}^{P}{\left({\widehat{s}}_{o}^{(b)}({{{\bf{r}}}}_{p})-{s}_{o}^{(b)}({{{\bf{r}}}}_{p})\right)}^{2}\right]}^{1/2}}{{\left[\mathop{\sum }_{p=1}^{P}{s}_{o}^{(b)}{({{{\bf{r}}}}_{p})}^{2}\right]}^{1/2}}.$$

Per-channel results are the arithmetic mean of ${e}_{{{\rm{rel}}}}^{(b,o)}$ over all test samples and are reported as percentages. When a single multi-field error is reported, the per-channel errors are averaged with equal weight so that fields with larger physical magnitudes do not dominate the aggregate. Results over repeated model initializations are reported as the mean and standard deviation. Robustness is evaluated through the percentage change in root-mean-square error relative to the corresponding clean-input condition, while uncertainty performance is assessed using empirical coverage and mean interval width.

Forward-pass latency and energy use were profiled on NVIDIA A40, A100, H200, and GH200 GPUs using the trained heat-exchanger model. Each evaluation used a batch size of one and *P* = 3977 query points, corresponding to one complete multi-field reconstruction. Gradient tracking was disabled, and ten preliminary iterations were used to stabilize memory allocation, kernel caching, and GPU clock states before *N* = 1000 timed evaluations. CUDA synchronization was performed immediately before and after every forward pass to prevent asynchronous kernel execution from biasing the measurement. Latency was measured using time.perf_counter(), and the reported value is the mean over the 1000 evaluations. Throughput was calculated as the reciprocal of mean latency.

GPU board power was sampled through NVIDIA’s NVML interface immediately before and after each synchronized forward pass and averaged to obtain *P*_*i*_. The estimated board energy associated with inference *i* was then calculated as *E*_*i*_ = *P*_*i*_*t*_*i*_, giving

36 $$\overline{E}=\frac{1}{N}\mathop{\sum }_{i=1}^{N}{P}_{i}{t}_{i}.$$

The reported power represents total board draw without idle-baseline subtraction; the resulting quantity should therefore be interpreted as an estimate of per-inference board energy rather than a direct measurement of incremental model energy. Throughout this work, real-time inference refers specifically to the operator forward-pass latency. Sensor acquisition, communication, preprocessing, and downstream decision-making were not included in the timing measurements and would contribute to the latency of a complete deployed monitoring loop.

## Supplementary information

Supplementary Information

Transparent Peer Review file

## Data Availability

The simulation data generated in this study: the transient lid-driven cavity, the pressurized-water-reactor subchannel and the compact heat-exchanger cases, have been deposited in the Zenodo database under accession code https://doi.org/10.5281/zenodo.21224751. The processed arrays for the three measured-data problems, the sensor draws and data splits for all five random seeds, the trained model checkpoints and the per-seed result files underlying every figure and table in this Article and its Supplementary Information are available at the same deposit. The proton-exchange-membrane fuel-cell data used in this study are available in the idUS repository of the Universidad de Sevilla under accession code 11441/160750, and are described in ref. ^46^; the accompanying experimental study is ref. ^41^. The tall-tower wind data used in this study are available in the EUDAT B2SHARE database under accession code https://doi.org/10.23728/b2share.136ecdeee31a45a7906a773095656ddb, and are described in ref. ^42^. The ocean reanalysis data used in this study are available in the Copernicus Marine Service database under accession code GLOBAL_MULTIYEAR_PHY_001_030; we use surface potential temperature, salinity and sea-surface height over 35–50°N, 60–20°W for the 730 daily fields spanning 1 January 2017 to 31 December 2018. Access to the source product requires free registration with the Copernicus Marine Service.

## Code availability

The full implementation of MIMONet, including source code, training and evaluation scripts, dataset configurations, the three preprocessed simulation datasets, and the trained model checkpoints for every reported result, is permanently archived and citable on Zenodo at https://doi.org/10.5281/zenodo.21224752^47^.
