# Supplementary material for: Virtual sensing to enable real-time monitoring of inaccessible locations & unmeasurable parameters
Source: Nat Commun. 2026 Sep 19;17:10976. doi: 10.1038/s41467-026-77463-7 (PMC13683354; doi:10.1038/s41467-026-77463-7)
Supplement: Supplementary file 1 [file 41467_2026_77463_MOESM1_ESM.pdf]

Supplementary Material

Kazuma Kobayashi[1], Farid Ahmed[1], Jaewan Park[2,3], Subhankar Sarkar[4], Seid Koric[2,3], Souvik Chakraborty[1,4,5], and Syed Bahauddin Alam[*1,3]

[1]Grainger College of Engineering, Nuclear, Plasma & Radiological Engineering Department, University of Illinois Urbana-Champaign, Urbana, IL, USA
[2]Grainger College of Engineering, Mechanical Science and Engineering Department, University of Illinois Urbana-Champaign, Urbana, IL, USA
[3]National Center for Supercomputing Applications, Urbana, IL, USA
[4]Department of Applied Mechanics, Indian Institute of Technology Delhi, New Delhi, India
[5]Yardi School of Artificial Intelligence, Indian Institute of Technology Delhi
[*]Corresponding author: alams@illinois.edu

Notation and Glossary

Symbols used throughout the main text and this Supplementary Material are summarised in Table S1 for convenience. Quantities defined only within a single section retain their context-specific meaning given there.

Contents

S1 Overview of the six problems **4**
S2 Mathematical formulation: the neural operator as a virtual sensor **5**
S2.1 Mixed-modal operator construction for coupled multiphysics 7
S2.2 Supplementary Note 1: permutation invariance of the multiplicative fusion 8
S3 Theoretical foundations **9**
S3.1 Standing assumptions, and where they hold 9
S3.2 Variational and functional-analytic interpretation 9
S3.3 The training objective as Tikhonov regularisation 10
S3.4 Universal approximation property 11
S3.5 Multiplicative fusion: empirical justification 11
S3.6 Error decomposition and measured convergence 12
S3.7 Mesh independence and continuous extension 12
S3.8 Comparison with kernel methods and Gaussian processes 13
S3.9 Low-rank structure of the learned operator 13
S4 Dataset preparation **14**
S4.1 Transient lid-driven cavity (Problem 1) 14
S4.2 PWR subchannel (Problem 2) 14
S4.3 Heat exchanger (Problem 3) 15
S4.4 Proton-exchange-membrane fuel cell (Problem 4) 16
S4.5 IEA-Wind tall towers (Problem 5) 17
S4.6 GLORYS12 North Atlantic reanalysis (Problem 6) 17
S5 Architectures and training configurations for all six problems **18**
S5.1 Two readout variants are used, and which one applies where 18
S5.2 Architectures 19
S5.3 Training configurations 19

S5.4 Normalisation, coordinate scaling and Fourier features . . . 20
S5.5 Baseline definitions . . . 21
S6 Baseline models . . . 21
S6.1 NOMAD (nonlinear manifold decoders) . . . 22
S6.2 KCN (Kriging Convolutional Networks) + NOMAD . . . 22
S6.3 GeoFNO + NOMAD . . . 23
S6.4 FNO (Problems 5 and 6) . . . 23
S7 Complete results for the simulation problems . . . 24
S8 Complete real-world results . . . 25
S8.1 Fuel-cell reconstruction across sensor coverage . . . 25
S8.2 Wind-profile reconstruction above the instrumented range . . . 27
S8.3 Ocean-state reconstruction from disjoint sparse networks . . . 28
S8.3.1 Other learned methods on the ocean problem . . . 29
S8.4 Forward-in-time robustness check for the ocean problem . . . 30
S9 Uncertainty, robustness and data efficiency . . . 32
S9.1 Conformal prediction for operator outputs . . . 33
S9.2 Stability under sensor noise: Lipschitz analysis . . . 34
S9.3 Sample complexity and convergence . . . 34
S9.4 Empirical Lipschitz characterisation . . . 36
S9.5 Structured (non-Gaussian) sensor-noise robustness . . . 36
S9.6 Gaussian sensor-noise inflation, tabulated . . . 38
S9.7 Local parameter extrapolation, tabulated . . . 39
S10 Computational complexity . . . 40
S10.1 Forward-pass complexity . . . 40
S10.2 Comparison with competing methods . . . 40
S10.3 Memory footprint . . . 40
S11 Real-time inference benchmarking across GPU architectures . . . 41

Table S1: **Notation and glossary.** Domain symbols are reused across the main text and Supplementary Material.

**Symbol**	**Definition**
Domains and spaces	
$\Omega \subset \mathbb{R}^d$	Spatial domain of the governing PDE; $d = 2$ throughout the case studies.
$\mathcal{Y} \subset \mathbb{R}^d$	Set of admissible query coordinates (interior + boundary).
$\mathcal{U} = \prod_{i=1}^{n} \mathcal{F}_i$	Heterogeneous input space; each $\mathcal{F}_i$ is a finite- or infinite-dimensional space of one input modality (scalars, function-valued profiles, time series).
$\mathcal{S} \subset L^2(\mathcal{Y}; \mathbb{R}^m)$	Output (solution) space of m-channel fields over $\mathcal{Y}$.
$\mathcal{X}$, $\mathcal{Y}_q$	Subsets of the domain where data are observed (sensors) and where reconstruction is requested (virtual queries) respectively. The disjoint case $\mathcal{X} \cap \mathcal{Y}_q = \varnothing$ is the cross-domain inference regime that Problems 5 and 6 instantiate; the symbols are used descriptively here and not in any derivation.
Operator and architecture	
$\mathcal{G} : \mathcal{U} \to \mathcal{S}$	True (continuous) nonlinear forward operator mapping boundary inputs to interior fields.
$\hat{\mathcal{G}}_\theta$	MIMONet surrogate operator parameterised by trainable parameters θ.
$\boldsymbol{u} = (\boldsymbol{u}_1, \ldots, \boldsymbol{u}_n)$	Multi-modal input drawn from $\mathcal{U}$.
$\mathbf{s}(\mathbf{r}) = \mathcal{G}(\boldsymbol{u})(\mathbf{r})$	Multi-channel output field at coordinate $\mathbf{r} \in \mathcal{Y}$.
$\phi_i : \mathcal{F}_i \to \mathbb{R}^I$	Branch encoder for modality i; produces a width-I latent vector.
$\tau : \mathbb{R}^d \to \mathbb{R}^{I \times m}$	Trunk encoder mapping a query coordinate to per-channel decoding weights.
$\psi_j(\boldsymbol{u}) = \prod_{i=1}^{n} [\phi_i(\boldsymbol{u}_i)]_j$	Fused branch latent (multiplicative aggregation; see Supplementary Note 1 in Section S2).
$\boldsymbol{\beta} \in \mathbb{R}^m$	Per-channel bias term applied after trunk–branch contraction.
$\hat{s}_o(\mathbf{r} \mid \boldsymbol{u}) = \sum_{j=1}^{I} \psi_j(\boldsymbol{u})\, \tau_{j,o}(\mathbf{r}) + \beta_o,\ o = 1, \ldots, m$	MIMONet's per-channel pointwise prediction at coordinate $\mathbf{r}$ (equivalent vector form $\hat{\mathbf{s}} = \sum_j \psi_j\, \boldsymbol{\tau}_j + \boldsymbol{\beta}$ with $\boldsymbol{\tau}_j = \tau_{\cdot,j,\cdot} \in \mathbb{R}^m$).
I	Branch–trunk latent width (256 for Problems 1–4, 128 for Problem 5, 384 for Problem 6; see Section S5).
$B,\ P$	Batch size and number of trunk query points per sample.
Training and inference	
N_{train}	Training-pool size used in the sample-complexity sweep.
n	Number of input modalities (e.g. $n = 1$ for LDC, $n = 2$ for Subchannel and HX). Distinct from n_{NOMAD} (NOMAD latent dimension) and from n_x (state-space dimension of classical observers).
σ_{noise}	Gaussian noise standard deviation in the standardised input space.
$\Delta\text{RMSE}(\sigma_{\text{noise}})$ (%)	Relative RMSE inflation at noise level σ_{noise} vs. the $\sigma = 0$ baseline: $100 \cdot [\text{RMSE}(\sigma) - \text{RMSE}(0)]/\text{RMSE}(0)$.
$L_{99,o}$, $L \equiv \max_o L_{99,o}$	Per-channel and global empirical Lipschitz proxies (99th percentile of $\Vert\Delta\mathcal{G}_\theta\Vert_2/\Vert\boldsymbol{\delta}\Vert_2$).
γ	Power-law exponent in the sample-complexity fit $\epsilon(N) \approx a\, N^{-\gamma}$. Distinct from the per-channel bias $\boldsymbol{\beta} \in \mathbb{R}^m$ above.
$\alpha,\ s_c$	α = conformal miscoverage level (e.g. $1-\alpha = 0.95$ for 95% intervals). $s_c = \text{std}(z_c)$ = per-channel post-hoc variance-calibration factor, where z_c is the vector of raw calibration-set z-scores $(\mathbf{s} - \hat{\mathbf{s}})/\sigma_G$ for channel c; renamed from α_c to avoid collision with the conformal level. Two unrelated uses of α occur locally in Section S5.5: the wind-shear power-law exponent and the ridge regularisation strength.
N (in MC-Dropout)	Number of stochastic forward passes per inference; $N = 20$ throughout.
Field-channel labels (per case)	
LDC: p, $\Vert\boldsymbol{v}\Vert$, k	Pressure, velocity-magnitude, turbulent kinetic energy.
PWR subchannel: $\Vert\boldsymbol{v}\Vert$, T, k	Velocity-magnitude, temperature, turbulent kinetic energy.
Heat exchanger: p, u_z, u_y, u_x	Pressure and three velocity components.
Constants used in the case studies	
$P_{\text{Sub}} = 1{,}733$	Trunk-mesh size for the PWR subchannel test set.
$P_{\text{LDC}} = 4{,}225$	Trunk-mesh size for the lid-driven cavity test set.
$P_{\text{HX}} = 3{,}977$	Trunk-mesh size for the heat-exchanger test set.

S1 Overview of the six problems

What this Supplementary is arguing, and what it is not. The claim under test throughout is the one set out in the Introduction, and it is a claim about deployment regime rather than about what is recoverable in principle. Classical state estimators can reconstruct spatially distributed fields from boundary data; what they do is propagate a state recursively under an explicit governing model and observation operator, on a prescribed grid or spatial basis whose coefficients are estimated, so that the governing model is part of online inference and evaluation at a new coordinate is tied to that representation. Established data-driven virtual sensors avoid the online model but return values at a set of locations fixed at training time. The regime addressed here is different from both: a map from sparse, heterogeneous, scalar- and function-valued measurements on a sensing region to a spatially continuous, coupled field over a reconstruction region that may be disjoint from it, evaluated at arbitrary query coordinates in a single amortised forward pass, with no PDE solved online and no retraining per operating condition. Where this Supplementary says that a classical estimator has no mechanism for a particular task, the statement is always about the estimator classes actually compared, which are spatial interpolants and fixed analytic profiles, and never a claim that no classical method could recover the field by other means. The comparisons that bear on that claim are against the classical estimators, and they are reported for Problems 4–6 in Section S8. They are not reported for Problems 1–3, and the reason is structural rather than an omission: those problems map boundary data to interior fields, so there are no samples of the output field to interpolate and no classical interpolant is defined. The simulation problems establish accuracy against other operator formulations on coupled multiphysics fields; the measured-data problems carry the comparison against what is deployed in practice. Comparisons among neural-operator architectures appear in several places, and their role is different: they document design decisions, establish that a reported effect is not an artefact of one particular architecture, and bound how much of a result depends on the choice of operator. On the measured-data problems those differences are small next to the gap between the operator formulation and the classical alternatives. On the simulation problems they are not: the simulation-case results in Section S7 span a factor of twenty between operator architectures, which is larger than any operator-versus-classical gap reported here. We do not read that spread as evidence of architectural superiority, because the baselines were not tuned per problem and carry very different capacities; it is reported because the main text quotes it, and it is not load-bearing. No conclusion in this work depends on a particular operator architecture being the best of its family.

Table S2 summarises what each problem asks of the operator, where the data comes from, and how it is divided. The three simulation problems (1–3) are generated with ANSYS Fluent and share a common structure: a boundary function together with scalar operating conditions is mapped to interior fields on a fixed CFD mesh. The three measured-data problems (4–6) differ from them in kind, not only in origin: their inputs are point sensors rather than boundary data, so the operator is asked to complete a partially observed state rather than to map boundary conditions to an interior. The three differ in how far that completion has to reach. In Problem 4 the sensors and the targets lie on the same grid, so the task is spatial infilling. In Problem 5 every target height lies above every input sensor, so the operator extrapolates beyond the instrumented range. In Problem 6 one of the three output channels is observed by no sensor of any kind, so the operator must infer it across modalities; that case is the sharpest form of the disjoint sensing and reconstruction domains this work addresses.

Table S2: **The six virtual-sensing problems.** P is the number of query coordinates at which the output field is evaluated. "Sensor regime" states what the operator receives at inference. Split sizes are given as training / validation / test in the units of the split column; the split protocol is stated because it differs across problems and is essential to reading the reported errors.

#	Problem	Inputs	Outputs	P	Split (unit; protocol)
1	Lid-driven cavity	Lid velocity $V(t)$, 90 time levels	p, $\|\boldsymbol{v}\|$, k	4,225	3,159/790/988 (samples; by trajectory)
2	PWR subchannel	$q(z)$ at 100 axial points; $T_{\text{in}}, v_{\text{in}}$	T, $\|\boldsymbol{v}\|$, k	1,733	3,200/800/1,000 (samples; random)
3	Heat exchanger	q_{wall} at 100 points; $T_{\text{in}}, u_{\text{in}}$	p, u_z, u_y, u_x	3,977	1,082/154/310 (samples; random)
4	PEM fuel cell	J and T at n_{obs} of 324 segments	J, T at all 324	324	5,600/1,200/1,200 (frames; random)
5	IEA-Wind towers	U at 10/25/50 m; site and hour	U at 60–100 m	7	421,013/90,219/90,222 (profiles; chronological per tower)
6	GLORYS12 ocean	T at 400 and S at 400 disjoint cells	T, S, SSH at all cells	9,457	610/60/60 (days; random)

Two entries in Tables S2 and S3 deserve emphasis because they qualify how the corresponding results

Table S3: **Data provenance and sensor regime.** "Observed fraction" is the share of the reconstruction domain that the operator sees at inference; it is not defined for Problems 1–3, whose inputs are boundary data rather than samples of the output field. Problem 5 reconstructs a one-dimensional profile, so its observed fraction refers to heights rather than to area.

#	Source	Sensor regime at inference	Observed fraction
1	ANSYS Fluent, transient RANS	Boundary actuation history only	—
2	ANSYS Fluent, steady RANS	Axial heat source and two inlet scalars	—
3	ANSYS Fluent, steady RANS	Wall heat flux and two inlet scalars	—
4	Segmented-cell laboratory measurements	n_{obs} point sensors on the mapping grid	1.9–50% of 324
5	BSC/IEA-Wind Tall Tower Dataset [1]	Three lower-level anemometers	3 of 7 heights
6	GLORYS12V1 reanalysis	800 point sensors, two disjoint networks	8.5% of 9,457

should be read, and both are documented in full in Section S4. The split protocol is not uniform: Problem 5 is split chronologically within each tower, Problem 1 is split at the level of complete trajectories so that a lid-velocity history cannot straddle the split, and Problems 2, 3, 4 and 6 are split by a random permutation of samples, frames or days. For Problems 4 and 6 in particular, where consecutive samples are temporally adjacent recordings of an autocorrelated system, a random split places test states close to training states; Section S8.4 quantifies what that means for the ocean problem. Separately, the observed fraction for Problem 4 refers to the current-density channel; the temperature channel is measured on a 9×9 grid and bilinearly upsampled to the 18×18 reconstruction grid, so it is not observed at 324 points in any configuration.

S2 Mathematical formulation: the neural operator as a virtual sensor

This section states formally the map that the Introduction describes in words, and fixes the notation used throughout. The formulation is not incidental to the claim: it is what makes the output a function rather than a vector of values at pre-chosen locations, and that is the property the deployment regime turns on.

A class of learning problems is considered in which the objective is to approximate nonlinear operators that map combinations of scalar and function-valued inputs to spatially distributed physical outputs. Such operators are encountered naturally in systems governed by partial differential equations (PDEs), particularly in thermal–fluid domains where boundary conditions, internal heat generation, and flow control determine the system response. Formally, the learned map is treated as an operator on function spaces rather than as a finite-dimensional regressor, ensuring that inference is defined for any admissible input function within a prescribed set.

This formulation encompasses mixed-modal input spaces, including both low-dimensional parametric variables (such as operating setpoints or control inputs) and high-dimensional function-valued fields (such as inlet velocity profiles or spatially varying heat sources). The objective is to learn an operator that maps these inputs to physically meaningful output fields (typically vector- or tensor-valued) distributed over a spatial domain of interest. These output fields may represent temperature, velocity, or turbulence kinetic energy, which are often difficult to measure directly in reactor-relevant environments. Crucially, by learning the *field-valued* response, the framework supports virtual sensing at *inaccessible locations* (points with no instrumentation) and recovery of *unmeasurable parameters*.

Across all scenarios, the inputs represent operational conditions derived from design specifications, actuator configurations, or sensor-informed system states. The learned operator thus enables high-resolution virtual diagnostics, reconstructing internal fields from sparse, heterogeneous, or boundary-only measurements. The emphasis is on *amortised* evaluation: once trained, the operator can be queried on arbitrary coordinate sets without retraining, decoupling inference cost from mesh resolution.

Let $D \subset \mathbb{R}^d$ denote the spatial domain and $\mathcal{Y} \subset D$ a lower-dimensional subset (e.g., a measurement or reconstruction plane). The forward operator is

$$\mathcal{G} : \mathcal{U} \to \mathcal{S}, \qquad \mathcal{G}(\boldsymbol{u})(\mathbf{r}) = \mathbf{s}(\mathbf{r}), \quad \mathbf{r} \in \mathcal{Y},$$

where $\boldsymbol{u} = (u_1, \ldots, u_n) \in \mathcal{U}$ with $\mathcal{U} = \prod_{i=1}^{n} \mathcal{F}_i$, and each $u_i \in \mathcal{F}_i$ is either $\mathbb{R}$ or a function space such as $L^2(\Omega_i)$ with $\Omega_i \subseteq \overline{D}$. The output $\mathbf{s}(\mathbf{r}) \in \mathbb{R}^m$ encodes m physical quantities on $\mathcal{Y}$, and the output space is $\mathcal{S} \subset L^2(\mathcal{Y}; \mathbb{R}^m)$. On compact subsets $\mathcal{K} \subset \mathcal{U}$, we seek $\hat{\mathcal{G}}$ that is uniformly close to $\mathcal{G}$ in the Bochner norm $\|\cdot\|_{L^2(\mathcal{Y};\mathbb{R}^m)}$, i.e., $\sup_{\boldsymbol{u}\in\mathcal{K}} \|\hat{\mathcal{G}}(\boldsymbol{u}) - \mathcal{G}(\boldsymbol{u})\| \le \varepsilon$.

In practice, inputs and outputs are observed on discrete grids. Let Π_U and Π_Y denote sampling operators for inputs and outputs, respectively. Given a dataset $\mathcal{D} = \{(\Pi_U \boldsymbol{u}^{(i)}, \Pi_Y \mathbf{s}^{(i)})\}_{i=1}^{N}$ drawn from an operating distribution on $\mathcal{K} \times \mathcal{S}$, we minimise empirical loss

$$\min_{\theta} \frac{1}{N} \sum_{i=1}^{N} \ell\Big(\Pi_Y \hat{\mathcal{G}}_\theta(\boldsymbol{u}^{(i)}),\ \Pi_Y \mathbf{s}^{(i)}\Big),$$

with ℓ a channel-wise L^2 loss. Because $\hat{\mathcal{G}}_\theta(\boldsymbol{u})$ is defined for any query set, evaluation on *inaccessible* coordinates is obtained by replacing Π_Y with arbitrary point sets.

To approximate the nonlinear operator $\mathcal{G} : \mathcal{U} \to \mathcal{S}$, a mixed-input, mixed-output branch–trunk architecture (MIMONet) is employed. This design accommodates both scalar and function-valued inputs and produces multichannel fields at arbitrary query locations, thereby aligning the learning task with the operator viewpoint. The representation is resolution-invariant: the same parameters decode fields on different meshes simply by changing the set of query coordinates.

Each input component $u_i \in \mathcal{F}_i$ is first encoded into a fixed-width latent vector by a feedforward encoder $\phi_i : \mathcal{F}_i \to \mathbb{R}^I$. The resulting latents are then fused element-wise to form a single representation

$$\psi_j = \prod_{i=1}^{n} [\phi_i(u_i)]_j, \quad j = 1, \ldots, I.$$

This multiplicative fusion preserves permutation invariance across modalities and yields a compact, modality-agnostic summary of the inputs. Physically, the product structure serves as a low-rank surrogate for multiplicative couplings in the governing PDEs (e.g., convective transport), encouraging a shared latent ψ that is decoded into all output channels through channel-specific basis vectors at the trunk's final layer (i.e. the channels share the latent but each has its own linear projection; there is no cross-channel mixing in the decoder output).

Spatial dependence is introduced through a trunk network that maps pointwise queries $\mathbf{r} \in \mathbb{R}^d$ to decoding weights, $\tau : \mathbb{R}^d \to \mathbb{R}^{I\times m}$. For batch size B and P query points per sample, the trunk output is $T \in \mathbb{R}^{B\times P\times I\times m}$ and the fused branch vector is broadcast as $\boldsymbol{\psi} \in \mathbb{R}^{B\times I}$. The multichannel field $\mathbf{s} \in \mathbb{R}^{B\times P\times m}$ is obtained by contracting over the latent index with a broadcasted per-channel bias $\boldsymbol{\beta} \in \mathbb{R}^m$,

$$[\mathbf{s}]_{bpo} = \sum_{j=1}^{I} \psi_{bj}\, T_{bpjo} + \beta_o, \qquad b = 1..B,\ p = 1..P,\ o = 1..m,$$

which provides a location-aware decoding of the fused representation while preserving multimodal conditioning. In practice, branch and trunk modules are implemented as fully connected networks with a shared latent width I, and the bias term is broadcast over batch and query points. The computational complexity of a forward pass scales as $\mathcal{O}(BPIm)$ for the branch–trunk contraction, so latency is linear in the number of queried coordinates; this underpins real-time evaluation on dense grids. Because $\tau(\mathbf{r})$ parameterises a continuous coordinate map, evaluation at inaccessible points (e.g., shielded core interiors or near-wall regions without sensors) is effected by querying those coordinates directly, without retraining or regridding. This admissibility of arbitrary query coordinates is a structural property of the branch–trunk formulation; the reconstruction accuracy attained at a queried coordinate remains governed by the local density of training nodes and the spatial regularity of the target field.

Operator-Based Virtual Sensing. This work applies operator learning to virtual sensing, reframing the task from pointwise regression between sensor readings ($f : \mathbb{R}^m \to \mathbb{R}^k$) to learning continuous operators that map boundary data to interior fields ($\mathcal{G} : \mathcal{U} \to L^2(\Omega; \mathbb{R}^m)$). Virtual sensing has traditionally been formulated as point-wise regression: given observed sensor values $\mathbf{x}_{\text{obs}} \in \mathbb{R}^m$, predict unobserved values $\mathbf{x}_{\text{unobs}} \in \mathbb{R}^k$ via a learned map $f_\theta : \mathbb{R}^m \to \mathbb{R}^k$. This formulation *can limit observability to pre-defined sensor*

locations and may not readily recover spatially continuous fields in inaccessible regions. We demonstrate that reconstructing coupled physical fields from sparse boundary measurements can be effectively addressed through operator learning in function space, enabling inference beyond fixed sensor locations.

We consider nonlinear operator learning problems where the goal is to approximate the solution operator $\mathcal{G} : \mathcal{U} \to \mathcal{S}$ that maps boundary functionals (e.g., inlet velocity profiles, wall heat flux distributions) to spatially resolved, physically coupled output fields distributed over domains where direct measurement is impossible. In safety-critical thermal–fluid systems, multiple physical fields, namely pressure p, velocity $\mathbf{v}$, temperature T, turbulence kinetic energy k, evolve jointly according to conservation laws (mass, momentum, energy) that enforce *tight nonlinear coupling.* The Navier–Stokes momentum equation contains the advective term $(\mathbf{v} \cdot \nabla)\mathbf{v}$, which couples velocity to itself bilinearly; energy transport includes the convection term $(\mathbf{v} \cdot \nabla)T$, coupling velocity and temperature multiplicatively. A surrogate that treats these fields independently (learning separate maps $u \mapsto p$, $u \mapsto \mathbf{v}$, $u \mapsto T$) may not enforce conservation constraints in regions lacking direct measurements. Accordingly, the learned map can be framed as an operator $\mathcal{G} : \mathcal{U} \to \mathcal{S}$ between infinite-dimensional function spaces, where $\mathcal{S} = L^2(\mathcal{Y}; \mathbb{R}^m)$ represents the *joint solution manifold* of all coupled fields. This operator viewpoint enables inference at arbitrary coordinates $\mathbf{r} \in \mathcal{Y}$ (including inaccessible regions) and supports inter-field physical consistency by learning continuous mappings from boundary data to interior fields.

To clarify the distinction, consider the prototypical virtual sensing task: given boundary measurements $\boldsymbol{u} = (q_{\text{wall}}, T_{\text{in}}, v_{\text{in}})$ (wall heat flux, inlet temperature, inlet velocity), reconstruct the interior pressure field $p(\mathbf{r})$, velocity field $\mathbf{v}(\mathbf{r})$, and temperature field $T(\mathbf{r})$ throughout a reactor subchannel where $\mathbf{r} \in \Omega_{\text{hidden}}$ is physically inaccessible to sensors.

Regression Formulation (Traditional Virtual Sensing): Train a model $f_\theta : \mathbb{R}^{n_{\text{in}}} \to \mathbb{R}^{n_{\text{out}}}$ on paired observations $(\boldsymbol{u}^{(i)}, \mathbf{y}^{(i)})$ where $\mathbf{y}^{(i)}$ contains sensor values at fixed locations. *Limitations:*

1. **Fixed output locations**: The output dimension n_{out} is fixed during training. To predict at a new location, the model must be retrained.
2. **Independent physical coupling**: Independent regressors for p, $\mathbf{v}$, T may predict velocity fields with $\nabla \cdot \mathbf{v} \neq 0$ (violating incompressibility) or pressure gradients inconsistent with momentum balance.
3. **No function-space generalisation**: Changing the mesh resolution or domain geometry requires new training data and model retraining.

Operator Formulation (This Work): Learn the solution operator $\mathcal{G} : \mathcal{U} \to L^2(\Omega; \mathbb{R}^m)$ that maps boundary functionals to the *entire coupled solution field.* Given $\boldsymbol{u}$, the operator returns $\mathcal{G}(\boldsymbol{u}) : \Omega \to \mathbb{R}^m$ such that $\mathcal{G}(\boldsymbol{u})(\mathbf{r}) = (p(\mathbf{r}), \mathbf{v}(\mathbf{r}), T(\mathbf{r}), k(\mathbf{r}))$ aims to satisfy the governing PDEs in Ω. *Capabilities enabled:*

1. **Query-time evaluation at any $\mathbf{r} \in \Omega$**: The operator $\mathcal{G}(\boldsymbol{u})$ is a continuous function, evaluable at coordinates unseen during training, including inaccessible regions.
2. **Physical coupling via shared latent manifold**: All output channels are decoded from a single latent representation $\boldsymbol{\psi}$ that encodes the coupled PDE state, which can help enforce consistency.
3. **Mesh-independent inference**: once $\mathcal{G}$ is learned, evaluation at a different coordinate set on the same geometry requires no retraining, because the trunk accepts any $\mathbf{r} \in \mathcal{Y}$. This is a structural property of the parameterisation. We do not claim transfer to a different geometry, which is not evaluated in this work.

This operator-based approach addresses key limitations of fixed-location regression. Virtual sensing in inaccessible reactor internals requires reconstructing fields at arbitrary locations; the operator formulation provides a natural framework for this task through continuous function approximation.

S2.1 Mixed-modal operator construction for coupled multiphysics

The operator $\mathcal{G} : \mathcal{U} \to \mathcal{S}$ must accommodate *heterogeneous boundary data*: scalar parameters (e.g., inlet temperature $T_{\text{in}} \in \mathbb{R}$), function-valued distributions (e.g., wall heat flux $q(\mathbf{r}) \in L^2(\partial\Omega)$), and discrete actuation signals (e.g., time-varying lid velocity $V(t) \in \mathbb{R}^{N_t}$). The input space is thus a product $\mathcal{U} = \prod_{i=1}^{n} \mathcal{F}_i$ where each $\mathcal{F}_i$ may be finite- or infinite-dimensional. The objective is to learn $\mathcal{G}$ such that the output

$\mathbf{s}(\mathbf{r}) = \mathcal{G}(\boldsymbol{u})(\mathbf{r})$ represents the *joint solution* of all coupled fields. These output fields represent quantities that are either *unmeasurable* (turbulent kinetic energy k, pressure gradients ∇p, wall shear stress) or *inaccessible* (interior velocity and temperature distributions in confined reactor subchannels). The operator formulation supports two properties that a fixed-grid regression model does not provide directly: **(i)** evaluation at arbitrary $\mathbf{r} \in \mathcal{Y}$, including uninstrumented regions, without retraining or re-meshing, and **(ii)** differentiability of the decoded field in the query coordinate, so that derived quantities such as ∇p or $\nabla \cdot \mathbf{v}$ are available in principle by differentiating the trunk. We state (ii) as a structural property and not as a demonstrated result: no derived quantity is computed or scored in this work. The shared latent decoder couples the output channels through a common representation, but it does not enforce conservation laws, and the consistency observed in the results is learned from data rather than guaranteed by the architecture.

Across all evaluation scenarios, the inputs represent operational conditions derived from design specifications, actuator configurations, or sensor-informed system states. The learned operator thus enables high-resolution virtual diagnostics, reconstructing internal fields from sparse, heterogeneous, or boundary-only measurements. Key to this formulation is amortisation: once trained, the operator supports query-time evaluation on arbitrary coordinate sets, decoupling inference latency from mesh resolution or geometry.

S2.2 Supplementary Note 1: permutation invariance of the multiplicative fusion

The multiplicative branch fusion used in MIMONet is invariant under reordering of the branch encoders. We give a formal statement and proof here. Let $\boldsymbol{u} = (\boldsymbol{u}_1, \ldots, \boldsymbol{u}_n) \in \mathcal{U} = \prod_{i=1}^{n} \mathcal{F}_i$ denote the heterogeneous multi-modal input vector and let $\phi_i : \mathcal{F}_i \to \mathbb{R}^I$ be the corresponding branch encoders, each producing a width-I latent vector. The fused latent is

$$\psi(\boldsymbol{u}) \;:=\; \big(\psi_1(\boldsymbol{u}), \ldots, \psi_I(\boldsymbol{u})\big) \in \mathbb{R}^I, \qquad \psi_j(\boldsymbol{u}) \;=\; \prod_{i=1}^{n} \big[\phi_i(\boldsymbol{u}_i)\big]_j, \quad j = 1, \ldots, I. \tag{1}$$

Theorem 1 (Permutation invariance of the multiplicative fusion)**.** *For every permutation $\sigma \in \mathfrak{S}_n$ of the modality index set $\{1, \ldots, n\}$ and every input $\boldsymbol{u} \in \mathcal{U}$,*

$$\psi\big(\boldsymbol{u}_{\sigma(1)}, \ldots, \boldsymbol{u}_{\sigma(n)};\, \phi_{\sigma(1)}, \ldots, \phi_{\sigma(n)}\big) \;=\; \psi\big(\boldsymbol{u}_1, \ldots, \boldsymbol{u}_n;\, \phi_1, \ldots, \phi_n\big).$$

That is, simultaneously reordering the inputs and the corresponding branch encoders does not change the fused latent.

Proof. Fix any $j \in \{1, \ldots, I\}$. By definition (1) applied to the permuted tuple,

$$\psi_j\big(\boldsymbol{u}_{\sigma(1)}, \ldots, \boldsymbol{u}_{\sigma(n)};\, \phi_{\sigma(1)}, \ldots, \phi_{\sigma(n)}\big) \;=\; \prod_{i=1}^{n} \big[\phi_{\sigma(i)}(\boldsymbol{u}_{\sigma(i)})\big]_j.$$

For each i, the scalar $[\phi_{\sigma(i)}(\boldsymbol{u}_{\sigma(i)})]_j \in \mathbb{R}$; ordinary multiplication of real numbers is commutative, so

$$\prod_{i=1}^{n} \big[\phi_{\sigma(i)}(\boldsymbol{u}_{\sigma(i)})\big]_j \;=\; \prod_{k=1}^{n} \big[\phi_k(\boldsymbol{u}_k)\big]_j \;=\; \psi_j(\boldsymbol{u}_1, \ldots, \boldsymbol{u}_n;\, \phi_1, \ldots, \phi_n),$$

where the first equality uses the substitution $k = \sigma(i)$ and the commutativity of $\mathbb{R}$. Since j was arbitrary, the equality holds componentwise, hence holds for the full vector ψ. □

Remarks. (i) The trunk network τ acts on a fixed coordinate input independent of the modality ordering, so the joint prediction $\hat{\mathbf{s}}(\mathbf{r} \mid \boldsymbol{u}) = \sum_j \psi_j(\boldsymbol{u})\, \tau_j(\mathbf{r}) + \boldsymbol{\beta}$ inherits the permutation invariance from ψ: the architecture as a whole is invariant under simultaneous reordering of the $(\boldsymbol{u}_i, \phi_i)$ pairs. (ii) The invariance is across *modalities* (heterogeneous input types) and is the property that makes the multi-input/multi-output architecture well-defined as the input list is reshuffled at the data-loading layer or at deployment when sensors are connected in different orders. It is distinct from invariance under permutations of trunk query coordinates, which is a property of the trunk's batched-evaluation pipeline rather than of the fusion. (iii) Permutation

invariance is shared by the additive aggregator $\psi_j = \sum_i [\phi_i(\boldsymbol{u}_i)]_j$ (by commutativity of + on $\mathbb{R}$). The multiplicative aggregator is chosen here because, in addition to invariance, it preserves the bilinear coupling structure that MIMONet uses to mirror the convective transport terms in the governing PDE. Whether this combination of properties uniquely selects the multiplicative aggregator among all permutation-invariant low-rank aggregators is not claimed here.

S3 Theoretical foundations

This section establishes what the operator formulation gives structurally, and marks the boundary past which the claims are empirical rather than proved. The distinction matters because the deployment properties the Introduction rests on are of the first kind: evaluation at arbitrary query coordinates and a fixed inference cost independent of the training-set size follow from the parameterisation itself, and do not have to be earned by measurement. Accuracy does not. Nothing below establishes that a learned operator reconstructs any particular field well; that is settled by the results in Sections S7 and S8.

The section proceeds from assumptions, to the variational reading of the training problem, to an approximation theorem, and then to four properties that bear on deployment. Except where stated, it concerns the linear branch–trunk contraction used by Problems 1–3; the scope of each statement for the measured-data problems is given in Section S5.1.

S3.1 Standing assumptions, and where they hold

The results in this section rest on three assumptions. They are stated here rather than invoked silently, and their scope is stated with them: they describe the simulation problems (Problems 1–3), whose samples are independent draws from a designed parameter distribution. Two of the three are known to fail on the measured-data problems, and we note where.

Assumption 1 (Operator Regularity)**.** *The true operator $\mathcal{G} : \mathcal{U} \to \mathcal{S}$ is Fréchet differentiable with bounded derivative $\|D\mathcal{G}\| \leq C_G$ for some constant $C_G > 0$.*

Assumption 2 (Data Distribution)**.** *Training samples $\{(\boldsymbol{u}^{(k)}, \mathbf{s}^{(k)})\}_{k=1}^{N}$ are drawn independently and identically distributed (i.i.d.) from a probability distribution μ supported on a compact subset $K \subset \mathcal{U}$.*

Assumption 3 (Latent Manifold Structure)**.** *The solution manifold $\mathcal{M} = \{\mathcal{G}(\boldsymbol{u}) : \boldsymbol{u} \in K\}$ is compact and is well approximated by a linear subspace of dimension at most I, in the sense that its Kolmogorov I-width is small relative to the target accuracy.*

These assumptions are physically motivated: Assumption 1 ensures well-posedness of the forward problem, Assumption 2 reflects the stochastic nature of operational conditions, and Assumption 3 formalises the observation that physical systems evolve on low-dimensional attractors despite living in infinite-dimensional function spaces. Two caveats belong here rather than later. Assumption 2 does not hold for Problems 4 and 6: their samples are consecutive recordings of an autocorrelated system split by a random permutation, so training and test draws are not independent, as Sections S4.4 and S4.6 state. Nothing in this section is used to derive an estimation rate, so no result here depends on it; it is retained because it is the condition under which the variational reading below is the natural one. Assumption 3 is empirically supported for Problems 1–3 and empirically *refuted* for Problem 6, where a fixed low-rank basis costs 12–18% relative accuracy against a nonlinear decoder (Section S8.3). That is a result about the problem rather than a defect of the theory, and it is why the measured-data models do not use the contraction readout. They are modelling assumptions on the underlying physics, and none of them is verified directly in this study; the quantities that are measured are stated as such throughout.

S3.2 Variational and functional-analytic interpretation

Let $\mathcal{U} \subset \prod_{i=1}^{n} \mathcal{F}_i$ and $\mathcal{S} \subset L^2(\mathcal{Y}; \mathbb{R}^m)$ denote separable Hilbert spaces of heterogeneous input modalities and output physical fields, respectively. The Hilbert structure of $\mathcal{S}$ is used below, in the nearest-point projection onto the learned subspace; since $\mathcal{S} \subset L^2(\mathcal{Y}; \mathbb{R}^m)$ throughout, this costs no generality here. Denote by $\langle \cdot, \cdot \rangle_{\mathcal{S}}$ the inner-product on $\mathcal{S}$ (or a corresponding dual pairing) and $\| \cdot \|_{\mathcal{S}}$ the induced norm.

We take the true physical forward mapping

$$\mathcal{G} : \mathcal{U} \;\to\; \mathcal{S}, \quad \boldsymbol{u} \mapsto \mathbf{s}$$

to be Lipschitz continuous on the domain of interest, that is, we assume there exists a constant $C > 0$ with

$$\|\mathcal{G}(\boldsymbol{u}_1) - \mathcal{G}(\boldsymbol{u}_2)\|_{\mathcal{S}} \;\le\; C\,\|\boldsymbol{u}_1 - \boldsymbol{u}_2\|_{\mathcal{U}} \quad \forall\,\boldsymbol{u}_1, \boldsymbol{u}_2 \in \mathcal{U}.$$

This is an assumption rather than a consequence of continuity: a continuous nonlinear operator need not be Lipschitz. It is implied by Assumption 1 above whenever the domain is open and convex, since a bounded Fréchet derivative gives the bound by the mean-value inequality.

We view the proposed surrogate operator (via the MIMONet architecture) as

$$\hat{\mathcal{G}} : \mathcal{U} \;\to\; \mathcal{S}, \qquad \hat{\mathcal{G}}(\boldsymbol{u})(\cdot) = \sum_{j=1}^{I} \psi_j(\boldsymbol{u})\; \tau_j(\cdot) \;+\; \boldsymbol{\beta},$$

where $\{\tau_j\}_{j=1}^{I} \subset \mathcal{S}$ are learned spatial basis functions (via the trunk network) and $\psi_j : \mathcal{U} \to \mathbb{R}$ are latent coefficient functions (via the branch network). $\boldsymbol{\beta} \in \mathbb{R}^m$ is a learned bias vector (channel-wise).

The map $\hat{\mathcal{G}}$ is nonlinear in $\boldsymbol{u}$, so it is not a finite-rank operator in the linear-operator sense; what is finite-dimensional is its *range*, which is contained in the affine subspace $\boldsymbol{\beta} + \operatorname{span}\{\tau_1, \ldots, \tau_I\} \subset \mathcal{S}$ of dimension at most I. The hypothesis class is

$$\mathcal{H}_I = \Big\{ \hat{\mathcal{G}} : \boldsymbol{u} \mapsto \sum_{j=1}^{I} \psi_j(\boldsymbol{u})\; \tau_j + \boldsymbol{\beta} \Big\}.$$

Writing $P_I : \mathcal{S} \to \boldsymbol{\beta} + \operatorname{span}\{\tau_1, \ldots, \tau_I\}$ for the orthogonal projection onto that affine subspace, one may decompose

$$\hat{\mathcal{G}}(\boldsymbol{u}) \;=\; P_I\, \mathcal{G}(\boldsymbol{u}) + \mathcal{E}_{\mathrm{opt}}(\boldsymbol{u}),$$

which *defines* the residual $\mathcal{E}_{\mathrm{opt}}$ collecting the discrepancy left by finite training. Setting $\epsilon_{\mathrm{train}} = \sup_{\boldsymbol{u}} \|\mathcal{E}_{\mathrm{opt}}(\boldsymbol{u})\|_{\mathcal{S}}$, the triangle inequality gives

$$\|\mathcal{G}(\boldsymbol{u}) - \hat{\mathcal{G}}(\boldsymbol{u})\|_{\mathcal{S}} \;\le\; \inf_{v \in \boldsymbol{\beta} + \operatorname{span}\{\tau_1, \ldots, \tau_I\}} \|\mathcal{G}(\boldsymbol{u}) - v\|_{\mathcal{S}} \;+\; \epsilon_{\mathrm{train}}.$$

The first term is the best approximation attainable within the learned subspace and the second is everything the optimiser fails to realise; the decomposition is definitional and is used below only to organise the discussion, not to supply a rate.

S3.3 The training objective as Tikhonov regularisation

It is instructive to read the training problem in the sense of Tikhonov regularisation, as

$$\min_{\hat{\mathcal{G}} \in \mathcal{H}_I} \frac{1}{N} \sum_{k=1}^{N} \|\hat{\mathcal{G}}(\boldsymbol{u}^{(k)}) - \mathbf{s}^{(k)}\|_{\mathcal{S}}^2 \;+\; \lambda_1\, \|\psi\|_{H^1}^2 + \lambda_2\, \|\tau\|_{H^1}^2,$$

where $\{(\boldsymbol{u}^{(k)}, \mathbf{s}^{(k)})\}_{k=1}^{N}$ are training pairs and the Sobolev penalties would enforce smoothness of the latent coefficient functions and of the spatial basis functions. We stress that this is an idealised reading and not the implemented objective: the models reported here are trained with an equally weighted mean-squared-error loss and the Adam optimiser with weight decay (Methods), which penalises parameter norm rather than a Sobolev norm of ψ or τ. Nor does such a penalty control the rank: the number of latent modes is fixed at I by the architecture, so the low-dimensionality of the representation is imposed by construction and is not an effect of regularisation.

Restricting the surrogate to I modes is nevertheless reasonable for the systems considered here, because the field variations they produce (convective and pressure-driven structures, boundary-layer shear modes, recirculation patterns) are strongly correlated across operating conditions and are well captured by a subspace of moderate dimension. The latent width I is a design hyperparameter, reported per case in the architecture tables, and the adequacy of a given choice is established empirically by the reconstruction errors rather than assumed.

S3.4 Universal approximation property

The multiplicative fusion restricts the form of the latent coefficients, so it is worth recording that it costs nothing in expressive power. The point requiring proof is that a *single* product $\prod_i [\phi_i(\boldsymbol{u}_i)]_j$ is separable across modalities and therefore cannot by itself represent a coefficient functional that couples them; expressivity is restored by the sum over the I latent components.

Theorem 2 (Universal Approximation for MIMO Operators)**.** *Let $\mathcal{G} : \mathcal{U} \to \mathcal{S}$ be a continuous operator, where $\mathcal{U} = \prod_{i=1}^{n} \mathcal{F}_i$ and $\mathcal{S} \subset L^2(\mathcal{Y}; \mathbb{R}^m)$. For any compact subset $K \subset \mathcal{U}$ and any $\epsilon > 0$, there exist a latent dimension $I \in \mathbb{N}$ and a MIMONet operator $\hat{\mathcal{G}}_I$ with multiplicative fusion such that*

$$\sup_{\boldsymbol{u} \in K} \|\mathcal{G}(\boldsymbol{u}) - \hat{\mathcal{G}}_I(\boldsymbol{u})\|_{\mathcal{S}} < \epsilon.$$

Proof sketch. **Step 1 (reduction to a rank-r expansion).** Since $\mathcal{G}$ is continuous and K compact, $\mathcal{G}(K) \subset \mathcal{S}$ is compact, hence has finite Kolmogorov width at every accuracy: there are $r \in \mathbb{N}$, functions $\tau_1, \ldots, \tau_r \in \mathcal{S}$ and continuous coefficient functionals $c_1, \ldots, c_r : K \to \mathbb{R}$ with $\sup_{\boldsymbol{u} \in K} \|\mathcal{G}(\boldsymbol{u}) - \sum_{j \le r} c_j(\boldsymbol{u}) \tau_j\|_{\mathcal{S}} < \epsilon/4$. This is the standard branch–trunk (DeepONet) reduction [2], and the c_j may be taken continuous because the projection onto a fixed finite-dimensional subspace is continuous.

Step 2 (products span the coefficient functionals). Consider

$$\mathcal{A} \;=\; \mathrm{span}\Big\{ \textstyle\prod_{i=1}^{n} f_i(\boldsymbol{u}_i) \;:\; f_i \in C(K_i) \Big\} \;\subset\; C(K),$$

the linear span of the modality-separable products. $\mathcal{A}$ is a subalgebra of $C(K)$: it is closed under multiplication, because a product of two such generators is again a generator; it contains the constants, by taking each $f_i \equiv 1$; and it separates points of K, since if $\boldsymbol{u} \neq \boldsymbol{u}'$ then $\boldsymbol{u}_i \neq \boldsymbol{u}'_i$ for some i and a single f_i separating them suffices. By the Stone–Weierstrass theorem $\mathcal{A}$ is dense in $C(K)$ in the uniform norm. Hence each c_j of Step 1 is approximated to within $\epsilon/(4r \max_j \|\tau_j\|_{\mathcal{S}})$ by a finite sum of m_j such products. It is this sum, not any individual product, that supplies the cross-modal coupling.

Step 3 (realisation by networks). Each factor f_i is a continuous functional on the compact set $K_i \subset \mathcal{F}_i$. For infinite-dimensional $\mathcal{F}_i$ we do not restrict to point evaluations, which are not bounded functionals on L^2; instead we use that a compact subset of a separable Hilbert space is approximated to any accuracy by a finite-dimensional projection, so there is d_i and a projection Π_{d_i} with $\sup_{\boldsymbol{u}_i \in K_i} \|\boldsymbol{u}_i - \Pi_{d_i} \boldsymbol{u}_i\| < \delta$. Uniform continuity of f_i on K_i then lets f_i be replaced, to within any prescribed accuracy, by a continuous function of the d_i projection coefficients, which a feedforward branch encoder approximates uniformly on a compact subset of $\mathbb{R}^{d_i}$. Since the factors are uniformly bounded on K, the factor-wise errors propagate through the product with a bounded multiplier, and the branch contribution is held to $\epsilon/4$. The trunk network likewise realises the τ_j to within $\epsilon/4$. In the discretised setting in which the branch input is already a finite vector of sensor readings, as it is in every case reported here, this step reduces to the classical branch–trunk approximation argument [3].

Collecting the four contributions, each at most $\epsilon/4$, and absorbing the $\sum_j m_j$ product terms into the latent index (replicating τ_j across the m_j slots that approximate c_j) gives a MIMONet of width $I = \sum_{j \le r} m_j$ meeting the bound by the triangle inequality. □

The construction is existential and gives no useful bound on I; the latent widths used in practice are set empirically and reported per case.

S3.5 Multiplicative fusion: empirical justification

Theorem 2 shows that multiplicative fusion does not restrict what can be represented, but it does not show that it is preferable to the alternatives. That question is settled by measurement rather than by argument, and we resolve it with an ablation on the heat-exchanger case in which the fusion rule is varied and everything else is held fixed. Table S4 reports the result.

Three observations follow. First, replacing the elementwise product by an elementwise sum raises the channel-mean error from 0.95% to 1.24%, a 30% relative degradation, at an identical parameter count and with every other component of the model, the data split and the optimisation schedule unchanged; the

Table S4: **Architectural ablation on the heat-exchanger case.** Channel-wise and channel-mean relative ℓ_2 error (%) on the held-out 310-sample test set. The additive variant differs from the baseline only in the fusion rule and has an *identical* parameter count, so the comparison between the first two rows isolates the fusion operation from model capacity.

**Variant**	**Params**	p	u_z	u_y	u_x	**Mean**
Multiplicative fusion (MIMONet)	1,762,052	0.82	1.45	1.02	**0.52**	**0.95**
Additive fusion	1,762,052	1.02	1.96	1.31	0.65	1.24
Concatenated input, single branch	1,104,900	0.81	1.65	1.02	0.54	1.00
Four per-channel models, joint decoding removed	6,258,692	**0.56**	**1.36**	1.20	0.91	1.01

fusion rule is therefore doing work that capacity cannot account for. Second, collapsing the modality-specific branches into a single branch acting on the concatenated input costs less accuracy (1.00%) but also removes the ability to accept modalities of different types and dimensions, which is the property the multi-input construction exists to provide. Third, training four independent single-channel models removes joint decoding and is instructive precisely because it is not uniformly worse: it is the most accurate variant on pressure (0.56% against 0.82%) and on u_z, yet it is worse on u_y and substantially worse on u_x (0.91% against 0.52%), and worse on the channel mean, while using 3.6× as many parameters. Shared-latent decoding thus trades a little per-channel specialisation for consistency across the coupled fields, and on the coupled channels that trade is favourable. The scale of these differences should be kept in proportion: the four variants span 0.95–1.24% channel-mean error, whereas the operator formulation as a whole differs from the classical estimators by factors of two to six on the problems of Section S8. This ablation justifies a design choice within the architecture; it is not the comparison on which the paper's argument rests.

S3.6 Error decomposition and measured convergence

The error of the trained operator separates into three contributions of different origin: an *approximation* term, the distance from $\mathcal{G}$ to the hypothesis class $\mathcal{H}_I$, governed by how well an I-dimensional subspace captures the solution manifold; an *estimation* term, reflecting that the class is fitted on N samples rather than on the population; and an *optimisation* term, reflecting that a non-convex objective is minimised approximately. We do not state a rate for the estimation term. The generalisation bounds available for classes of this kind are asymptotic in character and hold in expectation or with high probability over the draw of the training set, not pointwise in $\boldsymbol{u}$, and the constants they carry are far too loose to predict the behaviour observed at the sample sizes used here.

What can be reported instead is the measured convergence. Sweeping the training-set size and fitting $\epsilon(N) = a\,N^{-\gamma}$ to the held-out error gives $\gamma = 0.66 \pm 0.12$ for the PWR subchannel, $\gamma = 0.43 \pm 0.11$ for the heat exchanger and $\gamma = 0.58 \pm 0.23$ for the lid-driven cavity (Section S9.3), with the intervals being 95% confidence intervals on the exponent from a five-point fit. These exponents are consistent with the Monte-Carlo-like rates expected when the estimation term dominates, but the fits rest on five points each and are reported as measurements, not as verifications of a bound.

S3.7 Mesh independence and continuous extension

This subsection addresses the first of the deployment requirements set out in the Introduction: that the reconstructed field be evaluable at arbitrary coordinates, rather than at a grid or a set of locations fixed when the estimator was built.

Because the trunk network $\tau : \mathbb{R}^d \to \mathbb{R}^{I\times m}$ takes the query coordinate $\mathbf{r}$ as a continuous input, the learned operator admits evaluation at any coordinate $\mathbf{r} \in \mathcal{Y}$ without retraining or regridding: a new query is simply a new input to τ, and the corresponding decoding weights are produced by a single forward pass. This is a structural property of the branch–trunk formulation, and it distinguishes the operator from fixed-grid surrogates (e.g. convolutional decoders) whose output discretisation is committed at training time. We emphasise that this is a statement about the *admissibility* of arbitrary query coordinates, not a guarantee of uniform accuracy: reconstruction fidelity at a given coordinate depends on the local density of training

nodes and on the spatial regularity of the target field, and is therefore an empirical rather than an assumed property.

S3.8 Comparison with kernel methods and Gaussian processes

MIMONet can be viewed as learning a data-dependent kernel $K(\mathbf{r},\mathbf{r}') = \sum_{j=1}^{I} \tau_j(\mathbf{r})\tau_j(\mathbf{r}')$, generalising to tensor-valued kernels for multi-output learning:

$$K^{(o,o')}(\mathbf{r},\mathbf{r}') = \sum_{j=1}^{I} \tau_j^{(o)}(\mathbf{r})\tau_j^{(o')}(\mathbf{r}').$$

The practical difference is not one of asymptotic order in the number of query points: exact Gaussian-process regression conditioned on n observations costs $\mathcal{O}(n^3)$ to factorise the covariance once and then $\mathcal{O}(Pn)$ for predictive means at P query points, which is linear in P, as is the $\mathcal{O}(PIm)$ branch–trunk contraction (Section S10). The difference is where the cubic term sits. For a Gaussian process the $\mathcal{O}(n^3)$ conditioning is incurred *per input configuration*: a new set of sensor readings is a new regression problem and the solve must be repeated. The operator pays its training cost once, offline, and thereafter answers a new input with a single branch forward pass followed by the contraction, with no linear system to solve at deployment. It is this amortisation, rather than the scaling in P, that makes operator inference viable inside a monitoring loop, and it is the same property that produces the sub-millisecond latencies reported in the main text.

S3.9 Low-rank structure of the learned operator

The finite-rank ansatz has a population-level optimality that is worth stating precisely, because it explains what the latent basis is for.

Let $\bar{\mathcal{G}} = \mathbb{E}_{\boldsymbol{u}\sim\mu}[\mathcal{G}(\boldsymbol{u})]$ and let

$$C_{\mathcal{G}} = \mathbb{E}\big[(\mathcal{G}(\boldsymbol{u}) - \bar{\mathcal{G}}) \otimes (\mathcal{G}(\boldsymbol{u}) - \bar{\mathcal{G}})\big]$$

be the covariance operator of the output fields. Since $\mathcal{M}$ is bounded, $C_{\mathcal{G}}$ is trace class, self-adjoint and positive semi-definite, hence admits an eigendecomposition $C_{\mathcal{G}} = \sum_{j\geq 1} \lambda_j\, \varphi_j \otimes \varphi_j$ with $\lambda_1 \geq \lambda_2 \geq \cdots \geq 0$ and orthonormal $\{\varphi_j\}$. Among all approximations of the form $\boldsymbol{u} \mapsto \boldsymbol{\beta} + \sum_{j\leq I} c_j(\boldsymbol{u})\, \tau_j$ with arbitrary coefficient functions c_j, the mean-squared error $\mathbb{E}\|\mathcal{G}(\boldsymbol{u}) - \cdot\|_{\mathcal{S}}^2$ is minimised by taking $\boldsymbol{\beta} = \bar{\mathcal{G}}$, $\operatorname{span}\{\tau_j\} = \operatorname{span}\{\varphi_1, \ldots, \varphi_I\}$ and c_j the corresponding orthogonal-projection coefficients: for a fixed subspace the optimal coefficients are those of the orthogonal projection, and optimising over I-dimensional subspaces returns the leading eigenspace of $C_{\mathcal{G}}$. This is the Karhunen–Loève truncation, the function-space counterpart of a truncated singular value decomposition, and its error is $\sum_{j>I} \lambda_j$; the optimal subspace is unique when $\lambda_I > \lambda_{I+1}$.

It does not follow that $\mathcal{H}_I$ contains this optimum at width I, and we do not claim that it does. Two obstructions stand in the way. The offset $\boldsymbol{\beta}$ is a channel-wise constant in $\mathbb{R}^m$ rather than a field, so a spatially varying mean $\bar{\mathcal{G}}$ has to be carried by one of the I latent modes, leaving a mean-plus-rank-$(I-1)$ budget. More substantively, the Karhunen–Loève optimum requires the coefficient functionals to be exactly $c_j(\boldsymbol{u}) = \langle \mathcal{G}(\boldsymbol{u}) - \bar{\mathcal{G}}, \varphi_j \rangle$, which couple the modalities, whereas multiplicative fusion forces each ψ_j to be a modality-separable product; this is the same restriction noted before Theorem 2, and Theorem 2 circumvents it only by enlarging the width to $\sum_j m_j$. The correct reading is therefore that the Karhunen–Loève truncation is the benchmark an ideal rank-I approximation would attain, that MIMONet approaches it at sufficient width, and that nothing here bounds the width required. A third limitation is separate from both: this is a statement about the population optimum, whereas training minimises an empirical risk over a non-convex parameterisation with no guarantee of reaching any optimum. Finally, the individual τ_j are not identifiable: for any invertible A, replacing $\psi \mapsto A^\top \psi$ and $\tau \mapsto A^{-1}\tau$ leaves $\hat{\mathcal{G}}$ unchanged, and the multiplicative fusion still admits the diagonal and permutation elements of that freedom. The learned basis should therefore be read as spanning an informative subspace, not as recovering the eigenfunctions φ_j individually, and we make no claim that it does.

S4 Dataset preparation

Six datasets are described below: three generated with a CFD solver and three measured. They are reported at a level of detail that lets a reader judge what each reported error means, which for virtual sensing turns almost entirely on two things: what the operator is given at inference, and how the split was drawn. Both are stated plainly for every problem, including where the protocol is weaker than a reader might assume. The split for Problems 4 and 6 is a random permutation over temporally adjacent samples, not a chronological one, and we say so in each case rather than leaving it to be inferred from the code.

S4.1 Transient lid-driven cavity (Problem 1)

The transient flow dataset was generated from two-dimensional simulations of a square lid-driven cavity of side length 0.065 m. The flow was governed by the incompressible Reynolds-averaged Navier–Stokes (RANS) equations,

$$\frac{\partial \mathbf{u}}{\partial t} + (\mathbf{u} \cdot \nabla)\mathbf{u} = -\nabla p + \nabla \cdot \left[(\nu + \nu_t)(\nabla \mathbf{u} + \nabla \mathbf{u}^{\mathsf{T}})\right], \tag{2}$$

subject to the incompressibility constraint $\nabla \cdot \mathbf{u} = 0$, where ν and ν_t denote the molecular and eddy viscosities, respectively. The top wall (lid) translated in the x-direction, while the remaining walls were stationary and enforced with no-slip conditions.

To introduce temporal variability and generate diverse transient flow patterns, the lid velocity was prescribed as a time-dependent function $u_{\text{lid}}(t)$, drawn from a set of smoothly varying trajectories. Each trajectory was constructed from random coefficients ensuring gradual accelerations and decelerations within the velocity range 0.1–0.3 m/s. The time-varying lid forcing induced unsteady vortical structures within the cavity, providing rich spatio-temporal variability suitable for data-driven modelling.

A total of 4,937 high-fidelity transient simulations were performed, each corresponding to a unique lid-velocity history. The simulations were integrated until statistically steady behaviour was achieved, and the complete time-evolving velocity field $\mathbf{u}(x, y, t)$ and pressure field $p(x, y, t)$ were recorded. For each case, the stream function $\psi(x, y, t)$ was computed using

$$u = \frac{\partial \psi}{\partial y}, \qquad v = -\frac{\partial \psi}{\partial x}, \tag{3}$$

with $\psi = 0$ applied on all boundaries. The streamfunction formulation defines the flow, but the fields exported for operator training are pressure, velocity magnitude and turbulent kinetic energy $\{p, \|\boldsymbol{v}\|, k\}$ on the 4,225-node mesh, as listed in Table S2; the symbol ψ is used here only for the streamfunction of the generating simulation and is unrelated to the fused branch latent ψ_j of Section S2. The data generation process produced a diverse ensemble of transient flow evolutions, encompassing varying vortex intensities and shedding frequencies, providing sufficient variability for robust learning of time-dependent flow operators.

S4.2 PWR subchannel (Problem 2)

A second series of simulations was carried out under high-temperature, high-pressure operating conditions representative of pressurised water reactor (PWR) environments. The computational domain consisted of a single subchannel of a hexagonal fuel rod bundle, discretised to capture the detailed thermo-hydraulic behaviour of superheated water flow. A total of 5,000 stochastic cases were generated by varying inlet velocity, temperature, and heat generation parameters to imitate realistic reactor operating variability.

The simulated domain represented a triangular subchannel formed by three adjacent fuel rods within a hexagonal lattice. Each fuel rod had a diameter of 9.1 mm, and the rod-to-rod pitch was 12.75 mm, resulting in a pitch-to-diameter ratio of $P/D = 1.4$. The hydraulic diameter of the subchannel was 10.64 mm, while the total axial length was $L = 605$ mm. The geometric dimensions were adopted to ensure fully developed turbulent flow and to replicate realistic subchannel-scale heat transfer characteristics. A polyhedral mesh with prismatic inflation layers was generated to accurately resolve near-wall gradients. The mesh density ranged from 0.4 to 2.1 million cells, with the final simulations performed on the refined grid. The near-wall treatment satisfied $y^+ \leq 1$, ensuring that the viscous sublayer was adequately captured. Mesh independence was verified by monitoring the variation in pressure drop and Nusselt number across successive

refinements, with less than 2% deviation observed. The fluid properties corresponded to pressurised water at $p_{\text{ref}} = 15.5$ MPa and $T_{\text{ref}} = 565$ K. The inlet velocity was defined within the range 2–7 m/s, producing turbulent Reynolds numbers in the order of 10^5. The heated rod surfaces were subjected to a sinusoidal heat flux of $q_{\max} = 6.0 \times 10^5$ W/m^2, while the remaining walls were treated as adiabatic and no-slip. The outlet pressure was fixed at 15.7 MPa. These conditions were selected to replicate nominal subchannel flow under steady-state forced convection. To capture realistic operating variability, random perturbations of ±10% were introduced to the inlet velocity, temperature, and heat generation parameters:

$$T_{\text{in}} = T_{\text{ref}} + \delta_T, \qquad v_{\text{in}} = v_{\text{ref}} + \delta_v, \qquad q_{\max} = q_{\text{ref}}(1 + \delta_q),$$

where δ_T, δ_v, and δ_q were sampled from uniform distributions within their respective limits. The axial heat generation profile in each rod was modelled using a sinusoidal distribution,

$$q''(z) = q_{\max} \sin\left(\frac{\pi z}{L}\right),$$

representing peaking near the midplane and attenuation toward the rod ends. Gaussian-correlated perturbations were further applied to the inlet temperature field to mimic coherent thermal fluctuations. All simulations were performed in ANSYS Fluent using the steady-state Reynolds-Averaged Navier–Stokes (RANS) equations with the RNG k–ε turbulence model and enhanced wall treatment. The SIMPLEC algorithm was employed for pressure–velocity coupling, and second-order upwind discretisation was used for all governing equations. Convergence was ensured when the residuals for momentum fell below 10^{-6} and for energy below 10^{-8}. Each simulation produced spatial distributions of temperature, velocity, pressure, and wall heat flux, along with derived quantities such as local Nusselt number, friction factor, and wall temperature profiles. The ensemble of 5,000 stochastic simulations forms a comprehensive dataset representing the thermo-hydraulic behaviour of hexagonal rod-bundle subchannels under variable inlet and heat generation conditions.

S4.3 Heat exchanger (Problem 3)

To ensure statistical diversity and robustness of the simulation database, a total of 1,546 independent CFD cases were generated by randomly perturbing the inlet velocity, wall heat flux, and inlet temperature within defined physical limits. Each simulation employed the optimised geometric configuration of the dimpled channel with wavy tape inserts, characterised by $W/D = 0.66$, $A/W = 0.27$, and $t/D = 0.0417$, as determined from the parametric analysis. The simulations were conducted under constant wall heat flux and steady turbulent flow conditions. The governing heat flux at the heated wall was specified as

$$q'' = q_{\max} \sin\left(\frac{\pi z}{H}\right),$$

where $q_{\max} = 20$ kW/m^2 and $H = 0.8$ m represent the maximum heat generation intensity and the channel height, respectively. For each case, $q_{\max}$ was randomly perturbed by up to ±10% to initiate spatial and operational variability in heat generation. The remaining walls were treated as adiabatic and enforced with no-slip velocity conditions.

The inlet temperature of the working fluid was defined as

$$T_{\text{in}} = T_{\text{ref}} + \delta_T,$$

where $T_{\text{ref}} = 293$ K and δ_T was drawn from a uniform distribution within ±10% of T_{ref}. To represent spatially correlated fluctuations, an auxiliary Gaussian random field was applied to generate smooth spatial variations of temperature along the inlet boundary.

The inlet velocity was varied similarly:

$$v_{\text{in}} = v_{\text{ref}} + \delta_v, \qquad v_{\text{ref}} = 4.5 \text{ m/s},$$

where δ_v followed a uniform perturbation of ±10%. This resulted in Reynolds numbers spanning approximately 1.0×10^4 to 2.5×10^4. The outlet boundary was fixed at zero gauge pressure, ensuring fully developed flow.

Each case was solved under steady-state conditions using the ANSYS Fluent solver with the RNG k–ε turbulence model and enhanced wall treatment. The SIMPLEC algorithm was employed for pressure–velocity coupling, and a second-order upwind discretisation was used for momentum and energy equations. Convergence was accepted at residual thresholds of 10^{-6} for momentum and 10^{-8} for energy. For every simulation, spatial fields of velocity, temperature, and pressure were exported along with derived quantities such as Nusselt number, friction factor, and performance evaluation criterion (PEC). The resulting 1,546 samples constitute a comprehensive stochastic dataset capturing the thermal–hydraulic response of the optimised wavy-taped dimpled channel under realistic perturbations of inlet and thermal boundary conditions.

S4.4 Proton-exchange-membrane fuel cell (Problem 4)

Source and channels. The measurements are segmented-cell recordings from a laboratory PEM fuel cell, comprising a current-density map and a temperature map recorded in parallel over eight experiment directories (four gas configurations × two load profiles). Current density is parsed on an 18×18 grid, giving the 324 segments referred to throughout, and its magnitude is retained. Temperature is parsed on a 9×9 grid.

The temperature channel is interpolated, not measured at 324 points. The two channels are recorded on different grids, and the pipeline reconciles them by bilinearly upsampling the 9×9 temperature map onto the 18×18 grid before the two are stacked. The temperature channel therefore carries only 81 independent measurements spread over 324 nodes, and no code path retains a native 9×9 temperature field. This has a direct consequence for how the reported errors should be read: the temperature target is itself a smooth bilinear interpolant, so it is intrinsically easier to reconstruct than the current-density field, and the very small temperature errors reported in Section S8.1 (of order 0.03–0.05%) reflect that smoothness as much as they reflect operator accuracy. The current-density channel, which is measured at all 324 segments, is the informative one, and it is the channel on which the comparison with the classical interpolants is decided.

Temporal subsampling and frame budget. Every third recorded block is retained, the eight experiments are concatenated, and provenance is not preserved in the stored array. If the concatenated record exceeds 8,000 frames a random subset of 8,000 is drawn without replacement (seeded) and sorted; the shipped dataset has exactly $T = 8{,}000$ frames, so this branch was taken. The number of raw blocks before subsampling is not recorded, and neither the sampling interval in seconds nor the cell voltage is parsed, so the frames carry no physical time axis.

Sensor selection. For a given seed, a permutation of the 324 grid indices is drawn; the first n_{obs} indices are the observed sensors and the remainder are held out. Both branches read the *same* observed set, one supplying current density and the other temperature at those locations. The coverage levels swept are $n_{\text{obs}} \in \{6, 16, 32, 81, 162\}$, i.e. approximately 2%, 5%, 10%, 25% and 50% of the 324 segments; the operating point quoted in the main text is $n_{\text{obs}} = 32$, which is 9.9% rather than exactly 10%. The same seed reproduces the same sensor set for every model, so MIMONet, KCN and the classical interpolants are compared on identical sensor layouts.

Split, and the fact that it is not chronological. The 8,000 frames are split by a uniform random permutation of frame index into 5,600 training, 1,200 validation and 1,200 test frames. This is a random split, not a chronological one. Because the frames are consecutive recordings subsampled by a factor of three, a test frame can be temporally adjacent to a training frame, and no blocking or minimum-separation rule is applied. The reported errors therefore characterise reconstruction from sparse sensors on operating states that are close to states seen in training, and they should not be read as evidence of extrapolation to new operating regimes. Because experiment provenance is discarded at concatenation, it is also not possible to verify from the stored data whether any test frame comes from a gas configuration absent from training; no such generalisation is claimed.

Coordinates and normalisation. Grid coordinates are integer indices, min–max mapped to $[-1, 1]$ per axis; no physical cell pitch enters the pipeline, so the trunk coordinates and the distances used by the classical interpolants are in grid-index units. Targets are standardised per channel using means and standard deviations computed on training frames only, and predictions are returned to physical units before scoring. Errors are relative ℓ_2 per channel, evaluated on the held-out nodes only and averaged over test frames.

S4.5 IEA-Wind tall towers (Problem 5)

Source and towers. Seven towers are taken from the BSC/IEA-Wind Tall Tower Dataset [1]: Boseong, Lindenberg, NWTC M2, NWTC M4, NWTC M5, Hegyhatsal and Wallaby Creek, selected as the towers carrying a near-surface level together with measurements reaching to about 80 m or above. Each tower contributes hourly means obtained by resampling its native cadence, which is ten-minutely at five towers, hourly at Hegyhatsal and half-hourly at Wallaby Creek. After the retention rule below, the pooled dataset contains 601,454 profiles.

The canonical heights are interpolated onto a common grid. Every tower measures at its own set of levels, so both the input heights $(10, 25, 50)$ m and the query heights $(60, 70, 80, 100)$ m are obtained by interpolation that is linear in $\log_{10} z$ across each tower's measured profile, with no extrapolation: a canonical height outside a tower's measured range returns a missing value. Two consequences should be stated plainly. First, *no tower measures at* 25 *m*, so the middle input channel is an interpolated quantity at every tower rather than a sensor reading. The hub height of 80 m is a genuine measured level at five of the seven towers and is interpolated at Hegyhatsal and Wallaby Creek. Second, 100 m lies above the topmost level at Lindenberg (98 m) and NWTC M2 (80 m), so those two towers contribute no 100 m target at all; this is why the test count falls from 90,222 profiles at 60–80 m to 34,967 at 100 m, and it means the 100 m column is evaluated on a different and smaller subset of towers than the other three.

Quality control. A profile is treated as missing unless at least two finite measured levels exist at that timestamp, and a timestamp is retained only if all three input heights and the 80 m target are finite. No threshold, spike or stationarity filter is applied, and the quality flags carried by the source files are not read by the pipeline; the dataset is used as distributed. Hourly means are formed from whatever valid sub-hourly samples exist, with no minimum-coverage requirement.

Split. Each tower is split chronologically in its own time order into 70% training, 15% validation and 15% test, giving 421,013, 90,219 and 90,222 profiles pooled across towers. The held-out dimension is time. Note that the network is trained with supervision at all seven canonical heights wherever a target is finite, so the 60–100 m targets are seen during training on training-period timestamps; what the model never receives at any time, in training or at inference, is an *input* sensor above 50 m. The claim being tested is therefore reconstruction above the instrumented range of the input sensors, not discovery of a height regime absent from the training signal.

Contextual inputs and normalisation. The second branch receives a four-dimensional context vector (lat/90, lon/180, $\sin(2\pi h/24)$, $\cos(2\pi h/24)$), where h is the hour of day; tower identity enters only through latitude and longitude, and there is no tower embedding and no per-tower model. Input sensor values are z-scored per channel on training rows. Targets are standardised by a *single global* mean and standard deviation taken over all heights and all training rows, not per height. The query height enters the trunk as a standardised $\log_{10} z$ coordinate. Errors are relative ℓ_2 in physical units at each evaluation height.

S4.6 GLORYS12 North Atlantic reanalysis (Problem 6)

Source, domain and grid. Surface fields of potential temperature (`thetao`), salinity (`so`) and sea-surface height (`zos`) are taken from the GLORYS12V1 global ocean reanalysis over 35–50°N, 60–20°W, for 730 daily snapshots spanning 2017-01-01 to 2018-12-31. Only the surface level is used. The native grid is block-averaged by a factor of three in each direction, and a cell is retained only if it is finite in all three variables at every

time step, leaving $P = 9{,}457$ ocean cells. Land is excluded by this mask rather than by a separate coastline product.

Sensor networks. A single permutation of the 9,457 ocean-cell indices is drawn per seed; the first 400 indices form the temperature network and the next 400 the salinity network. Disjointness is therefore enforced by construction, and the two sets never intersect. Sensor positions are fixed across all days. Sea-surface height is supplied to no branch: each branch input stacks only $[\text{value}, \text{lat}, \text{lon}]$ from its own channel, so the SSH field is a decoder output driven entirely by the fused temperature and salinity latents. SSH does enter the per-channel target normalisation constants and the land mask, but never the input.

Split, and the fact that it is not chronological. The 730 days are split by a random permutation into 610 training, 60 validation and 60 test days. This is a random split over days, not a forward-in-time split; test days are interleaved with training days. Because daily ocean fields are strongly autocorrelated, this places essentially every test day next to a training day, and the resulting figures measure sparse-sensor reconstruction of states that are close to states seen in training. Section S8.4 quantifies what this protocol does and does not establish, and reports a forward-in-time split as a robustness check.

Normalisation. Targets are standardised per channel using statistics computed on training days only and pooled over all cells. Branch sensor values are standardised by a single scalar mean and standard deviation per channel, again on training days. Coordinates are min–max mapped to $[-1, 1]$ over the ocean cells. Predictions are returned to physical units before scoring, and errors are relative ℓ_2 per channel over the test days. The classical baselines operate on physical values; Geo-FNO operates on standardised values.

S5 Architectures and training configurations for all six problems

The per-problem architectures are collected in Table S5 and the corresponding optimisation settings in Table S6. Both are reported in full because the comparisons in this work are between methods rather than between tunings, and a reader checking that a baseline was given a fair budget needs the widths and the schedules, not a summary of them.

S5.1 Two readout variants are used, and which one applies where

The six problems do not all use the same readout, and the distinction matters for reading the rest of this document.

The three simulation problems use the *linear* branch–trunk contraction described in the main text. The trunk emits $I \times m$ values per query coordinate, the fused branch latent is contracted against them, and a learned per-channel bias is added:

$$\hat{\mathbf{s}}(\mathbf{r}) \;=\; \sum_{j=1}^{I} \psi_j(\boldsymbol{u})\, \tau_j^{(o)}(\mathbf{r}) \;+\; \beta_o, \qquad \psi_j(\boldsymbol{u}) = \prod_i \big[\phi_i(\boldsymbol{u}_i)\big]_j .$$

This is the form assumed throughout Sections S3.2 and S3: the range of the operator lies in a finite-dimensional affine subspace, which is what makes the finite-rank and Karhunen–Loève discussion applicable.

The three measured-data problems use a *nonlinear* decoder. This variant is referred to as MIMONet-NL ("NL" for nonlinear readout) wherever the two have to be distinguished in a table or figure; elsewhere "MIMONet" without qualification refers to whichever readout the problem under discussion uses, as listed in Table S5. The branches are still fused multiplicatively, but the fused latent is then concatenated with the trunk feature and passed through a multilayer perceptron:

$$\hat{\mathbf{s}}(\mathbf{r}) \;=\; \mathrm{MLP}\Big(\big[\,\tau(\mathbf{r})\,;\,\textstyle\prod_i \phi_i(\boldsymbol{u}_i)\,\big]\Big).$$

There is no contraction and no per-channel trunk basis; the output channels are produced by the decoder's final linear layer. The consequence for the theory is explicit: the output of this variant does *not* lie in

a fixed finite-dimensional subspace, so the low-rank statements of Section S3 characterise the simulation models and not the measured-data models. The same scoping applies to the approximation theorem of that section, whose proof constructs a finite sum $\sum_{j\leq I} c_j(\boldsymbol{u})\,\tau_j$ and therefore concerns the contraction readout: it is not a guarantee for the MLP-decoder variant used in Problems 4–6. Universality for that variant follows instead from the standard argument for branch–trunk architectures with a nonlinear decoder [2, 4], which we cite rather than reprove. Assumption 3 should likewise be read as an assumption about the simulation problems: the ocean comparison in Section S8.3 is direct evidence that a fixed low-rank basis is *not* adequate for Problem 6, which is a result about that problem rather than a defect of the theory. The permutation invariance of the multiplicative fusion (Supplementary Note 1) is unaffected and holds for both variants, since it is a property of the fusion rather than of the readout.

This design was adopted for the measured-data problems because their targets are not smooth solution fields of a single governing system evaluated on a fixed mesh, and a fixed low-rank basis proved too restrictive; the ocean comparison in Section S8.3, where the nonlinear decoder improves on the linear readout by 12–18% relative at comparable parameter count, is the direct measurement of that.

S5.2 Architectures

Table S5: **MIMONet configurations for all six problems.** Layer widths are listed as input followed by hidden and output widths. "Contraction" denotes the linear branch–trunk readout, "MLP decoder" the concatenate-and-decode variant of Section S5.1. Fourier entries give the number of random frequencies and the sampling scale of the trunk feature map; "—" means the raw coordinate is used.

Problem	Branch 1	Branch 2	Trunk	Fourier	I	Readout	Params
1. Lid-driven cavity	$[90, 512, 512, 512, 256]$	—	$[2, 256, 256, 256, 256{\times}3]$	—	256	Contraction	1,032,963
2. PWR subchannel	$[100, 512, 512, 512, 256]$	$[2, 512, 512, 512, 256]$	$[2, 256, 256, 256, 256{\times}3]$	—	256	Contraction	1,696,259
3. Heat exchanger	$[100, 512, 512, 512, 256]$	$[2, 512, 512, 512, 256]$	$[2, 256, 256, 256, 256{\times}4]$	—	256	Contraction	1,762,052
4. PEM fuel cell	$[3n_{\mathrm{obs}}, 256^{\times 4}]$	$[3n_{\mathrm{obs}}, 256^{\times 4}]$	$[128, 256, 256, 256]$	64, σ=3	256	MLP decoder	872,450
5. IEA-Wind towers	$[3, 128^{\times 4}]$	$[4, 128^{\times 4}]$	$[64, 128, 128, 128]$	32, σ=3	128	MLP decoder	207,617
6. GLORYS12 ocean	$[1200, 384^{\times 4}]$	$[1200, 384^{\times 4}]$	$[256, 384, 384, 384]$	128, σ=3	384	MLP decoder	2,795,907

For the measured-data problems the decoder widths are $[2I, I, I, I, m]$, i.e. four linear layers taking the concatenated trunk-and-latent vector of width $2I$ to the m output channels, with GELU between layers. Problem 4 has $m = 2$ (J, T), Problem 5 has $m = 1$ (wind speed, evaluated at each query height), Problem 6 has $m = 3$ (T, S, SSH). The branch input dimension for Problem 4 is $3n_{\mathrm{obs}}$ because each observed segment contributes its value and its two normalised coordinates; at the $n_{\mathrm{obs}} = 32$ operating point this is 96, and the parameter count grows by 1,536 for each additional sensor. For Problem 6 it is $1{,}200 = 400 \times 3$ per branch. The trunk of Problem 5 takes the query *height* alone, so its Fourier map acts on a one-dimensional coordinate; Problem 4 draws its basis over a three-column coordinate whose third entry is constant, so the encoding is effectively two-dimensional, and Problem 6 uses a two-dimensional latitude–longitude basis at the surface level. In all three the Fourier matrix is drawn from $\mathcal{N}(0, \sigma^2)$ with a generator seeded at $\mathrm{seed} + 7$, so the trunk basis itself changes with the run seed.

S5.3 Training configurations

No problem uses early stopping in the usual sense. On every case the full epoch budget is consumed and the best-validation checkpoint is restored at the end; the patience of 10 reported in the Schedule column belongs to `ReduceLROnPlateau` and not to a stopping rule. Problem 6 subsamples 3,000 of the 9,457 query cells per optimisation step, drawn uniformly with replacement and shared across the batch; Problems 4 and 5 use all query coordinates in every step. The loss is a mean-squared error on standardised targets in every case, masked to the finite entries where a target can be missing (Problems 4 and 5).

Seeds and repeated runs. The measured-data problems are each run at five seeds, 0 through 4, and the tables in Section S8 report the mean and standard deviation across those runs. A seed sets NumPy and PyTorch generators and, through them, the weight initialisation, the batch shuffling, the Fourier basis (via

Table S6: **Training hyperparameters for all six problems.** "Best-val" denotes that the full epoch budget is always run and the parameters restored from the epoch with the lowest validation loss; where a validation interval is given, the validation loss is evaluated only every that many epochs plus the final epoch. Batch sizes are in samples for Problems 1–4 and 6 and in profiles for Problem 5; every batch evaluates all query coordinates unless a subsample is stated.

Problem	Optimiser	LR	Weight decay	Schedule	Epochs	Batch	Stopping
1. Lid-driven cavity	Adam	10^{-3}	10^{-7}	$\times 0.5$ on plateau	≤ 500	8	Best-val checkpoint
2. PWR subchannel	Adam	10^{-3}	10^{-6}	$\times 0.5$ on plateau	≤ 500	4	Best-val checkpoint
3. Heat exchanger	Adam	10^{-3}	10^{-6}	$\times 0.5$ on plateau	≤ 500	4	Best-val checkpoint
4. PEM fuel cell	Adam	10^{-3}	10^{-5}	Cosine to 10^{-6}	200	64	Best-val, interval 3
5. IEA-Wind towers	Adam	10^{-3}	10^{-5}	Cosine to 10^{-6}	60	4,096	Best-val, interval 3
6. GLORYS12 ocean	Adam	10^{-3}	10^{-5}	Cosine to 10^{-6}	300	16	Best-val, interval 5

seed + 7) and the sensor draw; for Problems 4 and 6 it also sets the data split, so the five runs differ in split as well as in initialisation. Problem 5 has a fixed chronological split, so its five runs differ only in initialisation, shuffling and Fourier basis, which is why its seed-to-seed spread is the smallest of the three. Bit-exact reproducibility on GPU is not enforced: none of the scripts calls `torch.use_deterministic_algorithms` or fixes the cuDNN backend, and Problem 4 does not seed `torch.cuda` explicitly, so re-running may shift the last reported digit.

S5.4 Normalisation, coordinate scaling and Fourier features

Every statistic used for normalisation is computed on training data alone and then applied unchanged to the validation and test partitions; no test sample contributes to any mean, standard deviation or extremum used by the pipeline. The schemes differ across problems because the quantities differ, and the differences are consequential enough to state in one place.

Inputs. The simulation problems (1–3) standardise the functional and scalar branch inputs with training-set means and standard deviations. Problem 4 standardises each measured channel with means and standard deviations taken over training frames only. Problem 5 standardises wind speed with a *single global* mean and standard deviation pooled over all heights and all training rows rather than per height, so that the model is not handed the height-conditional statistics it is asked to infer. Problem 6 standardises each of T, S and SSH per channel with statistics computed on training days and pooled over all cells.

Outputs. The simulation problems map each output channel independently to $[-1, 1]$ using training-set extrema. The measured-data problems predict in the standardised space of the corresponding input channel and invert the transform before any error is computed, so all reported errors are in physical units.

Query coordinates. Trunk coordinates are scaled to $[-1, 1]$ per axis. No physical cell pitch enters the pipeline for Problems 4 and 6, so trunk coordinates and the distances used by the classical interpolants are in scaled units, and for Problem 6 the interpolant distances are scaled latitude–longitude rather than great-circle distance. Problem 5 uses a standardised $\log_{10} z$ height coordinate, which makes the trunk input linear in the variable that classical shear profiles are linear in.

Fourier features. The measured-data problems encode the trunk coordinate with random Fourier features before the trunk MLP. A basis $B \in \mathbb{R}^{M \times d}$ is drawn once with $B_{ij} \sim \mathcal{N}(0, \sigma^2)$ and held fixed for the run, and the coordinate $\mathbf{r}$ is mapped to

$$\gamma(\mathbf{r}) \;=\; \big[\sin(2\pi B\mathbf{r});\, \cos(2\pi B\mathbf{r})\big] \in \mathbb{R}^{2M},$$

so the trunk input width is $2M$. The pairs (M, σ) are the "Fourier" column of Table S5: $(64, 3)$ for Problem 4, $(32, 3)$ for Problem 5 and $(128, 3)$ for Problem 6. The basis is drawn from the run's seed, so it varies across

the five seeds along with the sensor draw and the initialisation, and its contribution is therefore included in the reported seed spread rather than held fixed. The encoding is used because a plain coordinate MLP is slow to represent the high-frequency spatial structure these targets carry; the simulation problems, whose targets are smooth fields on a fixed mesh, do not use it.

S5.5 Baseline definitions

The classical baselines are stated here exactly as implemented, because several of them carry names that are used loosely in the literature.

Inverse-distance weighting (IDW). Weights $w_k \propto \|\mathbf{r} - \mathbf{r}_k\|^{-2}$ over *all* observed sensors, normalised to sum to one; there is no neighbour cut-off, so it is global IDW with power $p = 2$. Distances are Euclidean in the coordinate system the problem uses: grid-index units for Problem 4, and normalised $[-1, 1]$ latitude–longitude for Problem 6, not great-circle distance.

k-nearest neighbours. For Problem 4, $k = 8$ with weights $\propto \|\cdot\|^{-1}$, i.e. inverse distance to the *first* power. This differs from the IDW baseline in both the neighbour set and the exponent, so the two are distinct interpolants rather than the same interpolant with and without a cut-off. For Problem 6, $k = 10$ with scikit-learn's distance weighting, refitted per test day.

Power law (Problem 5). A two-point exponent computed per profile from the 10 m and 50 m sensors, $\alpha = \log(U_{50}/U_{10})/\log(50/10)$, extrapolated as $U(z) = U_{50}(z/50)^{\alpha}$. Nothing is fitted on training data.

Two-point log-height extrapolation (Problem 5). A straight line in U against $\log_{10} z$ through the 25 m and 50 m sensors, extrapolated to the query height. We do not call this the logarithmic wind profile: it contains no roughness length, no von Kármán constant, no friction velocity and no stability correction, and it is not derived from surface-layer similarity theory. It is included as the natural two-point extrapolant in log-height coordinates, which is what a practitioner without a stability estimate would use.

Nearest sensor (Problem 5). Returns the 50 m reading unchanged at every query height.

Climatology (Problem 6). The per-cell mean over the training days, broadcast to every test day. It is a training-period mean, not a seasonal or day-of-year climatology.

Ridge regression (Problem 6). A global two-feature linear map from the IDW-interpolated temperature and salinity at a cell to the sea-surface height at that cell, SSH $\approx a\hat{T} + b\hat{S} + c$, fitted on all training (day, cell) pairs with $\alpha = 1$. The regularisation strength was not selected by cross-validation; it is the library default and was left at that value.

KCN and the neural operators. KCN embeds each of a query point's k nearest sensors as a feature vector, scores them, applies a softmax over the neighbours and decodes the attention-weighted sum; $k = 12$ for Problems 4 and 6 and $k = 3$ for Problem 5, where the three input heights are the only sensors available. FNO is applied where the input has a natural grid: Problem 5, where the profile is an ordered one-dimensional sequence, and Problem 6, where the scattered sensors are first IDW-rasterised onto the 60×160 grid, which makes it a two-stage pipeline whose front end is itself one of the classical baselines. FNO is not applied to Problem 4, whose 32 scattered segments would require the same rasterisation step on an 18×18 grid.

S6 Baseline models

Two families of comparator appear in this work and they answer different questions. The classical estimators defined in Section S5.5, inverse-distance weighting, k-nearest neighbours, the analytic wind profiles and climatology, are what is actually deployed when an interior field is wanted and no governing model is

available at the point of use. They are the comparison the paper's claim rests on, and they apply only to Problems 4–6, where the inputs are samples of the field being reconstructed.

The operator baselines defined in this section, NOMAD, KCN and Geo-FNO in their various combinations, answer a narrower question: whether the reported accuracy is a property of the operator formulation or an artefact of one architecture. They are configured here in full so that the reader can judge whether each was given a fair budget, and their per-problem results appear in Sections S7 and S8.

S6.1 NOMAD (nonlinear manifold decoders)

NOMAD [4] approximates the operator $\mathcal{G} : \mathcal{U} \to \mathcal{S} \subset L^2(\mathcal{Y}; \mathbb{R}^m)$ with an encoder–approximator–decoder architecture whose decoder is nonlinear in latent coordinates. Given a mixed-modal input $\boldsymbol{u} \in \mathcal{U}$, an encoder $E : \mathcal{U} \to \mathbb{R}^p$ produces a feature vector $z = E(\boldsymbol{u})$, an approximator $A : \mathbb{R}^p \to \mathbb{R}^{n_{\text{NOMAD}}}$ maps z to latent coordinates $\zeta = A(z)$, and a decoder $D : \mathbb{R}^{n_{\text{NOMAD}}} \times \mathcal{Y} \to \mathbb{R}^m$ returns the m-channel field value at query locations. (We use n_{NOMAD} for NOMAD's latent dimension to avoid collision with n, which denotes the number of input modalities in the MIMONet section, and with n_x, which denotes the state-space dimension of classical observers.) The pointwise prediction is therefore

$$\hat{\mathbf{s}}(\mathbf{r} \mid \boldsymbol{u}) \;=\; D(\zeta, \mathbf{r}) \in \mathbb{R}^m, \qquad \zeta \in \mathbb{R}^{n_{\text{NOMAD}}},\ \mathbf{r} \in \mathcal{Y},$$

where m is the number of output channels, n_{NOMAD} the latent dimension, and p the encoder feature width; the per-case widths are listed in Table S7. The latent coordinates $\zeta \in \mathbb{R}^{n_{\text{NOMAD}}}$ are shared across all m output channels and the decoder $D(\zeta, \mathbf{r})$ returns the channels jointly at each query point; in this shared-latent sense NOMAD belongs to the same multi-field-decoding regime as MIMONet. For a batch of P query points $\{\mathbf{r}_p\}_{p=1}^{P}$, independent evaluations of $D(\zeta, \mathbf{r}_p)$ yield $\hat{\mathbf{s}} \in \mathbb{R}^{P \times m}$. Unlike linear-basis decoders that confine $\hat{\mathbf{s}}(\cdot)$ to a finite-dimensional linear subspace, the nonlinear map $D(\zeta, \mathbf{r})$ permits curved (nonlinear) output manifolds, which is advantageous for solution families with slowly decaying spectra or transport-dominated structure. The distinguishing architectural choice between NOMAD and MIMONet is the branch-fusion operator that produces the shared latent: NOMAD's encoder E aggregates multi-modal inputs additively, whereas MIMONet's branch fusion is multiplicative. Training uses standard pointwise losses over $\mathbf{r} \in \mathcal{Y}$, preserving query-based evaluation consistent with the notation above.

Table S7: **NOMAD architectures used in this study.** Layers are listed as input and subsequent hidden/output widths per MLP block. Branch features are broadcast across query points before concatenation with trunk features.

**Problem**	**Branch encoder(s)**	**Trunk**	**Combined head**	**Outputs**
LDC	$[90, 512, 512, 512, 512, 512]$	$[2, 512, 512, 512]$	$[1024, 512, 512, 512, 512, 3]$	$\{p,\ \lVert\boldsymbol{v}\rVert,\ k\}$
Subchannel	$[100, 128, 128]$, $[2, 128, 128]$	$[2, 128, 128]$	$[384, 128, 3]$	$\{T,\ k,\ \lVert\boldsymbol{v}\rVert\}$
Heat exchanger	$[100, 128, 128]$,$[2, 128, 128]$	$[2, 128, 128]$	$[384, 128, 4]$	$\{p,\ u_x,\ u_y,\ u_z\}$

S6.2 KCN (Kriging Convolutional Networks) + NOMAD

In our comparisons, we also evaluated a hybrid in which NOMAD's latent representation is decoded by a KCN-style local graph [5], rather than an MLP. In this hybrid, a NOMAD backbone first maps $\boldsymbol{u} \in \mathcal{U}$ to latent coordinates $\zeta \in \mathbb{R}^{n_{\text{NOMAD}}}$. For each query location $\mathbf{r} \in \mathcal{Y}$, a K-nearest-neighbour graph is formed over the query coordinates with edge weights given by an RBF kernel of bandwidth ϕ,

$$A_{jk} = \exp\Big(- \tfrac{\lVert \mathbf{r}_j - \mathbf{r}_k \rVert_2^2}{2\phi^2} \Big).$$

Node features concatenate coordinates and the latent, e.g. centre $[\mathbf{r}, \zeta]$ and neighbours $[\mathbf{x}_k, \zeta]$. The per-case configurations are given in Table S8. A shallow message-passing GCN (three `GCNConv` layers, width 32, ReLU, dropout as specified) aggregates these features and a final linear layer maps embeddings to m output channels:

$$\hat{\mathbf{s}}(\mathbf{r} \mid \boldsymbol{u}) \;=\; \Phi\big([\mathbf{r}, \zeta],\ \{[\mathbf{x}_k, \zeta]\}_{k=1}^{K},\ A \big) \in \mathbb{R}^m.$$

The hybrid is trained end-to-end with the same pointwise loss on $\mathcal{Y}$ as other baselines. At inference, only coordinates and the NOMAD features are used; no labels enter the decoder. Across all problems (LDC, subchannel, heat exchanger), the KCN decoder used a fixed $K = 5$ nearest neighbours. The RBF bandwidth ϕ was set automatically to the median of the K-NN distances computed on the training coordinates; the edge weights were $A_{jk} = \exp(-\|\mathbf{r}_j - \mathbf{r}_k\|_2^2/(2\phi^2))$ with $\gamma = 1/(2\phi^2)$ in the implementation.

Table S8: **KCN+NOMAD configurations.** "Branch" lists widths for each parallel branch MLP; "Trunk" lists query-MLP widths; "Head" lists the latent-fusion MLP prior to the KCN decoder. The KCN block (3 `GCNConv` layers, width 32) is shared across problems.

Problem	NOMAD Branch	NOMAD Trunk	NOMAD Head	KCN GNN	Outputs
LDC	$[90, 32, 32]$	$[2, 32]$	$[64, 32, 32]$	$3 \times$ `GCNConv` (32)	$\{p, \|\boldsymbol{v}\|, k\}$
Subchannel	$[100, 32, 32]$, $[2, 32, 32]$	$[2, 32]$	$[96, 32, 32]$	$3 \times$ `GCNConv` (32)	$\{T, k, \|\boldsymbol{v}\|\}$
Heat exchanger	$[100, 32, 32]$, $[2, 32, 32]$	$[2, 32]$	$[96, 32, 32]$	$3 \times$ `GCNConv` (32)	$\{p, u_x, u_y, u_z\}$

S6.3 GeoFNO + NOMAD

A hybrid spectral–manifold decoder was evaluated in which a NOMAD front end produces a coordinate-conditioned latent map that is further refined by a Geo-FNO [6]; the per-case configurations are listed in Table S9. Concretely, a NOMAD block maps inputs to an intermediate two-channel feature at query locations; these features are then lifted and passed through a Geo-FNO with four spectral convolution layers (2D FFT/linear/IFFT with learned low-rank weights) interleaved with 1×1 pointwise convolutions and bias terms. An inverse mapping network (`IPHI`) provides coordinate warping for nonuniform query sets. The final linear head projects to m output channels. Models were trained end-to-end with the same pointwise loss on $\mathcal{Y}$ as other baselines.

Table S9: **GeoFNO+NOMAD configurations per problem.** "Branch/Trunk/Head" list widths per MLP block in the NOMAD front end. The GeoFNO block uses 4 spectral layers and pointwise 1×1 convolutions with width 32; `IPHI` is used for coordinate warping.

Problem	NOMAD Branch	NOMAD Trunk	NOMAD Head	GeoFNO core	Outputs
LDC	$[90, 32, 32]$	$[2, 32]$	$[64, 32, 2]$	modes(16, 16), width 32	$\{p, \|\boldsymbol{v}\|, k\}$
Subchannel	$[100, 32, 32]$, $[2, 32, 32]$	$[2, 32]$	$[96, 32, 2]$	modes(16, 16), width 32	$\{T, k, \|\boldsymbol{v}\|\}$
Heat exchanger	$[100, 32, 32]$, $[2, 32, 32]$	$[2, 32]$	$[96, 32, 2]$	modes(16, 16), width 32	$\{p, u_x, u_y, u_z\}$

Notes: (i) The GeoFNO lift/projection uses `fc0`: 2→32, `fc1`: 32→128, `fc2`: 128→m with GELU activations; m equals the number of outputs per problem (3 for LDC/subchannel, 4 for heat exchanger). (ii) The NOMAD head outputs a two-channel feature per query, which serves as the GeoFNO input (`in_channels`=2). (iii) All runs used identical spectral mode counts (16, 16) and width 32; IPHI was enabled in all cases.

S6.4 FNO (Problems 5 and 6)

FNO is reported as a baseline on the two problems whose inputs carry a natural grid, and its configuration is given here for completeness; no FNO is run on Problems 1–4.

Table S10 gives both configurations. For Problem 5 the vertical profile is an ordered one-dimensional sequence, so a one-dimensional FNO is used directly on the three input heights. For Problem 6 the scattered sensors carry no grid, so the sensor values are first rasterised onto the 60×160 ocean grid by inverse-distance weighting and a two-dimensional FNO is applied to the result; the rasterisation stage is part of the baseline and is the reason this model is not simply an operator on the same inputs MIMONet receives.

On Problem 6 the parameter count is within 0.8% of MIMONet-NL's 2,795,907, so that comparison is capacity-matched. On Problem 5 it is not, as recorded in Section S8.2.

Table S10: **FNO baseline configurations.** Both use the standard lift–spectral–project structure with pointwise 1×1 convolutions in the residual path and GELU activations. "Modes" is the number of retained Fourier modes per dimension.

Problem	Variant	Lift width	Modes	Depth	Projection	Params
5. IEA-Wind towers	FNO1d	48	3	4	$48 \to 64 \to 1$	68,289
6. GLORYS12 ocean	FNO2d	26	16	4	$26 \to 128 \to 3$	2,775,703

S7 Complete results for the simulation problems

The main text quotes the per-channel errors for Problems 1–3 in prose. They are collected in Table S11 against the three operator baselines so that the comparison can be read in one place, and because the simulation problems are the ones for which no classical interpolant exists to compare against: their inputs are boundary data rather than samples of the output field, so the only available comparators are other operator formulations. These results establish that the framework is accurate on coupled multiphysics fields; the argument against what is deployed in practice is carried by Problems 4–6 in Section S8.

Table S11: **Reconstruction error on the three simulation problems.** Mean relative ℓ_2 error (%) per output channel on the held-out test set of each case, with the channel mean in the final column. Parameter counts are given because the baselines span two orders of magnitude in capacity and the comparison is therefore not capacity-controlled; KCN in particular is far smaller than the others by construction, as its neighbour graph carries most of its structure. Every entry was regenerated from the released checkpoints and agrees with the value quoted in the main text to within 0.1 percentage points; the harness is `Production/reproduce_accuracy.py`.

Model	Params	Per-channel error (%)				Mean (%)
(a) Lid-driven cavity						
		p	$\|\boldsymbol{v}\|$	k		
**MIMONet**	1.0 M	**2.1**	**5.0**	**2.8**		**3.3**
NOMAD	2.9 M	23.7	46.9	55.1		41.9
GeoFNO	2.7 M	49.8	82.4	71.7		68.0
KCN	10.4 K	27.6	6.0	41.5		25.0
(b) PWR subchannel						
		$\|\boldsymbol{v}\|$	T	k		
**MIMONet**	1.7 M	**2.2**	**0.27**	**4.2**		**2.2**
NOMAD	113 K	74.1	98.5	30.9		67.8
GeoFNO	2.7 M	70.2	98.6	47.7		72.2
KCN	12.9 K	91.5	8.3	59.2		53.0
(c) Heat exchanger						
		p	u_z	u_y	u_x	
**MIMONet**	1.7 M	**0.82**	**1.45**	**1.02**	**0.52**	**0.95**
NOMAD	113 K	11.5	26.3	17.5	10.1	16.4
GeoFNO	2.7 M	3.90	8.70	6.00	3.40	5.50
KCN	12.9 K	14.4	22.2	17.1	11.4	16.3

Two features are worth drawing out. The ordering among the baselines is not stable across the three problems: GeoFNO is the weakest on the lid-driven cavity and the subchannel but the strongest on the heat exchanger, and KCN is competitive on the single smooth channel of each case ($\|\boldsymbol{v}\|$ at 6.0% on the cavity, T at 8.3% on the subchannel) while failing on the others. This is the behaviour a locality-based estimator should show, and it is why the paper does not present a ranking among operator families. Second, the

capacity spread is large and uncontrolled: KCN carries between 10 and 13 thousand parameters, against 113 thousand to 2.9 million for the others, so its errors should be read as a statement about the locality of its construction rather than as a like-for-like comparison.

S8 Complete real-world results

The main text quotes single operating points from each of the three measured-data problems. The complete five-seed results are given here. Throughout, the quoted spread is the standard deviation over the five seeds computed with the population convention (dividing by n), which is the convention used in the main text; the classical baselines are deterministic given the sensor draw and the split, but both of those are seed-dependent for Problems 4 and 6, so those baselines carry a seed spread and it is reported; where a baseline uses no sensors and the split is fixed it is reported without a spread.

S8.1 Fuel-cell reconstruction across sensor coverage

Table S12 reports every coverage level of the sweep, and Fig. S1 plots it. The current-density channel J is the informative one: it is measured at all 324 segments, whereas the temperature channel is a bilinear upsampling of 81 measurements (Section S4.4) and is correspondingly easy for every method, so the temperature column is reported for completeness rather than as a discriminating comparison.

Table S12: **PEM fuel-cell reconstruction across sensor coverage.** Relative ℓ_2 error (%) on the held-out segments, mean ± s.d. over five seeds. Coverage is n_{obs} out of the 324 segments of the 18×18 current-density grid. KCN was run at the 32-sensor operating point only.

n_{obs}	Coverage	Method	J (%)	T (%)
6	1.9%	MIMONet	$\mathbf{3.91 \pm 0.98}$	$\mathbf{0.044 \pm 0.002}$
		IDW	35.51 ± 7.97	0.335 ± 0.038
		kNN	36.18 ± 6.26	0.356 ± 0.027
16	4.9%	MIMONet	$\mathbf{3.66 \pm 0.99}$	$\mathbf{0.039 \pm 0.004}$
		IDW	25.18 ± 2.04	0.269 ± 0.012
		kNN	30.07 ± 2.35	0.305 ± 0.015
32	9.9%	MIMONet	$\mathbf{3.77 \pm 1.02}$	$\mathbf{0.038 \pm 0.004}$
		KCN	12.29 ± 3.93	0.055 ± 0.003
		IDW	23.10 ± 1.60	0.241 ± 0.018
		kNN	23.74 ± 2.04	0.239 ± 0.021
81	25.0%	MIMONet	$\mathbf{3.55 \pm 0.92}$	$\mathbf{0.035 \pm 0.001}$
		IDW	18.56 ± 0.57	0.190 ± 0.013
		kNN	12.71 ± 0.82	0.131 ± 0.011
162	50.0%	MIMONet	$\mathbf{3.55 \pm 0.85}$	$\mathbf{0.034 \pm 0.002}$
		IDW	16.77 ± 1.08	0.168 ± 0.009
		kNN	8.48 ± 0.49	0.089 ± 0.006

The sweep shows two behaviours that the single operating point in the main text does not. MIMONet is nearly flat in coverage: the current-density error moves only from 3.91% at six sensors to 3.55% at 162, a 9% relative change across a 27-fold increase in sensor count. The classical interpolants are not: kNN improves from 36.18% to 8.48% over the same range. The advantage of the operator is therefore largest where sensors are sparsest, which is the regime the application is about, and it narrows steadily as the field becomes densely observed. At 50% coverage kNN is within a factor of 2.4 of MIMONet on J, and simple extrapolation of the trend suggests the two would meet somewhere beyond the coverage this cell provides. Reporting only the 10% point would leave that convergence invisible, which is why the full sweep is given

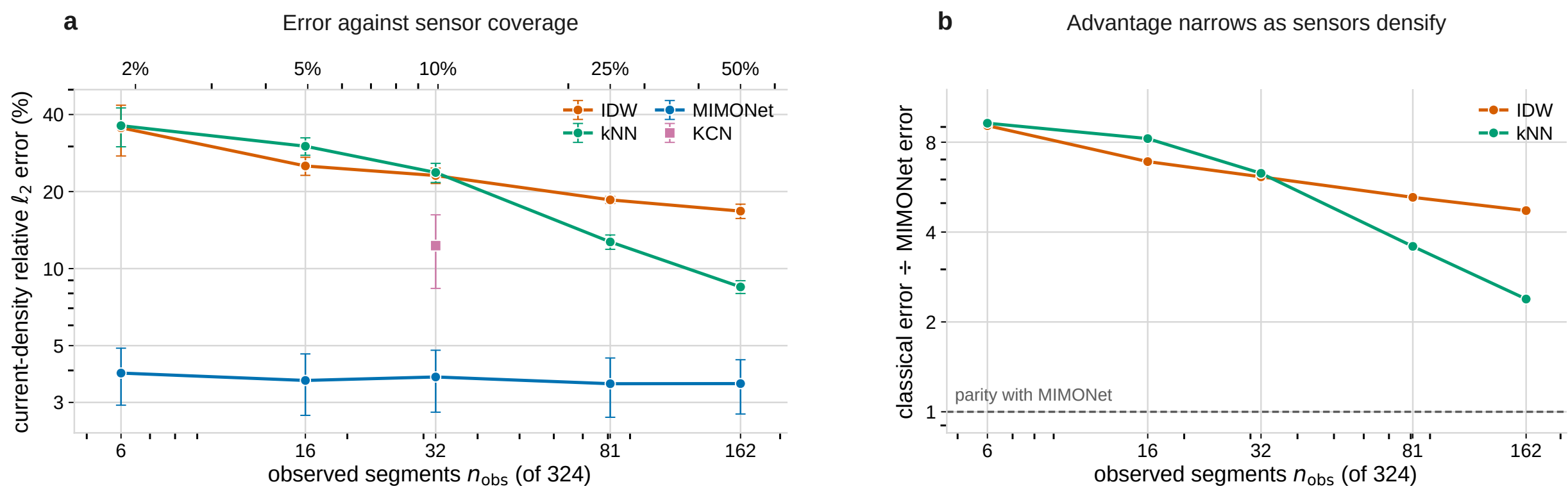

Figure S1: **Fuel-cell reconstruction against sensor coverage.** Current-density relative ℓ_2 error on the held-out segments, mean over five seeds with error bars at ±1 standard deviation. The upper axis of **a** gives coverage as a fraction of the 324 segments of the 18 × 18 mapping grid. Only the current-density channel is shown; the temperature channel is a bilinear upsampling of an 81-point measurement (Section S4.4) and does not discriminate between methods. **a**, Absolute error. MIMONet is nearly flat across a 27-fold change in sensor count, whereas the classical interpolants improve steeply; KCN was run at the 32-sensor operating point only. **b**, The same data as the ratio of classical error to MIMONet error. The operator's advantage is largest where sensors are sparsest, falling from roughly 9× at six sensors to 2.4× for kNN at 162, so the sparse regime that motivates virtual sensing is also the regime in which the advantage is real.

here. The ±1 standard deviation on the MIMONet current-density errors is around a quarter of the mean at every coverage level, driven by which segments the seed happens to select as sensors.

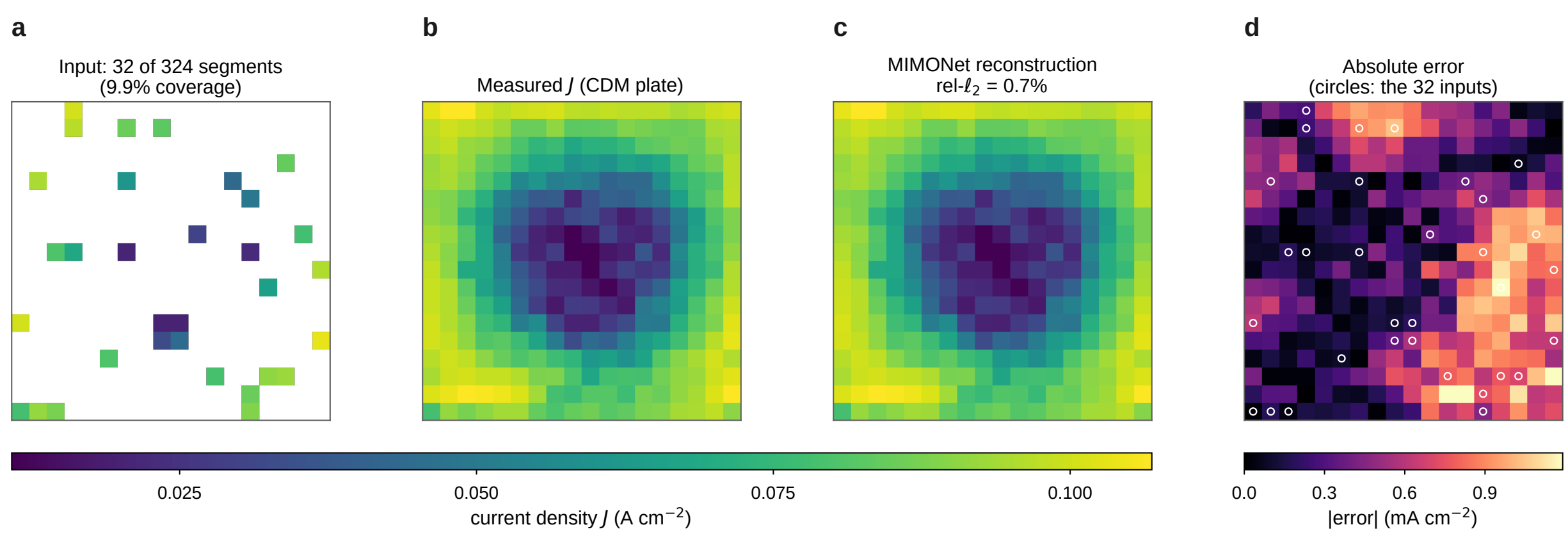

Figure S2: **Representative fuel-cell reconstruction and the spread behind it.** Seed-0 checkpoint, evaluated on the held-out frames at the 32-sensor operating point. **a**, The input actually supplied: 32 of the 324 segments, 9.9% coverage. **b**, The measured current density from the CDM plate on a held-out frame. **c**, The reconstruction at all 324 segments. **d**, Absolute error, with the 32 input locations circled; the error is not concentrated at the unobserved segments, which is the behaviour a spatial interpolant would show. Panels **b**–**d** show the median-error frame. That frame is not representative of the reported mean: the per-frame distribution is strongly right-skewed, with a median of 0.7% against a mean of 3.4% on this seed, so a frame carrying the mean error sits at roughly the 95th percentile. The headline current-density figure is set by a minority of difficult frames rather than by typical performance, and both statistics are given here so that neither reading stands alone.

A representative reconstruction is shown in Fig. S2, together with the distribution of per-frame errors

behind it. The distribution matters for reading the headline number. Errors are concentrated well below the mean, with a median of 0.7% against a mean of 3.4% on this seed, so the reported current-density error is set by a minority of frames rather than by the typical case. We report the mean throughout for consistency with the rest of the paper and with the main text, but a reader comparing a virtual sensor against an operational tolerance would want the distribution rather than its first moment, and the skew here is large enough that the two lead to different judgements. The spatial pattern of the error is also informative: it is not concentrated at the unobserved segments, which is what a spatial interpolant would produce, but follows the regions of steepest current-density gradient near the channel bends.

S8.2 Wind-profile reconstruction above the instrumented range

Table S13 reports all four query heights. The main text quotes the 80 m row.

Table S13: **Vertical wind-speed reconstruction at all four query heights.** Relative ℓ_2 error (%), mean ± s.d. over five seeds for the learned models; the analytic baselines are deterministic and carry no seed spread. Input sensors are at 10/25/50 m in every case. The test set contains 90,222 profiles at 60, 70 and 80 m but only 34,967 at 100 m, because two of the seven towers do not reach that height (Section S4.5); the 100 m column is therefore evaluated on a smaller and differently composed subset and is not directly comparable with the other three.

Method	60 m	70 m	80 m (hub)	100 m
MIMONet	$\mathbf{2.67 \pm 0.15}$	$\mathbf{3.79 \pm 0.06}$	$\mathbf{4.71 \pm 0.15}$	$\mathbf{14.70 \pm 0.59}$
FNO	4.05 ± 0.48	5.82 ± 1.03	7.30 ± 1.16	18.88 ± 1.00
KCN	11.03 ± 0.37	19.21 ± 0.40	25.28 ± 0.57	43.06 ± 1.14
Nearest sensor (50 m)	11.92	20.95	27.86	47.73
Two-point log-height extrapolation	12.43	21.86	28.95	52.49
Power law	12.89	23.68	34.30	95.92

The margin over the analytic profiles widens with distance above the topmost input sensor: at 60 m, ten metres above it, the operator is 4.5× better than the nearest-sensor estimate, and at 80 m it is 5.9× better. The reason is structural rather than a matter of tuning. The power law and the log-height extrapolation are two-parameter forms fitted to two sensors on each profile, so they can express only a single shape and cannot represent a profile that flattens, accelerates or reverses curvature above the instrumented range; when the atmosphere departs from that shape the error is bounded below by the model class, not by the data. An operator that has learned the distribution of profile shapes at these sites is not constrained in that way. This is the same argument as in the fuel-cell and ocean problems: the gain comes from replacing a fixed functional form with a learned one, not from a better fit within the same form.

FNO reaches comparable quality, which is expected and worth stating plainly, because the vertical profile is an ordered one-dimensional sequence and therefore a natural input for a spectral operator; the two learned models are not capacity-matched: MIMONet carries 207,617 parameters against FNO's 68,289 and KCN's 66,818, so FNO and KCN are matched to each other while MIMONet is roughly three times larger. We state this rather than presenting the comparison as capacity-controlled, and note that it cuts against the operator-versus-operator reading: FNO reaches comparable accuracy at a third of the budget. The comparison the paper rests on is against the classical estimators, where the gap is not attributable to capacity. Their similarity is the point rather than a complication: where an operator formulation applies, it is the formulation and not the particular architecture that produces the advance over the analytic profiles.

Figure S3 shows what the error numbers mean profile by profile, and it makes the limitation of the analytic baselines concrete rather than statistical. Both the power law and the log-height extrapolant are two-parameter forms fitted to two of the three input sensors, so the shape they extend above the mast is fixed by the shear those sensors show. When the measured profile reverses above 50 m, which happens in two of the three profiles drawn here at ordinary quantiles of the operator's error, no choice of the two parameters can follow it and both curves diverge from the measurement in the wrong direction. The operator is not constrained to a shape: it maps the three measured speeds together with site and diurnal context to a query height, so a reversal is representable provided the training data contains such profiles. That is the sense in

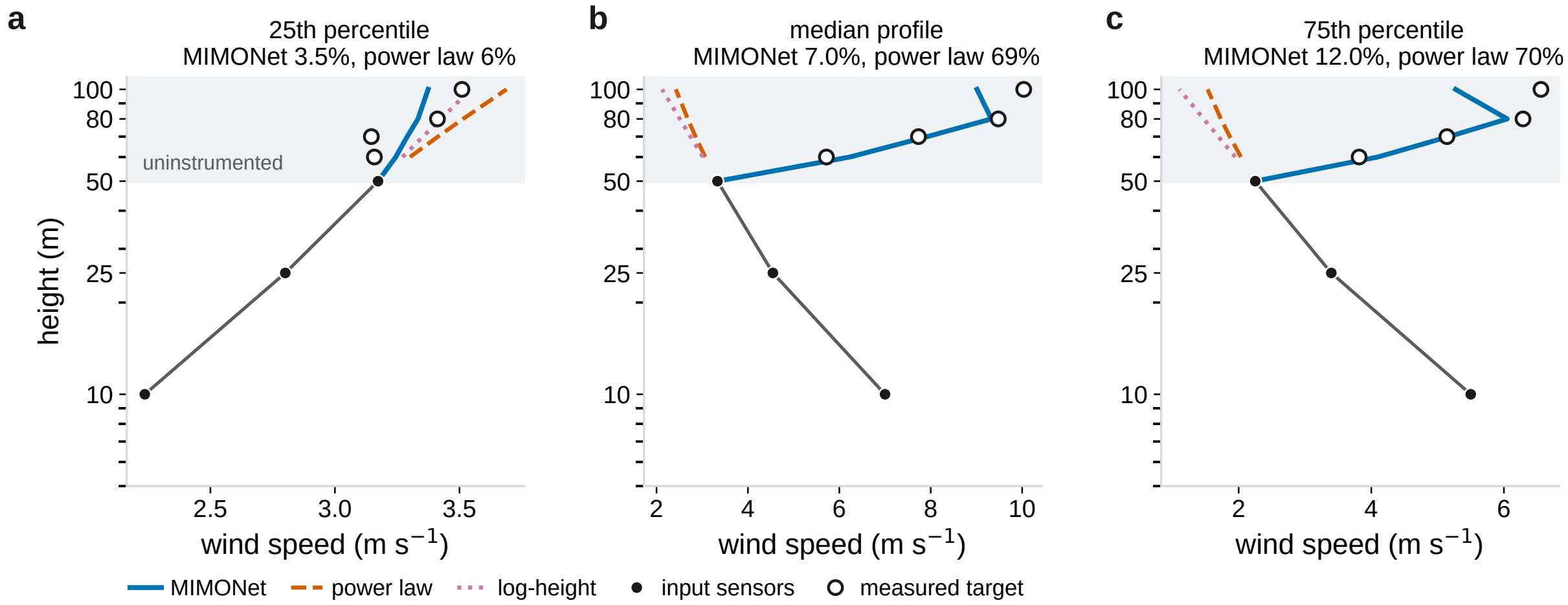

Figure S3: **Why a fixed-form profile cannot follow the measured one above the mast.** Seed-0 checkpoint on held-out profiles; the shaded band is the uninstrumented region above the topmost input sensor at 50 m. **a**–**c**, Three held-out profiles, with filled markers the three inputs at 10, 25 and 50 m, open markers the measured targets, and the grey segment below 50 m the measured input rather than a prediction. The three are taken at the 25th, 50th and 75th percentile of *MIMONet's own* error, so the selection does not depend on how the classical extrapolants happen to fare on them. Aggregate errors at every query height are in Table S13. In **b** and **c** the measured profile decreases with height through the instrumented range and then increases above it. The power law and the two-point log-height extrapolant are two-parameter shapes fitted to two of the three input sensors (10 and 50 m for the power law, 25 and 50 m for the log-height form), so they can only continue the shear those sensors show and both run the wrong way; the operator turns with the measurement. This is the qualitative content of the error numbers: the failure of the analytic profiles is structural rather than a matter of tuning.

which this problem is not an incremental improvement in fitting accuracy but a different class of estimator, and it is the same distinction, in a one-dimensional setting, that the ocean problem makes across modalities.

S8.3 Ocean-state reconstruction from disjoint sparse networks

Table S14 reports the three channels for the operators and classical estimators listed; the KCN and IDW-gridded FNO runs quoted in the main text are tabulated in Table S15. Sea-surface height carries no sensors of its own in any method's input, so the SSH column measures cross-modal inference throughout.

The comparison that carries the argument is the one against the classical estimators, and it separates into two very different situations. On temperature and salinity, which are directly sampled, interpolation is a well-posed problem and the classical methods do it competently; the operator improves on them by roughly a factor of two, which is a useful but incremental gain. Sea-surface height is the case the framework exists for, and it is the concrete instance of the disjoint sensing and reconstruction domains identified in the Introduction: the quantity to be recovered is of a different modality from every quantity observed. A spatial interpolant is defined by samples of the field it estimates, so with no SSH sensor anywhere IDW and kNN have no argument to take; this is a property of that estimator class, not a claim that SSH is unrecoverable by any classical route, and a data-assimilating ocean model with an explicit governing system would be a different matter. Within the classes that are deployed for this task, what remains is to predict the training-period mean (37.71%) or to regress SSH on the interpolated temperature and salinity, which at 58.18% and 59.44% depending on the interpolant is worse than the mean and shows that a global linear map does not capture the relationship. Read as complete pipelines, the three classical routes average 21.99% (kNN for T and S with its ridge for SSH), 23.35% (IDW throughout) and 18.83% (climatology throughout) across the three channels, against 4.22% for the operator. Recovering SSH at 9.11% from temperature and salinity sensors alone is therefore not an incremental improvement within an existing estimator class; it is a cross-modal

Table S14: **North Atlantic ocean-state reconstruction.** Relative ℓ_2 error (%) per channel on the 60 held-out days, mean ± s.d. over five seeds, under the random day split used in the main text. Inputs are 400 temperature and 400 salinity sensors at disjoint locations; no method receives any SSH observation. Dashes mark combinations that are undefined: IDW and kNN interpolate a field from its own sensors and have none for SSH, so the SSH entry for each of those rows is a ridge regression of SSH on the temperature and salinity fields interpolated by that same method, reported on its own line. The two ridge variants differ only in which interpolant supplies their inputs.

Method	Params	T (%)	S (%)	SSH (%)
MIMONet-NL	2,795,907	$\mathbf{3.10 \pm 0.06}$	$\mathbf{0.46 \pm 0.01}$	$\mathbf{9.11 \pm 0.17}$
NOMAD	2,943,363	3.49 ± 0.10	0.51 ± 0.01	10.72 ± 0.39
MIMONet (linear decoder)	2,412,547	3.54 ± 0.11	0.53 ± 0.00	11.05 ± 0.25
kNN ($k = 10$)	—	6.79 ± 0.32	0.99 ± 0.04	see below
IDW ($p = 2$)	—	9.26 ± 0.49	1.35 ± 0.06	see below
Ridge on kNN-interpolated T, S	—	—	—	58.18 ± 0.62
Ridge on IDW-interpolated T, S	—	—	—	59.44 ± 1.02
Climatology	—	17.68 ± 0.43	1.09 ± 0.01	37.71 ± 0.29

reconstruction obtained from a single amortised forward pass, with no governing model evaluated online. That is the claim the ocean problem is here to support, and Section S8.4 examines the conditions under which it holds.

The three operator variants are reported for completeness and to document the design choice, not as a ranking: MIMONet-NL, NOMAD and the linear-readout MIMONet span 9.11–11.05% on SSH, a spread far smaller than the gap between any of them and the classical alternatives. The nonlinear decoder is retained because it is the best of the three at comparable parameter count (Section S5.1), but nothing in the argument of this paper depends on which operator architecture wins that comparison.

S8.3.1 Other learned methods on the ocean problem

Two further learned baselines were run on this problem and are reported here because the main text quotes them and because one of them is informative about what the operator formulation is doing.

Table S15: **Learned baselines on the GLORYS12 problem, random day split.** Relative ℓ_2 error (%) per channel, mean ± s.d. over five seeds. The FNO is applied to sensor values first rasterised onto the 60×160 grid by inverse-distance weighting, and is capacity-matched to MIMONet-NL. KCN operates directly on the scattered sensors through a learned neighbour graph.

Method	T (%)	S (%)	SSH (%)
MIMONet-NL (reported)	3.10 ± 0.06	0.46 ± 0.01	9.11 ± 0.17
IDW-gridded FNO	3.10 ± 0.07	0.47 ± 0.01	9.58 ± 0.08
KCN	5.77 ± 0.23	0.86 ± 0.01	37.84 ± 0.28
Climatology (reference)	17.68 ± 0.43	1.09 ± 0.01	37.71 ± 0.29

The FNO result is stated plainly: on this problem it is indistinguishable from MIMONet-NL on the two sampled channels and within half a percentage point on sea-surface height. We do not present this as a defeat or a victory, because the paper's claim is not that one operator architecture outperforms another. It is the opposite of a complication for the argument: two independently constructed operator formulations both recover a field for which no sensor exists, which is stronger evidence that the capability follows from the operator formulation than a result from a single architecture would be.

KCN is the informative case, and it is why this table is included rather than omitted. It reconstructs the two sampled channels competently, as a learned interpolator should, but its sea-surface height error of

37.84% is statistically indistinguishable from the climatology value of 37.71%: it has learned to predict the training-period mean and nothing more. KCN builds its estimate from a neighbourhood graph over the sensor locations, so it is a learned interpolant rather than an operator between function spaces, and on a channel with no sensors in the neighbourhood there is nothing for that construction to interpolate. Cross-modal recovery is therefore not a generic consequence of using a neural network on this data, and it is not obtained by any method that remains tied to the geometry of the sensor network. That is the distinction the paper draws, and this row is the control that establishes it.

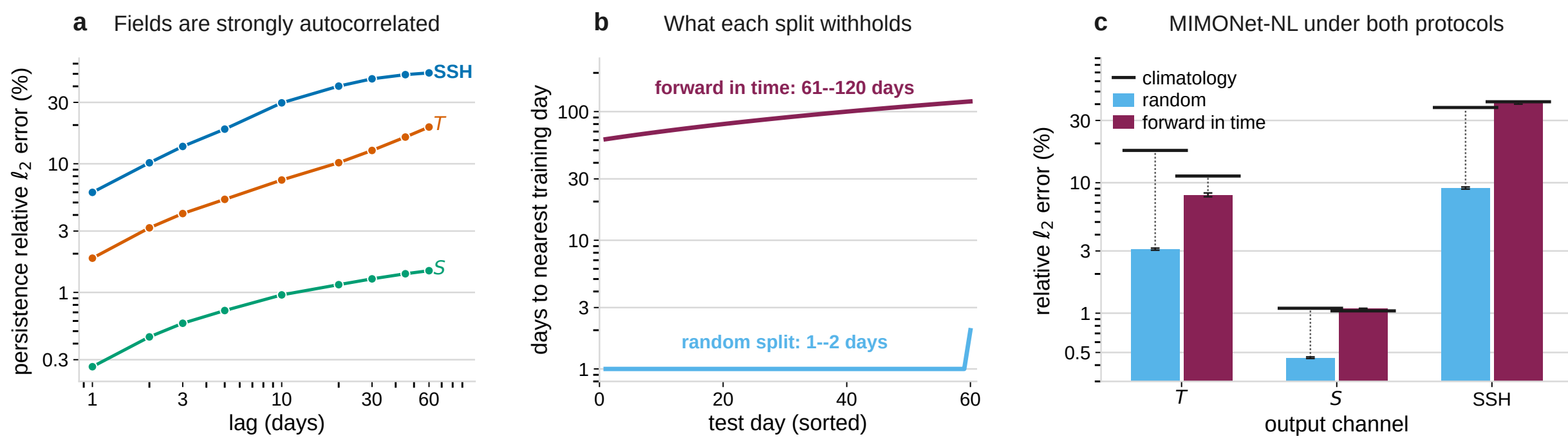

Figure S4: **Why the day split matters for the ocean problem.** **a**, Persistence error against lag: the relative ℓ_2 error incurred by carrying a field forward by the stated number of days, averaged over the record. Daily fields are strongly autocorrelated, so a nearby day is already an informative predictor. **b**, Distance from each test day to the nearest training day, sorted, under the two protocols. The random split leaves every test day within one or two days of a training day; the forward-in-time split places them 61 to 120 days away. **c**, MIMONet-NL under both protocols, per channel, with the horizontal rules marking the climatology of the same protocol. Persistence is not a competing method and is not plotted as one: it requires the complete field on an adjacent day, whereas the operator observes 800 point sensors on the target day and never sees another day.

S8.4 Forward-in-time robustness check for the ocean problem

The ocean results above use the random day split described in Section S4.6. Because daily ocean fields are strongly autocorrelated, that protocol places test days next to training days, and it is worth establishing what the reported numbers do and do not demonstrate. This section reports a re-run under a forward-in-time split, and states plainly which parts of the ocean claim survive it.

Why the split is consequential here. Figure S4a measures the autocorrelation directly: carrying a field forward by one day gives a relative ℓ_2 error of 1.8% for temperature, 0.3% for salinity and 5.9% for sea-surface height, rising to 19%, 1.5% and 51% at a lag of sixty days. Figure S4b shows what each protocol withholds: under the random split every test day has a training day within one or two days, whereas under the forward-in-time split the nearest training day is 61 to 120 days away. The random split therefore evaluates reconstruction of states that lie close to states already seen, and the forward split removes that proximity.

We note for completeness that carrying a neighbouring field forward is not a competing method and is not offered as a baseline. It requires the complete field on the adjacent day, all 9,457 cells in all three channels, whereas the operator observes 800 point sensors on the target day and never sees another day at training or inference. Panel a bounds how much novelty a test day carries; it does not describe an estimator the operator is being compared against.

Protocol. The re-run is identical to the reported experiment except in the assignment of days: training uses days 1–610, validation days 611–670 and testing days 671–730, i.e. the last two months of the record. Sensor networks, architecture, optimisation schedule, normalisation and seeds are unchanged, and five seeds

are run as before. The classical baselines are recomputed on the same split. Table S16 reports the two protocols side by side.

Table S16: **Ocean reconstruction under both day splits.** Relative ℓ_2 error (%) per channel, mean ± s.d. over five seeds; the classical estimators are deterministic given the split and the sensor draw. Under the forward-in-time protocol the split is fixed but the sensor draw is still seed-dependent, so kNN and IDW retain a spread; climatology uses no sensors at all and is therefore a single value under that protocol. IDW and kNN have no sea-surface-height sensors to interpolate, so no entry exists for them in that column.

	Random day split (reported)			Forward in time		
Method	T (%)	S (%)	SSH (%)	T (%)	S (%)	SSH (%)
MIMONet-NL	3.10 ± 0.06	0.46 ± 0.01	9.11 ± 0.17	8.08 ± 0.27	1.08 ± 0.01	41.07 ± 1.02
NOMAD	3.49 ± 0.10	0.51 ± 0.01	10.72 ± 0.39	8.10 ± 0.16	1.08 ± 0.01	40.37 ± 0.64
kNN	6.79 ± 0.32	0.99 ± 0.04	—	7.40 ± 0.28	1.02 ± 0.04	—
IDW	9.26 ± 0.49	1.35 ± 0.06	—	9.81 ± 0.48	1.37 ± 0.06	—
Ridge on interpolated T, S	—	—	59.44 ± 1.02	—	—	59.82 ± 0.24
Climatology	17.68 ± 0.43	1.09 ± 0.01	37.71 ± 0.29	11.26	1.04	41.74

What the re-run shows. Three things, and they point in different directions.

First, the degradation is a property of the protocol and of the problem, not of the architecture. MIMONet-NL and NOMAD are statistically indistinguishable under the forward split on all three channels (8.08 against 8.10, 1.08 against 1.08, 41.07 against 40.37), although they differed under the random split, most visibly on sea-surface height (9.11 against 10.72). Whatever the forward split removes, it removes from both operators equally, so the effect cannot be attributed to a particular fusion rule or decoder.

Second, on the directly observed channels the ordering against the classical interpolants reverses. Under the random split the operators improve on kNN by roughly a factor of two on temperature; under the forward split kNN is the more accurate of the two (7.40% against 8.08%), and on salinity the two are level. Where a field is sampled at 400 points and the task is spatial interpolation, a local estimator that fits each test day independently is not disadvantaged by temporal distance, whereas a trained operator is. This is a real limitation and we state it as one: the operator's advantage on directly observed channels, as measured here, depends on the test states resembling the training states.

Third, sea-surface height behaves differently from the other two, and the distinction is the one that matters for the claim this problem supports. No classical *interpolant* of the classes compared here can attempt it: IDW and kNN are defined by samples of the field they estimate and there are none, which is why their SSH entries are empty under both protocols. This is a statement about that estimator class and not a claim that SSH is unrecoverable by any classical route. The only classical options are climatology and a regression from interpolated temperature and salinity, and the regression is worse than climatology under both splits (59.44% and 59.82% against 37.71% and 41.74%). The operator remains the only method that produces a cross-modal estimate at all, and Fig. S5 shows what that difference looks like on a single day. What does not survive the forward split is the *margin*: at 41.07% against a climatology of 41.74% the operator is level with the trivial predictor, where under the random split it was better by a factor of 4.1.

What we conclude. The qualitative capability the ocean problem is included to demonstrate, that an operator can infer a field for which no sensor exists by using the learned coupling to fields that are sensed, is demonstrated under the random day split. Under the forward split it is not: at 41.07 ± 1.02% against a climatology of 41.74% the SSH estimate is not statistically distinguishable from the trivial predictor. We state this in the same terms used for KCN in Section S8.3.1, because the standard has to be the same: producing a cross-modal estimate at all is a property of the estimator's type signature, not evidence of skill, and we have not measured whether the forward-split output carries day-specific information. An anomaly correlation against the daily SSH anomaly would settle it in one number; we have not run it. What the two protocols jointly establish is that the operator formulation admits cross-modal reconstruction where the compared interpolants do not, and that the accuracy of that reconstruction depends on the test states

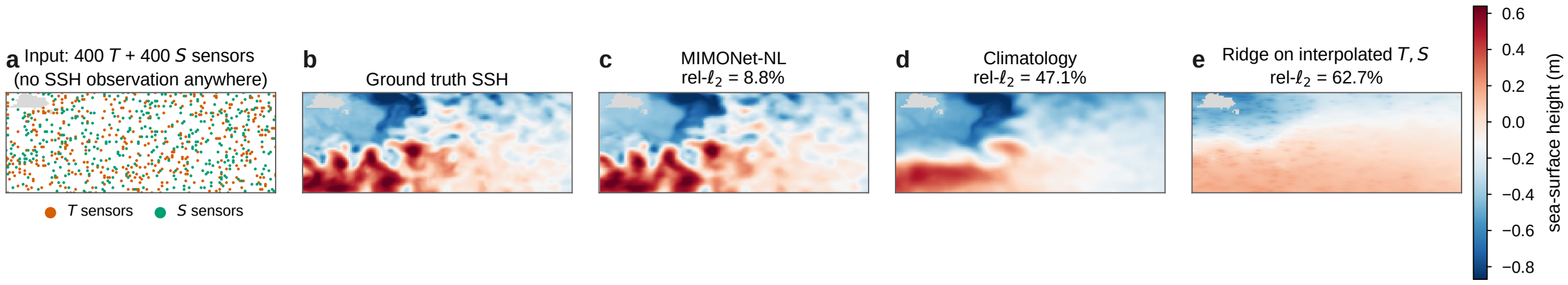

Figure S5: **Cross-modal reconstruction of an unobserved field.** Sea-surface height on a held-out day of the reported run, the day at the median SSH error of the sixty test days. **a**, The entire input: 400 temperature and 400 salinity point sensors at disjoint locations. No SSH value is supplied anywhere, which is why inverse-distance weighting and k-nearest neighbours have no entry for this field: an interpolant is defined by samples of the quantity it interpolates, and here there are none. This is the disjoint sensing and reconstruction regime of the Introduction, in its sharpest form. **b**, Ground truth. **c**, MIMONet-NL, reconstructing SSH from the temperature and salinity sensors alone through the learned coupling. **d**, Climatology, the training-period mean, which is the same field on every day and therefore cannot express the day's mesoscale structure at all. **e**, Ridge regression from the interpolated temperature and salinity, the only route among the classical estimators compared here that uses the day's observations, which is worse than the climatology. All panels share the colour scale. The point of the comparison is qualitative: the front position and the eddy field in **c** are recovered from measurements of different physical quantities, whereas **d** and **e** show what remains available without an operator.

resembling the training states. A data-assimilating ocean model with an explicit governing system would be a different matter; the contrast drawn here is with the interpolants that are actually deployed when no such model is available at the point of use. The quantitative figure quoted in the main text, a 4.1× reduction against climatology, is specific to the random day split and should be read as such. We attribute the loss under the forward split to the temperature–salinity-to-SSH relationship being calibrated to the mean state of the training period: sea-surface height carries a slowly varying component that the operator cannot observe through its inputs, so a systematic offset accumulates as the test period moves away from the training period. Recalibration on recent data, which is available in any operational setting, is the natural remedy, and quantifying how often it would be required is a well-defined question that we leave open.

Limitation of this check. The forward test window is the last two months of the record, November and December 2018. Temporal distance from the training period and the particular season are therefore confounded, and this experiment cannot separate them. Splitting the record into contiguous blocks and rotating the test window would do so; we have not run it, and the numbers above should be read as characterising a two-month-ahead gap at this season rather than temporal extrapolation in general.

S9 Uncertainty, robustness and data efficiency

A virtual sensor reports values at locations where, by construction, no measurement exists to check them against. An interval is therefore not a refinement but a precondition for using the output at all, and the same argument applies to knowing how the estimate degrades when the instrumentation does. This section reports the calibration, noise-robustness, data-efficiency and extrapolation studies together, because they answer one question: under what conditions the reconstruction should be trusted.

One restriction applies throughout and is stated once here rather than repeated. Every study in this section was run on the heat-exchanger case. No uncertainty estimate, noise sweep or out-of-distribution result is reported for the other five problems, and the properties established here should not be assumed to transfer to them.

S9.1 Conformal prediction for operator outputs

The Introduction lists calibrated uncertainty among the properties an operational virtual sensor must supply, and it matters most exactly where the framework is aimed: at interior locations where no measurement exists against which a prediction could be checked. An interval is the only quantitative statement of trustworthiness available there. Conformal prediction provides distribution-free, finite-sample valid uncertainty quantification. We extend the standard framework to spatially distributed operator outputs.

Theorem 3 (Valid Coverage for Conformal Intervals). *Let $\{(\boldsymbol{u}^{(k)}, \mathbf{s}^{(k)})\}_{k=1}^{N_{cal}}$ be a held-out calibration set exchangeable with the test distribution. Define normalised residuals*

$$z^{(k)}(\mathbf{r}) = \frac{|s^{(k)}(\mathbf{r}) - \hat{s}^{(k)}(\mathbf{r})|}{\sigma_G'^{(k)}(\mathbf{r}) + \epsilon},$$

where $\sigma_G' = s_c \cdot \sigma_G$ is the variance-rescaled predictive standard deviation from MC dropout, σ_G being the raw MC-dropout standard deviation and s_c a per-channel scaling factor fixed on the same calibration set (using s_c rather than α here to free the symbol α for the conformal miscoverage level). Normalising the score by the same quantity that scales the interval is what makes the two steps consistent. Let $q_{1-\alpha}$ be the $\lceil(1-\alpha)(N_{cal}+1)\rceil$-th smallest order statistic of $\{z^{(k)}(\mathbf{r})\}$ over the calibration set (the standard split-conformal quantile). Then the conformal prediction interval

$$\mathcal{C}(\mathbf{r}) = [\hat{s}(\mathbf{r}) - q_{1-\alpha}\sigma_G'(\mathbf{r}),\ \hat{s}(\mathbf{r}) + q_{1-\alpha}\sigma_G'(\mathbf{r})],$$

satisfies the split-conformal coverage guarantee

$$1 - \alpha \ \leq\ \mathbb{P}[s_{test}(\mathbf{r}) \in \mathcal{C}(\mathbf{r})] \ \leq\ 1 - \alpha + \frac{1}{N_{cal} + 1}$$

for any test point exchangeable with the calibration set [7, 8].

Implementation. On the heat-exchanger case the 1,546 samples are split 70/10/20 into 1,082 training, 154 calibration and 310 test cases, so $N_{\text{cal}} = 154$. The scaling factor s_c is the per-channel standard deviation of the raw z-scores $(\mathbf{s} - \hat{\mathbf{s}})/\sigma_G$ on the calibration set, and $q_{1-\alpha}$ is then taken per channel from the residuals of all $154 \times 3{,}977 = 612{,}458$ calibration (sample, node) pairs pooled together, at $\alpha = 0.05$. Two consequences of pooling should be stated. First, the coverage delivered is *marginal* over the field rather than pointwise at each $\mathbf{r}$, which is the quantity reported in the main text. Second, nodes within one reconstructed field are spatially correlated, so the pooled residuals are not exchangeable in the strict sense that the finite-sample guarantee assumes; the exchangeable units are the 154 calibration samples. The guarantee should therefore be read as holding to the accuracy with which pooled nodes approximate exchangeable draws, and it is the measured coverage, not the bound, that establishes calibration here. Table S17 gives the per-channel calibration constants, the coverage before and after rescaling, and the resulting interval half-width.

Two features of the table are worth reading together. The uncalibrated intervals are not uniformly wrong in the same direction: MC-Dropout is over-dispersed on pressure (97.79% coverage, $s_c = 0.77$, so the raw spread is shrunk) and slightly under-covering on u_y (94.16%), where $s_c = 1.00$ says the raw dropout spread is already correctly scaled and the correction comes from the residual tail through the quantile instead. A single global inflation factor would therefore not calibrate all four channels, which is why the scaling is fitted per channel. After calibration every channel sits within 0.25 percentage points of nominal, and the half-widths differ by roughly 28% across channels, so the calibrated intervals also carry information about which channels the model is genuinely less certain about.

Spatial considerations. What is delivered and measured above is *marginal* coverage over the field. Simultaneous coverage at every $\mathbf{r} \in \mathcal{Y}$ is a strictly stronger requirement and would need more conservative quantiles, obtained for instance by a Bonferroni correction or by calibrating on supremum rather than pointwise residuals. Neither is applied here, and no simultaneous guarantee is claimed.

Table S17: **Conformal calibration on the heat-exchanger case.** All quantities are per output channel, computed on the $N_{\text{cal}} = 154$ calibration samples and evaluated on the 310-case test set at $\alpha = 0.05$. s_c rescales the MC-Dropout standard deviation so that the normalised residuals have unit spread; $q_{1-\alpha}$ is the conformal quantile of those rescaled residuals. The calibrated 95% interval is $\hat{\mathbf{s}} \pm q_{1-\alpha} s_c \sigma_G$, so the last column is the interval half-width expressed as a multiple of the raw MC-Dropout standard deviation. Values are from the reproduction script `Production/analyses/uq_repro.py`; the MC-Dropout ensemble is redrawn at each run, so the final digit of the coverages moves by a few hundredths between runs.

Channel	s_c	$q_{1-\alpha}$	Coverage, uncalibrated (%)	Coverage, conformal (%)	Half-width ($\times\sigma_G$)
p	0.767	2.132	97.79	95.21	1.63
u_z	0.901	2.094	95.39	94.97	1.89
u_y	1.003	2.083	94.16	94.96	2.09
u_x	0.928	2.100	95.08	94.95	1.95
Pooled	—	—	—	95.02	—

S9.2 Stability under sensor noise: Lipschitz analysis

Virtual sensing under degraded instrumentation requires that the learned operator not amplify input perturbations. We treat this as a property that the operator is required to have and is verified to have *by measurement*, not as one guaranteed by the training procedure: empirical risk minimisation places no constraint on the Lipschitz constant of the learned map, and the cross-architecture comparison in Section S9.6 measures the related but distinct quantity of RMSE inflation under input noise, finding it to vary by more than two orders of magnitude across operators trained on the same data. No Lipschitz constant was measured for those other architectures, so the two quantities should not be equated.

Let $\tilde{\boldsymbol{u}} = \boldsymbol{u} + \boldsymbol{\eta}$, with $\boldsymbol{\eta} \sim \mathcal{N}(\mathbf{0}, \sigma^2_{\text{noise}}\mathbf{I})$, denote a noisy input, let $\hat{\mathcal{G}}_{\boldsymbol{\omega}}$ be the sub-network realised by dropout mask $\boldsymbol{\omega}$, and let $\bar{\mathcal{G}}(\boldsymbol{u}) = \mathbb{E}_{\boldsymbol{\omega}}[\hat{\mathcal{G}}_{\boldsymbol{\omega}}(\boldsymbol{u})]$ be the MC-Dropout estimator. Since the norm is convex, Jensen's inequality gives

$$\left\|\bar{\mathcal{G}}(\tilde{\boldsymbol{u}}) - \bar{\mathcal{G}}(\boldsymbol{u})\right\|_{\mathcal{S}} \leq \mathbb{E}_{\boldsymbol{\omega}}\left\|\hat{\mathcal{G}}_{\boldsymbol{\omega}}(\tilde{\boldsymbol{u}}) - \hat{\mathcal{G}}_{\boldsymbol{\omega}}(\boldsymbol{u})\right\|_{\mathcal{S}} \leq \mathbb{E}_{\boldsymbol{\omega}}[L_{\boldsymbol{\omega}}]\,\|\boldsymbol{\eta}\|_{\mathcal{U}},$$

where $L_{\boldsymbol{\omega}}$ is the Lipschitz constant of the masked sub-network. The content of this inequality is exact but limited: averaging over dropout masks cannot make the estimator more sensitive to a perturbation than the *average* of its members. It does not establish that the ensemble mean is less sensitive than the deterministic full network, which is a different quantity, and it yields no numerical bound, since $\mathbb{E}_{\boldsymbol{\omega}}[L_{\boldsymbol{\omega}}]$ is not accessible in closed form. Both the magnitude of the constant and any ordering between inference modes are therefore established empirically, in Section S9.4 and in the main text.

S9.3 Sample complexity and convergence

Amortisation moves cost from inference to training, so the size of that up-front cost is part of the argument: a virtual sensor that needs an unattainable number of high-fidelity solutions is not deployable however fast it runs afterwards. This subsection measures how the error falls with the number of training solutions.

The sample-complexity sweep reports the held-out test-set error as a function of the training-pool size N_{train}. MIMONet was trained from a fresh, identically-seeded initialisation on four nested random subsets of each case's training pool; the fifth and largest point is the published checkpoint rather than a sweep run, which has a consequence for the LDC case that is stated below. The three cases reported here are the PWR subchannel (pool size 3,200, $N_{\text{train}} \in \{320, 800, 1{,}600, 2{,}400, 3{,}200\}$), the heat exchanger (pool size 1,082 from the 70/10/20 split, $N_{\text{train}} \in \{108, 270, 541, 812, 1{,}082\}$), and the LDC ($N_{\text{train}} \in \{320, 800, 1{,}600, 2{,}400, 3{,}200\}$ subsampled from the combined LDC training and validation pool of 3,949 = 3,159+790 samples). The swept points share the architecture, optimiser (Adam, lr $= 10^{-3}$, weight decay 10^{-6}), and batch size (4). The LR schedule is `ReduceLROnPlateau` (patience 50, factor 0.5, $\text{lr}_{\min} = 10^{-6}$) for subchannel and heat exchanger, and `CosineAnnealingLR` ($\eta_{\min} = 10^{-6}$, $T_{\max} = 500$) for LDC (the plateau detector was found unreliable across N on LDC, so a deterministic cosine schedule is used to remove the dependency on val-loss noise).

A fixed held-out validation split (800 / 154 / 749 samples for subchannel / heat exchanger / LDC) is held constant across the swept conditions in each case; the LDC sweep therefore partitions the 3,949-sample pool as 3,200/749 rather than as the 3,159/790 split of record given in Table S2. Test errors are evaluated on the fixed, disjoint test set of each case (1,000 / 310 / 988 samples).

For $N_{\text{train}} = N_{\max}$ the headline checkpoint reported throughout the main text is reused to anchor the curve at the published per-channel relative ℓ_2 values, and the consequence differs by case. On the subchannel and heat exchanger the headline configuration coincides with the sweep configuration, so the endpoint is continuous with the other four points. On the LDC it is not: the headline model was trained with batch size 8, weight decay 10^{-7} and a plateau schedule on the 3,159-sample split (Table S6), whereas the swept points use batch size 4, weight decay 10^{-6} and cosine annealing. The LDC endpoint is thus plotted at a nominal $N_{\text{train}} = 3{,}200$ although the checkpoint was trained on 3,159 samples, and it differs from the other four points in optimiser configuration as well. The fitted exponent $\gamma_{\text{LDC}} = 0.58 \pm 0.23$ is computed across that seam and should be read as indicative rather than as a controlled measurement. The subchannel and heat-exchanger exponents are unaffected.

The convergence curves for all three cases are shown in Fig. S6. All three fitted exponents ($\gamma_{\text{Sub}} = 0.66\pm0.12$, $\gamma_{\text{HX}} = 0.43\pm0.11$, $\gamma_{\text{LDC}} = 0.58\pm0.23$) are consistent with the operator-approximation rate bounds for smooth neural-operator classes [9,10]. The subchannel curve flattens between $N_{\text{train}} = 1{,}600$ and $N_{\text{train}} = 2{,}400$ (log–log slope -0.10 over a 1.5× pool-size increase), then resumes its decline to 2.2% at the full pool. We do not read the full-pool point as convergence: the error falls by 47% over the final 1.33× of data, so the curve is still descending steeply at its endpoint and the pool size cannot be described as saturating. The heat-exchanger curve descends monotonically from 2.6% at $N_{\text{train}} = 108$ to the headline 0.95% at $N_{\text{train}} = 1{,}082$ with a smaller exponent, reflecting that the HX virtual-sensing problem is data-efficient (low-error regime is reached with $\sim 5\times$ fewer training samples than for subchannel) while still benefiting from additional data up to the full pool. The LDC curve descends from 15.6% at $N_{\text{train}} = 320$ to the headline 3.3% at $N_{\text{train}} = 3{,}200$. One data point, $N_{\text{train}} = 2{,}400$, sits above the power-law prediction (measured 6.4% vs. fit $\approx 4.8\%$, a +1.6 percentage-point residual); the confidence interval on the fitted exponent (±0.23) reflects the additional fitting uncertainty introduced by this point but is a statement about the exponent, not a prediction interval that contains the residual itself. We attribute the elevation at $N_{\text{train}} = 2{,}400$ to a known sensitivity of the LDC optimisation to which 2,400-sample subset is drawn from the 3,949-sample pool (the LDC training-pool re-uses a deterministic random permutation seed of 42; different seeds shift this point but not the overall trend), rather than to a violation of the power-law regime. The endpoint at $N_{\text{train}} = 3{,}200$ matches the headline value reported in the main text.

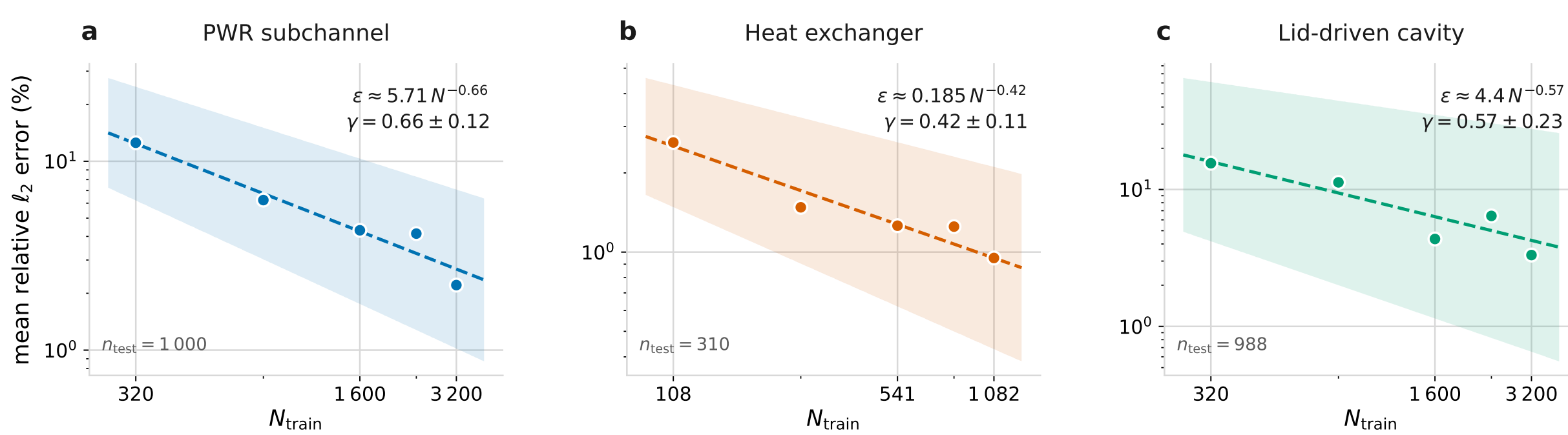

Figure S6: **Sample-complexity convergence.** Mean relative ℓ_2 error on the held-out test set as a function of N_{train}, with a power-law fit $\epsilon(N) = a \cdot N^{-\gamma}$. **(a)** PWR subchannel (1,000-sample test set). Channel-mean errors 12.5%, 6.2%, 4.3%, 4.1%, 2.2% at $N_{\text{train}} = 320$, 800, 1,600, 2,400, 3,200; fit $\epsilon \approx 571\,N^{-0.66}$, $\gamma = 0.66\pm0.12$. **(b)** Heat exchanger (310-sample test set). Channel-mean errors 2.61%, 1.48%, 1.26%, 1.25%, 0.95% at $N_{\text{train}} = 108$, 270, 541, 812, 1,082; fit $\epsilon \approx 18.5\,N^{-0.43}$, $\gamma = 0.43 \pm 0.11$. **(c)** LDC (988-sample test set). Channel-mean errors 15.6%, 11.3%, 4.4%, 6.4%, 3.3% at $N_{\text{train}} = 320$, 800, 1,600, 2,400, 3,200; fit $\epsilon \approx 440\,N^{-0.58}$, $\gamma = 0.58 \pm 0.23$. Filled circles are per-condition errors; dashed curves are the least-squares fits with the 95% confidence interval on the exponent.

S9.4 Empirical Lipschitz characterisation

To quantify this directly, the empirical Lipschitz constant of the deterministic-mode MIMONet operator was measured on the held-out heat-exchanger test set. For 1,000 random perturbation directions $\boldsymbol{\delta}$ drawn at each of 20 magnitudes $\|\boldsymbol{\delta}\|_2 \in [0.01, 0.50]$ in the combined branch-input space $\mathbb{R}^{102}$, the ratio $\|\mathcal{G}_\theta(\boldsymbol{u}+\boldsymbol{\delta}) - \mathcal{G}_\theta(\boldsymbol{u})\|_2/\|\boldsymbol{\delta}\|_2$ was computed per output channel and aggregated to its 99th percentile. The combined boundary vector was split back into the two encoders so that perturbations propagate through both functional (q_{wall}) and scalar ($T_{\text{in}}, u_{\text{in}}$) input modalities. Inputs $\boldsymbol{u}$ were sampled uniformly from the 310 test cases.

The empirical Lipschitz characterisation is shown in Figure S7. The 99th-percentile bounds are $L_p \approx 0.88$ (pressure), $L_{u_z} \approx 0.37$, $L_{u_y} \approx 0.64$, and $L_{u_x} \approx 1.02$, yielding the global 99th-percentile proxy $L \approx 1.02$ reported. This is a measured percentile and not a bound: by construction one per cent of the sampled ratios exceed it. The ratios are essentially flat across the entire $\|\boldsymbol{\delta}\|_2$ range, indicating that the operator response is locally linear in this neighbourhood of the test manifold; this is the expected behaviour of a smooth approximated operator. The connection to the relative noise-robustness results in the main text is as follows: a bounded L enforces a worst-case *absolute* scaled-output deviation of $\leq L\,\|\boldsymbol{\eta}\|_2$ for an input perturbation $\boldsymbol{\eta}$. The two studies parametrise the perturbation differently and the conversion must be stated: the sweep above is indexed by the vector norm $\|\boldsymbol{\delta}\|_2 \in [0.01, 0.50]$, whereas the Gaussian study of Section S9.6 is indexed by a *per-component* standard deviation σ_{noise}, so that $\|\boldsymbol{\eta}\|_2 \approx \sigma_{\text{noise}}\sqrt{102} \approx 5.05$ at $\sigma_{\text{noise}} = 0.5$. The measured envelope therefore covers the Gaussian sweep only up to $\sigma_{\text{noise}} \approx 0.05$, that is, its first non-zero level, and the linearity observed here cannot be extrapolated to the larger noise levels; that regime is characterised by direct measurement in Section S9.6 instead. Within the range actually probed, the deterministic-pass *relative* RMSE inflation is much smaller than the worst-case envelope; MC-Dropout averaging further reduces it to the sub-3% level reported in the main text by marginalising over weight-space realisations.

S9.5 Structured (non-Gaussian) sensor-noise robustness

The Gaussian noise model used in the main text isolates the operator's response to isotropic, zero-mean perturbations and serves as the simplest analytical proxy for sensor degradation. Real sensor failures in nuclear environments rarely follow this idealisation: instead, sensors exhibit systematic drift (radiation-induced bias), temporally correlated noise (thermal-electronic coupling between channels), and intermittent dropout (radiation-induced single-event upsets or temporary obstruction). To examine MIMONet's robustness beyond simulated smooth-noise data, three structured-noise models were applied to the held-out heat-exchanger test set, each evaluated against the MC-Dropout ensemble ($N = 20$) baseline.

Sensor drift. A spatially uniform additive bias was applied to all branch inputs, $\tilde{u} = u + d \cdot \mathbf{1}$, with $d \in \{0, 0.02, 0.04, 0.06, 0.08, 0.10\}$ corresponding to up to 10% of the normalised input range. This emulates a slow drift in calibration shared across the wall heat-flux profile $q(z)$ and the inlet scalars $(T_{\text{in}}, u_{\text{in}})$.

Correlated (AR(1)) noise. Temporally correlated perturbations were generated via a first-order autoregressive process $\varepsilon_t = \rho\,\varepsilon_{t-1} + \sigma_{\text{AR}}\,\xi_t$ with $\rho = 0.8$, $\sigma_{\text{AR}} = 0.1$, and $\xi_t \sim \mathcal{N}(0, 1)$. Ten independent realisations were averaged. The strong correlation makes this model substantially harder to suppress than i.i.d. Gaussian noise because successive sensor readings carry similar systematic error.

Sensor dropout. A fraction $p_{\text{drop}} = 10\%$ of input channels (selected uniformly per sample) was zeroed, modelling intermittent failure of individual sensor lines.
The ΔRMSE results are shown in the main text; the measured values are tabulated in Table S18 and the full sweeps are plotted in Figure S8.

Interpretation. All three velocity channels remain below 2% inflation under every structured-noise model, so that under every structured-noise model tested the velocity error stays within a small fraction of its clean value. We avoid the term "operationally robust" here because no application-specific tolerance has been defined against which robustness could be judged; what is established is the measured inflation, not its acceptability for a given deployment. Pressure exhibits a larger 11–13% sensitivity to systematic drift and dropout. This channel-dependent behaviour is physically expected: pressure is a globally coupled elliptic field whose interior reconstruction depends on the full boundary-input vector, so systematic bias and missing

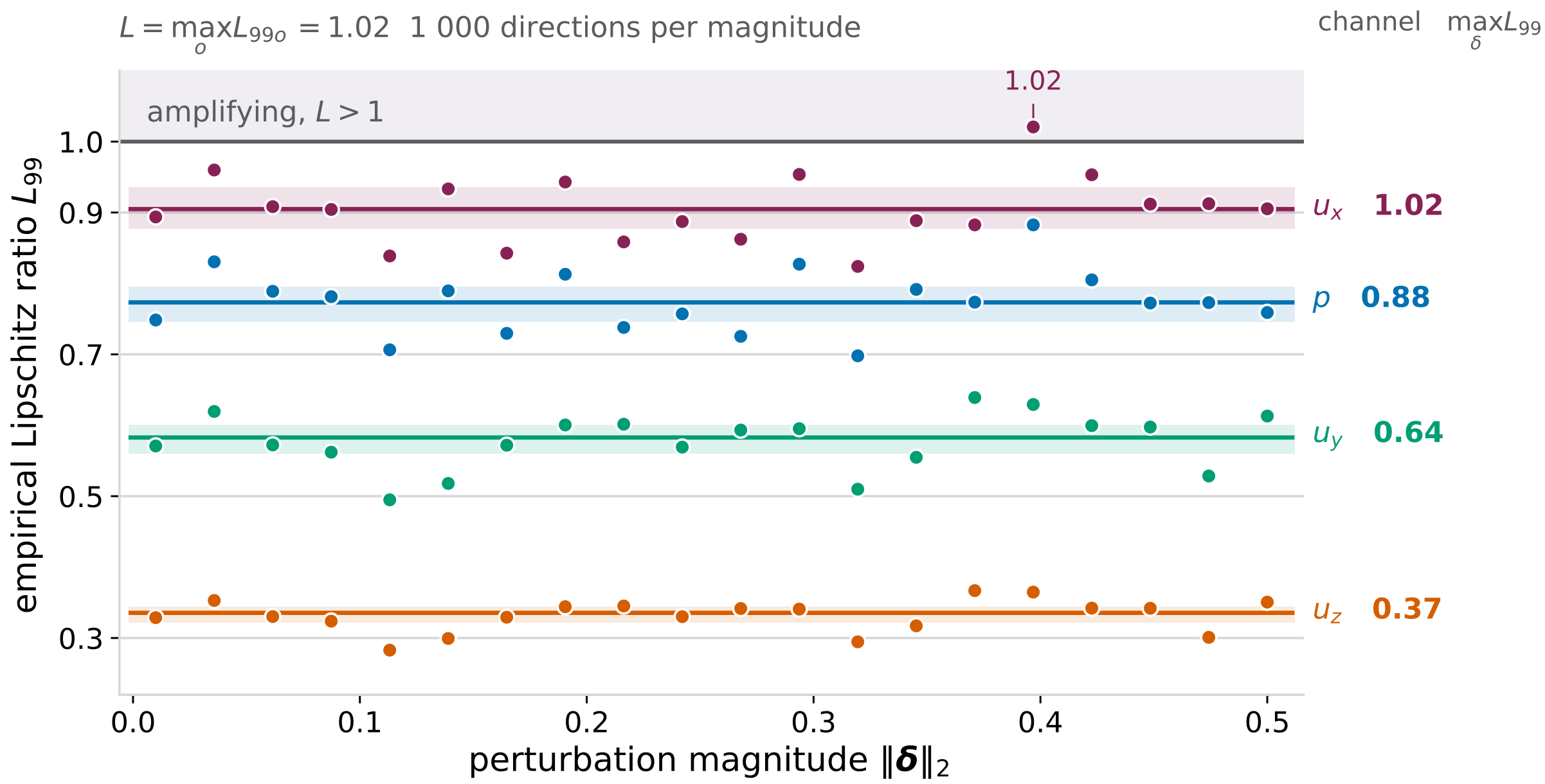

Figure S7: **Empirical Lipschitz characterisation on the heat-exchanger test set.** Empirical 99th-percentile Lipschitz ratio $L_{99} = \|\mathcal{G}(\boldsymbol{u}+\boldsymbol{\delta}) - \mathcal{G}(\boldsymbol{u})\|_2/\|\boldsymbol{\delta}\|_2$ of MIMONet on the heat-exchanger test set, against perturbation magnitude $\|\boldsymbol{\delta}\|_2$, for the four output channels $\{p, u_z, u_y, u_x\}$. Each marker aggregates 1,000 random perturbation directions at that magnitude; the 20 magnitudes are independent measurements and are therefore not joined. For each channel the horizontal line is the median over the 20 magnitudes and the band the interquartile range, so a narrow band indicates a ratio that does not drift with $\|\boldsymbol{\delta}\|_2$. The right-hand labels give $\max_\delta L_{99}$ per channel: 0.88 (p), 0.37 (u_z), 0.64 (u_y) and 1.02 (u_x), the last of which sets the global proxy $L = \max_o L_{99,o} = 1.02$. The shaded region above the solid line is where the operator would amplify a perturbation; exactly one of the 80 measurements enters it, the u_x value at $\|\boldsymbol{\delta}\|_2 = 0.40$, and it does so by two per cent. The flatness across a fiftyfold range of $\|\boldsymbol{\delta}\|_2$ is the behaviour expected of a smooth operator that is locally linear in this neighbourhood of the test manifold.

Table S18: **Structured-noise inflation, tabulated.** ΔRMSE (%) of MC-Dropout MIMONet under structured noise on the heat-exchanger test set, relative to the $\sigma = 0$ baseline. The drift row is the worst value over the six sweep levels rather than the value at $d = 0.10$: the two coincide for p and u_y, whereas the u_z maximum occurs at $d = 0.08$ (the $d = 0.10$ value is 0.48) and the u_x maximum at $d = 0.02$, the error at $d = 0.10$ being 0.35 below baseline. Figure S8 shows every level. Computed channelwise on 310 test cases with 20 stochastic forward passes per evaluation.

**Noise model**	$p(z, y)$	u_z	u_y	u_x
Sensor drift, worst over $d \in [0, 0.10]$	11.36	0.52	1.75	0.03
AR(1), $\rho = 0.8$, mean of 10 realisations	2.28	0.24	0.48	0.25
Random sensor dropout, $p_{\text{drop}} = 10\%$	13.02	0.35	0.67	1.18

channels propagate through the entire field rather than averaging out. By contrast, velocity components are governed by local advective–diffusive transport and are correspondingly less sensitive to coordinated bias. The AR(1) result is the most encouraging: even with strong $\rho = 0.8$ temporal correlation that mimics realistic sensor coupling, ΔRMSE stays below 3% on every channel. The MC-Dropout averaging provides the noise-suppression mechanism by marginalising over weight-space realisations of the operator manifold, an effect that becomes especially valuable for the smoother (correlated) noise spectra encountered in deployed reactor instrumentation. Taken together, these results probe non-idealised sensor noise, which the isotropic

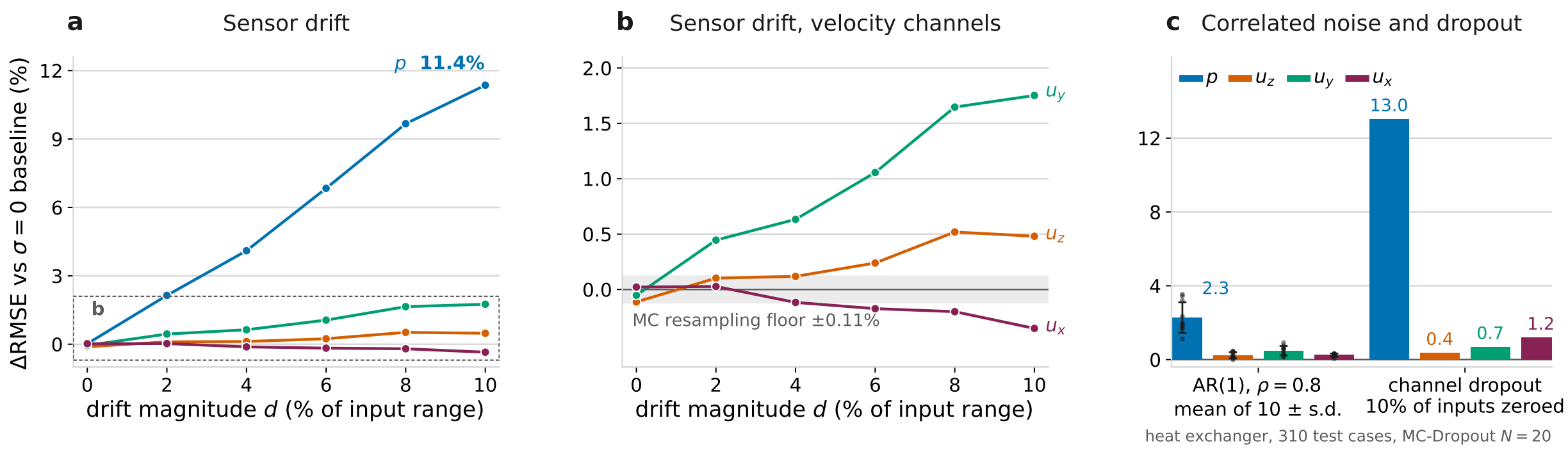

Figure S8: **Structured (non-Gaussian) sensor-noise robustness.** Relative RMSE inflation ΔRMSE (%) of the MC-Dropout MIMONet ($N = 20$) relative to the $\sigma = 0$ baseline, on the 310-case heat-exchanger test set, for the four output channels $\{p, u_z, u_y, u_x\}$. **a**, Uniform additive sensor drift, swept over $d \in \{0, 0.02, \ldots, 0.10\}$ of the normalised input range. The pressure response is close to linear in d, reaching 11.4% at $d = 10\%$, which is the behaviour expected of a globally coupled elliptic field whose interior reconstruction depends on the full boundary-input vector: a coordinated bias does not average out. **b**, The same sweep on the velocity window marked by the dashed rectangle in **a**. All three velocity channels stay below 2% over the entire sweep. The shaded band is the ±0.11% Monte-Carlo resampling floor measured at $d = 0$ from the independent dropout draws of the baseline and the $d = 0$ evaluation, and values inside it are not distinguishable from no effect. u_z and u_y rise slightly but monotonically out of it; u_x moves in the opposite direction, falling to −0.35% at $d = 0.10$, about three times the floor. A small reduction in error under a coordinated input bias is not a result we can explain from the architecture, and we report it as measured rather than interpret it. **c**, AR(1) correlated noise ($\rho = 0.8$, $\sigma_{\mathrm{AR}} = 0.1$) and random channel dropout (10% of input channels zeroed per sample). Bars are means, dots the ten individual AR(1) realisations and whiskers their standard deviation; the AR(1) spread is appreciable relative to the mean, $2.28 \pm 0.79\%$ for pressure, so single realisations range from 1.12% to 3.54%. Every channel *mean* stays below 3% under AR(1) despite the strong temporal correlation, though the largest single pressure realisation reaches 3.54%, while dropout reproduces the pressure sensitivity seen under drift (13.0%). Taking the worst case over all levels of all three models, the velocity channels remain below 2% and pressure between 11% and 13%.

Gaussian model does not given that ground-truth interior fields cannot be measured in operating reactors.

S9.6 Gaussian sensor-noise inflation, tabulated

The main text quotes channel-mean RMSE inflation at $\sigma_{\mathrm{noise}} = 0.5$ for four operator architectures. The complete per-channel numbers are given in Table S19, together with the protocol.

Protocol. Isotropic Gaussian noise $\eta \sim \mathcal{N}(\mathbf{0}, \sigma^2_{\mathrm{noise}}\mathbf{I})$ is added to the scaled branch inputs of the heat-exchanger case, at eleven levels $\sigma_{\mathrm{noise}} \in \{0, 0.05, \ldots, 0.50\}$ in normalised input units, and the test-set RMSE is recomputed per output channel on all 310 held-out cases. Every architecture receives the identical perturbed inputs. The reported quantity is the relative inflation $\Delta\mathrm{RMSE} = [\mathrm{RMSE}(\sigma) - \mathrm{RMSE}(0)]/\mathrm{RMSE}(0)$, so each model is measured against its own clean baseline and the comparison is of noise sensitivity rather than of accuracy. The MC-Dropout entry averages $N = 20$ stochastic passes at every noise level; the deterministic entry is a single pass through the same trained weights with dropout disabled, so the two rows differ in inference mode and not in training.

Two entries need reading with care, and the clean-input block is included so that they can be. KCN shows almost no inflation, but it starts from the worst clean RMSE of the five on pressure, and is mid-pack on the three velocity channels; its neighbour graph and edge weights are constructed once from the training-mesh coordinates and applied as a fixed operator at inference, so most of the noise pathway is short-circuited by a structure that is not responding to the input in the first place. Low sensitivity of that kind is not a virtue. The MC-Dropout and deterministic rows share their weights and differ only in inference mode, so their

Table S19: **Gaussian sensor-noise sensitivity across operator architectures.** Clean-input RMSE and relative RMSE inflation at $\sigma_{\text{noise}} = 0.5$, per output channel, on the 310-case heat-exchanger test set. Inflation is measured against each model's own $\sigma = 0$ baseline, which is given in the upper block so that the two can be read together: a small inflation on a large baseline is not evidence of accuracy.

Model	p	u_z	u_y	u_x	Mean
RMSE at $\sigma_{noise} = 0$ *(scaled units)*					
MIMONet, deterministic	0.0245	0.0358	0.0604	0.1302	—
MIMONet, MC-Dropout (N=20)	0.0245	0.0354	0.0557	0.1117	—
GeoFNO	0.0152	0.0193	0.0231	0.0313	—
NOMAD	0.0445	0.0585	0.0672	0.0935	—
KCN	0.0585	0.0499	0.0670	0.1054	—
ΔRMSE *(%) at* $\sigma_{noise} = 0.5$					
MIMONet, deterministic	42.86	5.31	6.13	2.53	14.21
MIMONet, MC-Dropout (N=20)	5.71	1.13	2.33	0.63	2.45
GeoFNO	555.92	227.46	234.20	258.15	318.93
NOMAD	67.19	37.61	38.10	111.34	63.56
KCN	0.85	0.40	0.60	3.98	1.46

comparison isolates the effect of averaging over dropout masks; the MC-Dropout variant is reported in the main text as an uncertainty-aware mode of the same model rather than as a capacity-matched single-pass competitor.

S9.7 Local parameter extrapolation, tabulated

The main text reports out-of-distribution behaviour on the heat-exchanger case. The per-channel values are given in Table S20.

Protocol. Fifty additional ANSYS Fluent simulations were generated with the inlet velocity, inlet temperature and wall heat-flux amplitude placed approximately 15% outside their training ranges, twenty-five below the lower boundary and twenty-five above the upper. Geometry, solver configuration, convergence criteria, data processing and the target plane at $x = 0.789$ are unchanged from the in-distribution dataset, and the trained models are evaluated on these cases without retraining or fine-tuning.

Table S20: **MIMONet under local parameter extrapolation.** Relative ℓ_2 error (%) per channel on the in-distribution test set and on the out-of-distribution cases, split by whether the operating parameters lie below or above the training range. The final column is the ratio of the pooled out-of-distribution error to the in-distribution error.

Condition	p	u_z	u_y	u_x	Channel mean
In-distribution ($n = 310$)	0.82	1.45	1.02	0.52	0.95
OOD, below range ($n = 25$)	2.04	2.81	2.60	1.23	2.17
OOD, above range ($n = 25$)	0.92	1.92	1.63	0.88	1.34
OOD, pooled ($n = 50$)	1.48	2.37	2.11	1.05	1.75
OOD ÷ in-distribution	1.81	1.63	2.07	2.02	1.84

The degradation is graded rather than abrupt: a 15% excursion in the operating parameters costs a factor of 1.84 in channel-mean error, and the worst per-channel factor is 2.07. The asymmetry between the two directions is consistent across channels, extrapolation below the training range costing roughly 1.6× more than extrapolation above it, which is expected because the lower boundary approaches the laminar-transitional regime where the field structure changes more rapidly with the parameters. No claim of

extrapolation beyond this margin is made; the experiment establishes that the operator degrades continuously over a 15% excursion, not that it remains valid at larger ones.

S10 Computational complexity

The property at issue is amortisation. A virtual sensor is useful inside a monitoring loop only if the cost of producing a field does not scale with the effort that went into learning it, and the operator formulation moves that cost to an offline training stage. This section states the forward cost, compares it with the alternatives, and is explicit about which rows of that comparison were measured here and which are reference figures taken from the literature.

S10.1 Forward-pass complexity

The requirement at issue here is amortisation: a virtual sensor must answer a new operating condition within a monitoring loop, without solving a governing system online and without refitting. The costs below are what that amortisation buys, and they are the analytic counterpart of the measured latencies in Section S11.

For batch size B, query points P, latent dimension I, and m output channels:

- **Branch networks:** Each branch with L layers of width W: $\mathcal{O}(B \cdot L \cdot W^2)$ per branch.
- **Multiplicative fusion:** $\mathcal{O}(B \cdot I \cdot n)$ where n is number of input modalities.
- **Trunk network:** $\mathcal{O}(P \cdot L' \cdot W'^2)$ for trunk with L' layers of width W'.
- **Einstein summation:** $\mathcal{O}(B \cdot P \cdot I \cdot m)$.

Total: $\mathcal{O}(B \cdot P \cdot I \cdot m)$ dominates for large P, yielding linear scaling in the number of query points.

S10.2 Comparison with competing methods

The asymptotic costs of the methods compared in this work are collected in Table S21.

Table S21: **Computational complexity comparison for operator learning methods.** P is the number of query coordinates, I the branch–trunk latent width and n the number of conditioning observations. The finite-element row is an order-of-magnitude figure taken from the literature and not a measurement reported here.

Method	Training	Inference
Finite Element Method	$\mathcal{O}(P^{1.5-2})$	$\mathcal{O}(P^{1.5-2})$ per solve
Gaussian Process	$\mathcal{O}(n^3)$	$\mathcal{O}(Pn)$
Fourier Neural Operator	$\mathcal{O}(P \log P)$	$\mathcal{O}(P \log P)$ per mode
**MIMONet (ours)**	$\mathcal{O}(P \cdot I)$	$\mathcal{O}(P \cdot I)$

Here P is the number of query coordinates and n the number of conditioning observations; the two are distinct, and conflating them is what produces the common claim that operators are asymptotically cheaper than Gaussian processes at query time. They are not: both are linear in P. The difference is where the cubic term sits, as set out in Section S3. No solver timing is measured in this work, so the comparison with the finite-element row is an order-of-magnitude statement taken from the literature rather than a measurement reported here.

S10.3 Memory footprint

Training: $\mathcal{O}(N_{\text{params}} + B \cdot P \cdot m)$ for model parameters and batch activations. **Inference:** $\mathcal{O}(N_{\text{params}} + P \cdot I)$, independent of training set size. Measured peak inference VRAM is 2.4–3.5 GB on the data-centre GPUs benchmarked here (Table S22); no embedded or edge platform was tested, and we make no claim about one.

S11 Real-time inference benchmarking across GPU architectures

The efficiency comparison in the main text is made on a single GPU. This section reports the same workload across four hardware generations, so that the architectural conclusions can be separated from the machine they were measured on.

Each metric is the mean over $N = 1{,}000$ independent inferences; per-run spreads were not retained by the benchmarking harness and are therefore not tabulated. Latency ($\bar{t}$) is the average forward-pass duration per full-field reconstruction, throughput (TPS) is the reciprocal of latency, power ($\bar{P}$) is the mean instantaneous GPU power, and energy ($\bar{E}$) is the average per-inference energy cost computed as $\bar{E} = \bar{P}\,\bar{t}$. VRAM denotes the peak GPU memory usage during inference. All tests were performed using identical trained MIMONet weights with batch size $B = 1$ and trunk query size $P = 3{,}977$.

Table S22: **MIMONet single-inference hardware-portability measurements.** HeatExchanger case (B=1, P=3,977, N=1,000 timed forward passes). Latencies are wall-clock means over 1,000 passes after 10 warmups; power is sampled via NVML and averaged across the timed window; energy = power × latency. The PWR-subchannel and LDC cases give the same qualitative ordering and are reported in Supplementary Table S23.

**GPU**	**Architecture**	$\bar{t}$ (ms)	TPS (samples s^{-1})	$\bar{P}$ (W)	$\bar{E}$ (mJ)	VRAM (GB)
NVIDIA A40	Ampere	0.84	1,190	72.6	61.2	2.38
NVIDIA A100 (40 GB)	Ampere	0.81	1,230	124.9	100.4	2.54
NVIDIA H200	Hopper	**0.35**	**2,860**	129.2	**45.6**	3.00
NVIDIA GH200 (120 GB)	Grace Hopper	1.17	855	156.7	182.6	3.45

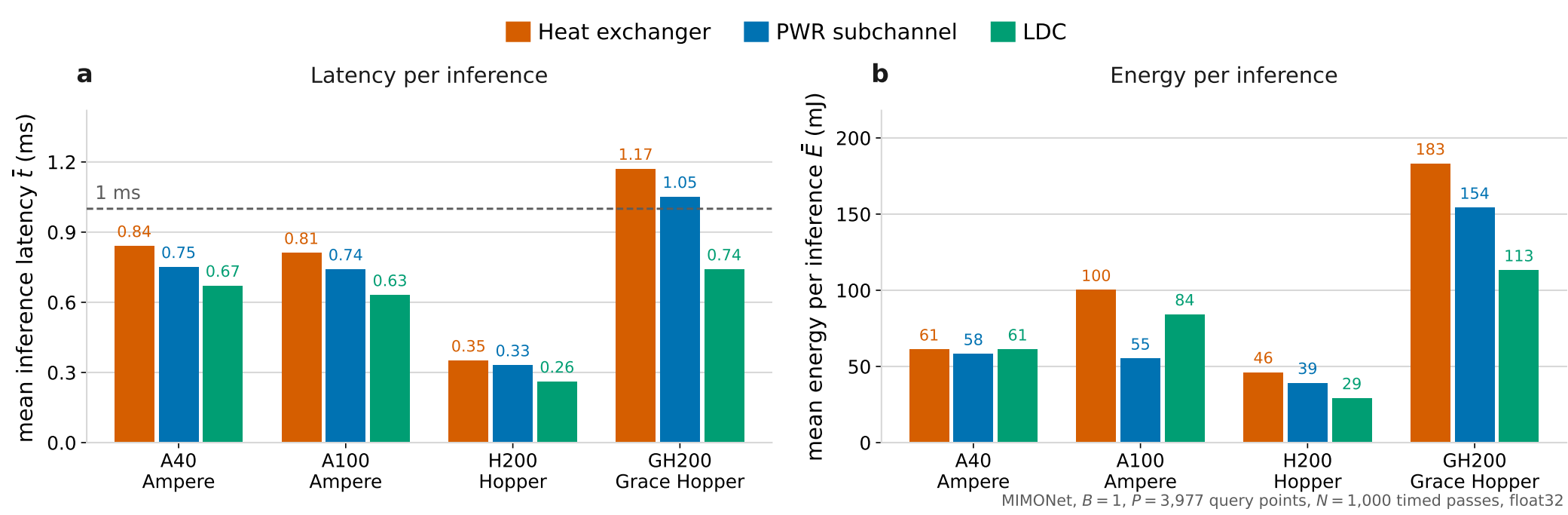

Figure S9: **Hardware portability of MIMONet across NVIDIA data-centre GPUs.** Measurements on the A40, A100, H200 and GH200 under identical workload conditions (B=1, P=3,977 query points, N=1,000 timed forward passes, float32), reported separately for each of the three case studies. **a**, Mean inference latency $\bar{t}$; the dashed line marks 1 ms. **b**, Mean energy per inference $\bar{E} = \bar{P}\,\bar{t}$, with $\bar{P}$ the mean NVML board power over the timed window. Three of the four GPU generations achieve sub-millisecond single-inference latency on every case, and the slowest measurement anywhere is 1.17 ms on the GH200. The Hopper-generation H200 is both fastest and most energy-efficient ($\bar{t} \leq 0.35$ ms, $\bar{E} \leq 46$ mJ across the three cases). The GH200 yields larger latency and energy than the H200 at this workload because Grace Hopper's coherent CPU–GPU interconnect is optimised for larger working sets and does not improve the launch envelope of a single B=1 inference. The architectural-efficiency comparison across operator families on a single GPU is given in the main text; this figure isolates the hardware-generation contribution under a fixed model. All values are the MIMONet column of Supplementary Table S23.

The measurements are given in Table S22 and plotted in Figure S9. Single-inference MIMONet latency is sub-millisecond on three of the four GPUs tested on every case study, and the slowest measurement anywhere is 1.17 ms on the GH200. The lowest latency and energy are obtained on the Hopper-generation H200 (0.26–0.35 ms, 29–46 mJ across the three cases), followed by A40 (0.67–0.84 ms, 58–61 mJ) and A100 (0.63–0.81 ms, 55–100 mJ). The Grace Hopper GH200 is the outlier at this workload (0.74–1.17 ms, 113–183 mJ):

the coherent CPU–GPU interconnect adds a fixed launch overhead that dominates at the sub-millisecond scale of a single B=1 inference, so although peak throughput of GH200 is high, its single-shot latency is larger than H200's. Normalised per-point energy is therefore lowest on H200 ($\bar{E}/P \approx 11.5\,\mu$J/point on HX) and highest on GH200 ($\approx 45.9\,\mu$J/point on HX). The architectural ranking established in the main text is preserved across all four GPUs (Supplementary Table S23), confirming that the cross-model conclusions are properties of the architecture, not of the specific GPU on which the comparison is reported. The hardware-portability panel above is therefore retained as a separate characterisation rather than a model-efficiency claim.

Table S23: **Full architecture×GPU×case efficiency matrix.** Each cell shows latency / energy per inference (ms / mJ) under the identical workload (B=1, $P=$3,977, $N=$1,000 timed passes, float32). Bold marks the lowest entry within each (case, metric, GPU) group of four architectures. The cross-architecture ordering established in the main text is preserved on every GPU tested, confirming the conclusions are architectural rather than hardware-specific.

**Case**	**GPU**	**MIMONet**	**GeoFNO**	**NOMAD**	**KCN**
Heat exchanger	A40	0.84 / 61	5.36 / 611	**0.50** / **60**	3.11 / 270
	A100	0.81 / 100	5.46 / 414	**0.50** / **37**	3.15 / 207
	H200	0.35 / 46	3.08 / 460	**0.23** / **36**	1.75 / 216
	GH200	1.17 / 183	11.52 / 1727	**0.80** / **121**	4.28 / 611
PWR subchannel	A40	0.75 / 58	5.42 / 522	**0.52** / **51**	3.12 / 259
	A100	0.74 / 55	5.46 / 367	**0.50** / **33**	3.14 / 195
	H200	0.33 / 39	3.03 / 402	**0.22** / **31**	1.73 / 206
	GH200	1.05 / 154	7.13 / 1046	**0.80** / **117**	4.03 / 569
LDC	A40	**0.67** / **61**	5.22 / 634	1.47 / 340	2.97 / 284
	A100	**0.63** / **84**	5.27 / 437	1.42 / 336	3.01 / 201
	H200	**0.26** / **29**	2.91 / 453	0.67 / 159	1.62 / 208
	GH200	**0.74** / **113**	6.82 / 1066	1.12 / 251	3.90 / 561

Code Availability

Reproducible code and data are available at `https://doi.org/10.5281/zenodo.21986357`; a permanent DOI will be assigned upon publication.

References

[1] Jaume Ramon, Llorenç Lledó, Núria Pérez-Zanón, Albert Soret, and Francisco J. Doblas-Reyes. The Tall Tower Dataset: a unique initiative to boost wind energy research. *Earth System Science Data*, 12(1):429–439, 2020.

[2] Lu Lu, Pengzhan Jin, Guofei Pang, Zhongqiang Zhang, and George Em Karniadakis. Learning nonlinear operators via DeepONet based on the universal approximation theorem of operators. *Nature machine intelligence*, 3(3):218–229, 2021.

[3] Tianping Chen and Hong Chen. Universal approximation to nonlinear operators by neural networks with arbitrary activation functions and its application to dynamical systems. *IEEE transactions on neural networks*, 6(4):911–917, 1995.

[4] Jacob Seidman, Georgios Kissas, Paris Perdikaris, and George J Pappas. NOMAD: Nonlinear manifold decoders for operator learning. *Advances in Neural Information Processing Systems*, 35:5601–5613, 2022.

[5] Gabriel Appleby, Linfeng Liu, and Li-Ping Liu. Kriging convolutional networks. In *Proceedings of the AAAI conference on artificial intelligence*, volume 34, pages 3187–3194, 2020.

[6] Zongyi Li, Daniel Zhengyu Huang, Burigede Liu, and Anima Anandkumar. Fourier neural operator with learned deformations for pdes on general geometries. *Journal of Machine Learning Research*, 24(388):1–26, 2023.

[7] Vladimir Vovk, Alex Gammerman, and Glenn Shafer. *Algorithmic Learning in a Random World*. Springer, 2005.

[8] Jing Lei, Max G'Sell, Alessandro Rinaldo, Ryan J. Tibshirani, and Larry Wasserman. Distribution-free predictive inference for regression. *Journal of the American Statistical Association*, 113(523):1094–1111, 2018.

[9] Nikola Kovachki, Zongyi Li, Burigede Liu, Kamyar Azizzadenesheli, Kaushik Bhattacharya, Andrew Stuart, and Anima Anandkumar. Neural operator: Learning maps between function spaces with applications to pdes. *Journal of Machine Learning Research*, 24(89):1–97, 2023.

[10] Tim De Ryck and Siddhartha Mishra. Generic bounds on the approximation error for physics-informed (and) operator learning. *Advances in Neural Information Processing Systems*, 35:10945–10958, 2022.
