# Supplementary material for: Virtual sensing to enable real-time monitoring of inaccessible locations & unmeasurable parameters
Source: Nat Commun. 2026 Sep 19;17:10976. doi: 10.1038/s41467-026-77463-7 (PMC13683354; doi:10.1038/s41467-026-77463-7)
Supplement: Supplementary file 2 [file 41467_2026_77463_MOESM2_ESM.pdf]

nature portfolio

Peer Review File

Virtual Sensing to Enable Real-Time Monitoring of Inaccessible Locations & Unmeasurable Parameters

Corresponding Author: Dr Syed Bahauddin Alam

This file contains all reviewer reports in order by version, followed by all author rebuttals in order by version.

Attachments originally included by the reviewers as part of their assessment can be found at the end of this file.

Version 1:

Reviewer comments:

Reviewer #1

(Remarks to the Author)
Noteworthy results?
-achieves consistently low errors (~0.5–5%) while also reconstructing full interior fields from sparse or boundary-only data which is a bit harder than point prediction.
- outperforms established neural operator baselines for eg GeoFNO, NOMAD, KCN by large margins across all three test problems.

Significant to the field and related fields?
-Yes, i think it advances neural operators from surrogate modeling to deployable virtual sensing, especially in safety-critical systems.
-The approach is broadly applicable beyond nuclear systems (e.g., aerospace, chemical reactors, batteries).
-also it directly addresses a known gap in SciML: multi-field, interior inference in inaccessible domains.

contribution:
even though built on known components, the architecture, framing and validation together represent a novel contribution.

Does the work support the conclusions and claims?
I can see that core claims about accuracy, scalability and robustness are well supported by quantitative results and comparisons.

Are there flaws in data analysis, interpretation, or conclusions?
- No major flaws in analysis or interpretation, also metrics and comparisons are appropriate.

Is the methodology sound and up to field standards?
Yes, i think the neural operator formulation, training setup, and evaluation protocol are technically sound. Baseline models are also appropriate and fairly compared.

Is there enough detail to reproduce the work?
reproducibility is good though a public code or dataset would strengthen it further.

(Remarks on code availability)
Yes. The authors provide accompanying code at https://github.com/kkazuma19/MIMONet and a README file is available that clearly documents the installation requirements, dependencies, and steps needed to run the experiments

Reviewer #2

(Remarks to the Author)
Please see the attached pdf file.

(Remarks on code availability)

Reviewer #3

(Remarks to the Author)
The manuscript proposes a neural network model for real-time virtual sensing, with special focus on thermal and fluid dynamics. I find the paper contributions to be narrow for the Nature Communications readership, and therefore I do not recommend the paper for publication. I discuss the reasons in detail below:

1) It is not clear what fundamental challenge is solved by the proposed method can address that cannot already be handled by existing approaches. There is a wide variety of virtual sensing methods in the literature, from classical ones like Kalman filters, MHE, and basic machine learning tools like SVMs, to physics-informed neural networks or identification models like SINDy. Although some of these methods are acknowledged in the Introduction, the authors do not benchmark the performance of their method against them, neither in computational time nor in performance analysis. For instance, the noise robustness assessment is made exclusively within the context of the proposed method (and its variations). For the considered case studies, it is not clear why other physics-based ML models could not be readily deployed to solve these problems.

2) I find the claims/analysis on energy efficiency to be even more lacking, given that the energy comparison is made across GPUs rather than across model architectures. Thus, the authors are actually evaluating the neural network inference efficiency of a GPU rather than the efficiency of their model relative to others.

3) Given the overemphasized motivation of the paper on the need for *real-time* virtual sensing methods for critical applications, I find the lack of model validation on real-world data to be a critical limitation of the work. The authors go through the effort of measuring the inference time and energy consumption of their model computations, but none of this matters if the model cannot be validated beyond simulated (highly smooth) data.

4) Moreover, the paper presentation is exaggerated. The introduction is unnecessarily long, with repetitive arguments or in-depth discussion of the literature that is not directly related to the main contribution of the paper. The methodological details on the simulations are also distracting from the main results in the paper and could be moved to the methods or supplement.

(Remarks on code availability)

Version 3:

Reviewer comments:

Reviewer #2

(Remarks to the Author)
[This is contained in the uploaded pdf file.]

Review of Nature Communications manuscript NCOMMS-25-12559C-Z “Virtual Sensing to Enable Real-Time Monitoring of Inaccessible Locations & Unmeasurable Parameters” by Kazuma Kobayashi, Farid Ahmed, Jaewan Park, Subhankar Sarkar, Seid Koric, Souvik Chakraborty and Syed Bahauddin Alam

The paper describes an approach to using machine learning encoders to ‘learn’ the full solution space of physical systems described by constituent relations — PDEs, ODEs, etc – using ‘data’ consisting of vector input functions, $u \in U$,and simulation-based grid samples of vector internal ‘fields,’ $s \in S$. The fitting is performed on (u(i),s(i)) data pairs using least-squares fitting over parameter θ. The operator $G : U \rightarrow S$ is presumed time-invariant, although the functions uand smay be functions of time. Once this operator is fitted, then the “sparse boundary. measurements” ucan generate associated “physically inaccessible” internal state estimates s(r) at any interior point r. The learnt encoders have low computational complexity and so the rationale for this procedure lies in the capacity of running these learnt forward operators in close to real time, while the simulation is time- and computation-intensive. The formulation is described by lines 315 through 318. There is a set of three examples: a Navier-Stokes flow, a pressurized water reactor, and heat exchanger. More convincingly and impressively, there are also three examples with real-world physically sensed data with appropriate experimental excitation and complexity: a fuel cell, a wind turbine tower, and ocean-state reconstruction.

I thank the authors for their care in addressing my earlier comments, the clarity and thoroughness of the analysis and experimentation. Originally, I had significant difficulty appreciating the science behind this paper and its claims. My core issues with the approach were that it is essentially an inductive procedure to fit the operator G. The philosophy of science has been grappling for centuries with inductive reasoning [1, 2] and with the connection between data and system complexity [3, 4]. In a sense, the inductive question is bypassed early on in the paper by presuming precise knowledge of the constituent equations, whose possible precision is another issue for the philosophy of science [5, 6]. [I am not recommending that the authors cite any of these references here. This is just to highlight the problems.]

For Example 1, the input function for data generation is described in lines 386-390. The learning is described in lines 400-421. The input test set is isomorphic to the excitation set, which is central to the issue that I had with the paper and its links to induction; the efficacy requires the future inputs to be very close to past inputs used in the training. Indeed, since machine learning is an inductive pursuit, this is a systemic problem. The two other examples share this issue.

However, I now appreciate that the authors perceive and address these issues head on in the subsequent analysis and in the application to real-world experimental data. Further, in reading their critiques, I am impressed with their careful quantification of the issues and their care in developing claims. As a meaningful path forward to the development of these techniques and their application, I am very pleased to recommend the paper for publication.

I concur with Reviewer 3 that the paper is rather wordy and, at times, distractingly detailed. Some reorganization would be welcome to focus on the major claims, as opposed to the implementation issues, which could be relegated to appendices but are important for reproducibility. Rereading the revised paper was necessary to dispel my earlier negative assessment but is also a sign of the difficulty to separating the essential ideas from the details.

My remaining minor quibble and tie back to Kalman filtering and to my earlier remarks, it would appear that the role of time is a bit obscure and could be given some more space. The methods rely on mapping input signal functions state estimates, and this would appear to be performed using the entire input function over time, as opposed to the piecewise input function up to the current time. So, the comparison is really with what is called fixed-interval smoothing in the control systems Kalman filtering area [7]. I should expect that the "real-time" computational aspects are when compared to simulation time estimates rather than contemporaneous state estimate of Kalman. You might want to comment so that people like me are less confused

References

[1] J. Vickers, "The Problem of Induction," in The Stanford Encyclopedia of Philosophy, E. N. Zalta and U. Nodelman, Eds. Metaphysics Research Lab, Stanford University, June 2010. [Online]. Available: https://plato.stanford.edu/entries/induction-problem/#2HumIndJus
[2] L. Henderson, "The Problem of Induction," in The Stanford Encyclopedia of Philosophy, Winter 2024 ed., E. N. Zalta and U. Nodelman, Eds. Metaphysics Research Lab, Stanford University, 2024. [Online]. Available: https://plato.stanford.edu/entries/induction-problem/
[3] R. Solomonoff, "A Formal Theory of Inductive Inference. Part I," Information and Control, vol. 7, pp. 1–22, 1964.
[4] ——, "A Formal Theory of Inductive Inference. Part II," Information and Control, vol. 7, pp. 224–254, 1964.
[5] K. Popper, Conjectures and Refutations: the growth of scientific knowledge. New York, NY: Basic Books, 1962.
[6] G. Box, "Science and statistics," Journal of the American Statistical Association, vol. 356, pp. 791–799, 1976.
[7] B. Anderson and J. Moore, Optimal Filtering. Englewood Cliffs, New Jersey: Prentice-Hall, 1979.

(Remarks on code availability)

Reviewer #3

(Remarks to the Author)
The authors have made a genuine effort to revise the manuscript, improve presentation, and address the concerns in the first round of revision. In particular, the revised text now: 1) clearly distinguishes between the neural net approach for operator learning proposed in the paper and previous methods for state estimation (observers, Kalman filters, SINDy, PINNs); 2) avoids overusage of jargon and abuse of terminology; and 3) introduces new results based on experimental data. I am satisfied to see that the good performance of the method, originally evaluated only on simulated/artificial data, also extends to experimental data.

I believe the paper contains publishable results of broad interest for the Nature Communications audience, and that the underlying examples on simulated/experimental data demonstrate well the applicability of the proposed method.

However, I still believe the paper presentation is subpar for Nature Communications. First, the paper is unnecessarily long. Second, information is often presented in a repetitive way, making the text not concise. Third, many of the methodological details and results are unnecessary to the main point of the paper and could be moved to the supplement. In my opinion, the paper could be presented in <20 pages, rather than 40. Below, I give specific comments to the authors on how I believe this paper could be condensed.

1) Introduction is too long. In my opinion, the Introduction could be condensed to lines 1-46 + 96-103 + a more concise description of 140-170.

Everything else concerns related work on observer/state estimation (lines 47-62), data-driven and machine learning models (lines 63-94), and PINNs (lines 104-139). It is indeed important to emphasize this related literature, as done in Table 1, but such an in-depth discussion is unnecessary for the main text and distracts from the main contribution. Moreover, there is a lot of overlap between this literature review and Sec. 2.1, where the authors again discuss the difference between operator-based approaches and classical state estimation. In fact, these discussions could be combined and made more concise, so that Secs 2.1 and 2.2 are also shorter.

2) The presentation of the results on simulated data (Secs 2.4, 2.5, and 2.6) is also too long. Take for example Problem 3 (Sec 2.6). Lines 517-532 are the problem motivation, lines 533-556 are the model setup to generate the simulated data, lines 557-577 are numerical details on the method implementation, and lines 578-611 are the actual results. Only the problem motivation and results need to be presented in the main text (along with a bit of the model setup), the remaining can be moved to the SM or Methods.

Moreover, in my opinion, Problems 1 and 2 concern very simple case studies, in which, far away from the boundaries, the flow is quite homogeneous and simple, making its prediction simple. Only in Problem 3 we see a flow with non-trivial structure far from the boundaries (which the proposed approach successfully captures). Thus, in my opinion, Problems 1 and 2 can be seen as proof-of-concepts, and hence one or both could be moved to the SM to save space.

3) Sec. 2.7 is nice and interesting, but the results in Secs. 2.8-2.12 also seem supplementary. In my opinion, these results could be moved to the SM and the authors should write a single short section (along with a single figure) summarizing the main findings in Secs 2.8-2.12. The details can be described fully in the SM.

4) Secs 3.1-3.3 are interesting and should be kept in the main text, but the authors should consider following my suggestions on point 2 to also reduce their presentation, focus on the results, and move methodological/data details to the SM.

(Remarks on code availability)

Open Access This Peer Review File is licensed under a Creative Commons Attribution 4.0 International License, which permits use, sharing, adaptation, distribution and reproduction in any medium or format, as long as you give appropriate credit to the original author(s) and the source, provide a link to the Creative Commons license, and indicate if changes were made.
In cases where reviewers are anonymous, credit should be given to 'Anonymous Referee' and the source.
The images or other third party material in this Peer Review File are included in the article’s Creative Commons license, unless indicated otherwise in a credit line to the material. If material is not included in the article’s Creative Commons license and your intended use is not permitted by statutory regulation or exceeds the permitted use, you will need to obtain permission directly from the copyright holder.
To view a copy of this license, visit https://creativecommons.org/licenses/by/4.0/

Point-by-Point Response to Reviewers

VIRTUAL SENSING TO ENABLE REAL-TIME MONITORING OF INACCESSIBLE LOCATIONS & UNMEASURABLE PARAMETERS

EDITOR'S COMMENTS:

We appreciate the points you raise, and are not questioning the validity of your work or its interest to others in the field. However our view remains that, in the absence of a clear demonstration of significant advance in performance on real-world datasets, publication in Nature Communications would be premature.

We thank the Editor for clarifying the central basis of the decision. We agree that a manuscript motivated by real-time virtual sensing for critical applications must demonstrate not only simulation-based feasibility but also a clear and significant performance advance on real-world datasets. The originally submitted version did not provide this evidence sufficiently. The revised manuscript addresses this requirement directly through a new Section 3 reporting three independent real-world experiments.

The three experiments were selected to form an escalating sequence of virtual-sensing difficulty, each instantiating a distinct physical instance of the paper's core claim.

On the proton-exchange-membrane fuel cell (PEMFC) fuel-cell internal-field experiment, MIMONet reconstructs internal current-density and temperature fields from only 32 of 324 grid points, corresponding to 10% sensor coverage, achieving 1.90 ± 0.51% channel-mean relative L^2 error against 11.67% for the best classical baseline (IDW), an advance of +84%. The current-density channel, the primary unmeasurable field, reconstructs to 3.77 ± 1.03% versus 23.10% for IDW. This case is the most direct real-world analogue of the nuclear reactor motivation: the current-density-mapping probe used to generate laboratory ground truth cannot be embedded as an operational sensor in a deployed cell.

On the International Energy Agency (IEA)-Wind tall-tower experiment, consisting of purely in-situ anemometry at seven international tower sites across five climatic zones with no model or reanalysis component, MIMONet reconstructs hub-height wind speed at 80 m from lower-level sensors at 10, 25, and 50 m, achieving 4.71 ± 0.15% relative L^2 error against 27.86% for the best classical baseline, an advance of +83%. The target height is not observed by the lower-level sensor network, and fixed-form engineering extrapolation laws fail under atmospheric stratification. At 100 m, the standard power law reaches 95.92% error while MIMONet remains at 14.70 ± 0.59%, a 6.5-fold lower error corresponding to an 85% error reduction.

On the GLORYS12 North Atlantic ocean-state experiment, a publicly available ocean reanalysis fusing Argo float profiles, satellite altimetry, and sea-surface temperature retrievals, MIMONet jointly reconstructs temperature, salinity, and sea-surface height from two disjoint sparse sensor networks consisting of 400 temperature and 400 salinity locations, achieving 4.22 ± 0.06% channel-mean error against 15.16% for the best classical baseline, an advance of +72%. GLORYS12 is a real-world ocean reanalysis product that carries genuine observational structure from in-situ and satellite measurements.The most decisive result is sea-surface height: SSH carries no direct sensors in the input domain, so classical spatial interpolation cannot operate on

that channel directly. MIMONet recovers SSH at 9.11 ± 0.17% against 37.7% for climatology, a 4.1-fold lower error corresponding to a 76% error reduction. This demonstrates cross-modal recovery of a physically coupled field that classical spatial interpolation or locality-based learned interpolation cannot recover under the stated sparse-sensor protocol when the target channel has no direct observations.

A comprehensive method comparison table in the revised manuscript shows all evaluated method families across the three domains. The advance is quantitative (+72% to +84% across three domains), qualitative (cross-modal SSH recovery from zero direct input sensors), and systematic (consistent gains across an electrochemical energy device, an in-situ wind-energy measurement network, and a real-world North Atlantic ocean-state reconstruction). These results provide the clear demonstration of significant advance on real-world field data requested by the Editor.

RESPONSE TO REVIEWER #1 (R1)

We thank Reviewer #1 for the careful and constructive evaluation. We appreciate the reviewer's assessment that the methodology is sound, the comparisons are appropriate, and the combined formulation, architecture, and validation constitute a meaningful contribution to scientific machine learning and virtual sensing.

1. *Noteworthy results? achieves consistently low errors (~0.5-5%) while also reconstructing full interior fields from sparse or boundary-only data which is a bit harder than point prediction. outperforms established neural operator baselines for e.g., GeoFNO, NOMAD, KCN by large margins across all three test problems.*

Authors' Response: We appreciate this characterization. In contrast to predicting a prescribed scalar or finite target vector, the present task requires reconstruction of multiple physical quantities over the complete query domain from boundary-only or spatially separated inputs. The information asymmetry is substantial, with reconstruction ratios ranging from 47:1 to 156:1 across the three experiments. The cases additionally introduce geometric confinement, multimodal forcing, turbulence, and coupled output fields. We acknowledge this distinction in the revised manuscript. The reconstruction task involves recovering 4,225 to 3,977-node interior fields from inputs of dimension 90 to 102, representing reconstruction ratios from approximately 47:1 to 156:1 across the three experiments; this information asymmetry is why operator-based field reconstruction is categorically harder than pointwise regression.

Revision: The reconstruction information asymmetry is now explicitly framed in the problem-class distinction (Section~2.1), which clarifies why the task is harder than pointwise prediction.

2. *Significant to the field and related fields? Yes, I think it advances neural operators from surrogate modeling to deployable virtual sensing, especially in safety-critical systems. The approach is broadly applicable beyond nuclear systems (e.g., aerospace, chemical reactors, batteries). Also, it directly addresses a known gap in SciML: multi-field, interior inference in inaccessible domains.*

Authors' Response: We agree entirely and have strengthened the Discussion to explicitly enumerate these cross-domain application pathways with specific references to cardiovascular hemodynamics, structural health monitoring, and battery thermal management.

Revision: Discussion paragraph expanded with cross-domain applications.

3. *Contribution: even though built on known components, the architecture, framing and validation together represent a novel contribution. Does the work support the conclusions and claims? I can see that core claims about accuracy, scalability and robustness are well supported by quantitative results and comparisons.*

Authors' Response: We thank the reviewer for this precise and balanced assessment. The contribution lies in combining modality-specific input encoding, multiplicative latent interactions, coordinate-conditioned decoding, and simultaneous prediction of multiple physical

fields within a unified operator-based virtual-sensing formulation. The revised manuscript now states this contribution relative to prior operator architectures without claiming that the individual components are independently unprecedented.

Revision: Introduction reduced by approximately 30%; detailed solver settings, convergence criteria, material-property handling, and extended parameter-generation details moved to the Methods and Supplementary Methods; contribution summary and two-layer validation strategy clarified at the end of the Introduction.

4. *Reproducibility: reproducibility is good though a public code or dataset would strengthen it further.*

Authors' Response: The public GitHub repository (linked in the Code Availability section) hosts the complete artefacts for reproducing every result reported in this work: the full MIMONet source code, training and evaluation scripts, experiment configurations, all three preprocessed simulation datasets (lid-driven cavity, PWR subchannel, and heat exchanger), and the trained model checkpoints for both the deterministic and the MC-Dropout heat-exchanger MIMONet variants. A complementary Code Ocean capsule with the containerized execution environment is additionally provided for peer review; a permanent DOI will be assigned upon publication. The raw ANSYS Fluent simulation outputs from which the preprocessed datasets were generated are archived by the authors and are available through the data-access pathway specified in the revised Code Availability and Data Availability statements.

Revision: Code Availability section consolidated to provide the public GitHub URL (hosting code, datasets, and trained models), the Code Ocean peer-review link, and the dataset access pathway for raw CFD outputs.

RESPONSE TO REVIEWER #2 (R2)

We thank Reviewer #2 for a technically rigorous review. The reviewer clearly has deep expertise in PDE observer theory and control-theoretic state estimation. We address each concern with full technical precision.

We begin with a clarification that is essential to contextualizing every response that follows.

1. *R2 comment: The paper presents an approach to using machine learning to develop state estimators for systems described by partial differential equations (PDEs). This is an active and lively subject within control systems and might or might not lend itself to machine learning. While mentioning system observers such as the Kalman filter and Luenberger estimators, they do not appear to appreciate what these systems do. They are dynamic systems which take the multiple measurements and yield estimates of the full system state, perhaps at different times. The system state is not pointwise the same as the measurements. [Full detailed comments follow in each section below.]*

Authors' Response: The review revealed that the original manuscript did not distinguish sufficiently clearly between classical PDE state estimation and the nonrecursive boundary-to-field reconstruction problem considered here. The distinction is not semantic. It concerns the estimation formulation, required assumptions, temporal role, and deployment regime.

Control-theoretic observers - including the Kalman-filter family, Luenberger design, moving-horizon estimation, and the boundary observers cited by Reviewer 2 - can estimate distributed states from boundary measurements when a governing model and observation operator are available. The reviewer's cited papers establish exactly this capability, and we now cite them as a complementary reference class rather than as a contrast case. MIMONet addresses a different deployment formulation. It learns offline a nonrecursive map from prescribed boundary and operating inputs to a steady or terminal multichannel field, and evaluates that map through a single forward pass without integrating any governing PDE or solving any optimization problem online. PDE observers use an explicit dynamical model and sequential measurements to construct causal state estimates analyzed through estimation-error dynamics, stability, and convergence guarantees. MIMONet does not provide observer-error convergence guarantees. The two formulations are therefore complementary: observers are appropriate when an accurate online model is available and the goal is temporal state tracking; MIMONet targets settings in which an explicit governing model is not available or solved/integrated during online inference and the goal is amortized steady or terminal field reconstruction within a tested operating envelope.

The cited references establish relevant context: they address 1-D hyperbolic PDE boundary observers with provable convergence under known dynamics. Their assumptions differ from ours in four ways: (1) they require a specified governing PDE at every inference step; (2) they perform recursive causal estimation in time; (3) their convergence guarantees derive from the known model; (4) their targets are 1-D spatial domains under traffic-flow or ODE-PDE cascade dynamics rather than steady 2-D multichannel thermofluid fields on unstructured meshes. We cite them as complementary methods and discuss them in that capacity in the revised Section 2.1.

We respect Reviewer #2's expertise in PDE observer theory. The revised Section 2.1 ("Problem Class Distinction: Operator Learning vs. State Estimation") formalizes this distinction and presents the two formulations as complementary.

2. *R2 comment: Line 73 states: Existing virtual-sensing models are intrinsically localized: they estimate only where sensors are present, failing precisely where measurements are absent or infeasible. This indicates a fundamental misunderstanding and misrepresentation of state estimation. The state is normally a finite-dimensional vector and the system models have time as a free variable, hence the central role of time in delivering predictions or smoothed results.*

Authors' Response: The reviewer is correct that state observers can estimate unmeasured states and, in distributed-parameter formulations, spatially distributed fields beyond the sensor locations. We do not dispute this. However, Line 73 refers to the virtual sensing literature specifically, GNNs, LSTMs, SVMs applied to sensor fusion, <u>not to PDE observer theory</u>. Many data-driven soft-sensing implementations are trained for prescribed output quantities or coordinates and do not natively provide a coordinate-conditioned field representation. Although such models could be redesigned for dense field output, the methods considered in the cited virtual-sensing literature were not formulated or validated for arbitrary-coordinate, multichannel reconstruction on irregular unstructured meshes.

We have also added a footnote clarifying that classical PDE observers perform recursive causal estimation in time under a known dynamical model and produce estimates analyzed through estimation-error dynamics, whereas MIMONet learns a nonrecursive map to steady or terminal fields with no online model requirement. The footnote explicitly acknowledges that observers can estimate spatially distributed states when a governing model is available, and it identifies the deployment-regime difference rather than an output-dimensionality difference.

Revision: Introduction and New Section 2.1

3. *R2 comment: Line 84 says: Consequently, no existing virtual-sensing method satisfies the central requirement of real-world diagnostics: reconstructing complete, physically coherent fields from sparse, heterogeneous measurements. The approach proposed in the paper seeks to simulate the PDE system and to use machine learning to fit finitely parametrized models, which are presumed to be universally applicable for changing operating conditions.*

Authors' Response: The reviewer characterizes our approach as fitting "finitely parametrized models presumed to be universally applicable." We agree that all neural networks are finitely parametrized; this is not in dispute.

We agree that the original wording could be interpreted as claiming universal applicability outside the training distribution. That claim was neither justified nor intended and has been removed. MIMONet is intended to approximate the boundary-to-field map over the operating envelope represented by the training distribution, with limited local extrapolation characterized separately. The revised manuscript supports its generalization claims empirically rather than through the universal approximation theorem, which is an existence result and does not establish finite-data generalization, sample efficiency, or accuracy for the trained network. The new data-scaling study

characterizes how test error varies with training-set size over five evaluated fractions, and the new out-of-distribution study evaluates a controlled 15% parameter shift beyond the training bounds. These results establish performance only for the evaluated geometries, CFD solver, physical models, and parameter ranges. The function-space distinction between finite-dimensional regression and operator approximation is retained and formalized in revised Section 2.1.

The relevant distinction is between coordinate-fixed regression, in which the predicted quantities and locations are prescribed during model construction, and field-valued operator approximation, in which the learned map is parameterized by spatial coordinates and represents a multichannel field over the domain. Although both inputs and outputs are discretized during computation, the operator formulation retains an explicit coordinate dependence that supports evaluation on alternative query sets. We have added a paragraph in the new Section 2.1 making this theoretical foundation explicit and citing the relevant approximation theorems.

We have also revised Line 84 to be more precisely scoped to the virtual sensing and soft sensing literature, and to acknowledge that classical observers provide complementary capabilities for time-domain state estimation.

Revision: Line 84 was narrowed to the relevant virtual-sensing literature, and Section 2.1 now defines the coordinate-conditioned operator formulation and its empirical scope. Universal-approximation results are cited only as representational background and are not used to support finite-data generalization or stability claims.

4. *R2 comment: Line 99: Moreover, neural operators trained across a range of possible operational conditions relax the requirement for continuous retraining when new operating regimes are encountered. There is no analysis of how the number of experiments might relate to the accuracy of the fits and the features of the underlying problem.*

Authors' Response: We have added a data-scaling study in new Section 2.8, "Sample Complexity and Convergence Analysis," with a three-panel figure covering all three simulated case studies. MIMONet was trained on five nested training fractions, 10%, 25%, 50%, 75%, and 100%, for each case. The PWR subchannel sweep uses N_{train} = 320, 800, 1,600, 2,400, and 3,200 and is evaluated on the fixed 1,000-sample test set. The heat-exchanger sweep uses N_{train} = 108, 270, 541, 812, and 1,082 and is evaluated on the fixed 310-sample test set. The LDC sweep uses N_{train} = 320, 800, 1,600, 2,400, and 3,200 and is evaluated on the fixed 988-sample test set.

Results: mean relative L^2 error decreases over the five training-set fractions for all three experiments. We fit a least-squares power law to each curve; the fitted exponents are $\gamma = 0.66$ (subchannel), $\gamma = 0.43$ (heat exchanger), and $\gamma = 0.58$ (LDC). These exponents are descriptive summaries of the observed finite-range behavior and are reported as such. They are not presented as theoretical sample-complexity rates or asymptotic convergence guarantees, and we do not infer a formal convergence knee from five data points. The displayed fit uncertainty reflects regression uncertainty over the five measured dataset sizes, not variability across independent model-training runs. Accordingly, the fitted exponents are used only as descriptive summaries of the observed finite-range trends.

Across the evaluated training fractions, the observed test errors decrease as the number of training samples increases. Because each fraction was evaluated using a single training realization, these curves characterize empirical data dependence for the reported training protocol but do not quantify seed-to-seed variability or establish formal sample-complexity rates. The fitted exponents are therefore used only as descriptive summaries of the finite-range trends. The universal approximation theorem for operators (Chen and Chen 1995; Lu et al. 2021) is an existence result; it does not provide finite-sample learning rates or optimization guarantees.

5. *R2 comment: There is no requirement of stability, be it the dynamics of the system or the numerics of the simulator, stated.*

Authors' Response: This concern addresses two distinct stability requirements: (a) the stability of the learned operator; (b) the numerical stability of the simulations used for training data. We address both.

For operator stability, we have added an empirical local sensitivity analysis in new Section 2.9. For 1,000 random perturbation directions sampled at each of 20 magnitudes, with $\|\delta\|_2 \in [0.01, 0.50]$, on the 310-sample heat-exchanger test set, the empirical 99th-percentile sensitivity values are $L_p \approx 0.88$ for pressure and L_u_z ≈ 0.37, L_u_y ≈ 0.64, and L_u_x ≈ 1.02 for the three velocity components. These statistics characterize observed local amplification over the sampled test inputs and perturbation directions in normalized input coordinates. They are not global Lipschitz constants, do not establish stability of the physical dynamics, and do not constitute a closed-loop observer guarantee. The same noise injection applied to the three operator baselines confirms that MIMONet shows substantially smaller empirical sensitivity than GeoFNO, NOMAD, and KCN under identical perturbation conditions, as reported in Section 2.10.

Because the subchannel and heat-exchanger datasets consist of converged steady-state solutions, we do not claim a dynamical stability analysis of the underlying physical systems. For numerical convergence and dataset quality: we have explicitly stated in the Methods section that all ANSYS Fluent simulations were converged to scaled residual criteria of 10^{-6} for the continuity, momentum, energy, turbulent kinetic energy, and turbulent dissipation rate equations. Residual convergence histories were archived for all 11,483 simulation runs; no diverged or unconverged solutions were included in the dataset; Material properties were temperature-dependent and updated internally by the CFD solver during each simulation.

Revision: New Section 2.9 added; Methods section updated with explicit CFD convergence criteria.

6. *R2 comment: Line 120 goes on: The shift from "solve once per boundary condition" to "learn the operator itself" is therefore the decisive scientific break that makes real-time inference in inaccessible regions physically plausible. This is never demonstrated other than by simple*

smooth examples chosen by the authors. Such uncritical statements abound in the paper. Further, apart from liberal use of words like "real-time," the method uses functional expansions of variables as functions of time on a fixed finite interval.

Authors' Response: We agree that the original wording was too categorical and we have now removed the phrase "decisive scientific break." The examples are simulation-based and do not establish universal applicability. However, they extend beyond elementary smooth test cases. The PWR subchannel case is governed by RANS equations closed with a k–ε turbulence model in a confined multi-rod geometry, with wall-adjacent thermal gradients, cross-stream secondary flows, and turbulent kinetic energy production on a 1,733-node unstructured mesh with no interior measurement access. The heat-exchanger case contains pressure, three velocity components, recirculation cells, stagnation regions, and geometric junctions on a 3,977-node unstructured mesh. High-fidelity CFD requires hundreds of seconds per instance, whereas MIMONet evaluates the corresponding full field at millisecond-scale latency on the tested data-center accelerators within the trained operating envelope. The revised manuscript makes a narrower empirical claim: within these three simulated configurations, offline operator learning reduces repeated field-evaluation time from the CFD scale to the measured inference latency while maintaining the reported reconstruction accuracy.

We have nonetheless revised Line 120 to remove the word "decisive" and ground the statement in the empirical evidence: *"The shift from instance-specific solving to an offline-learned operator enables millisecond-scale field evaluation after the required input descriptor is available, as demonstrated across the three simulated configurations."*

Revision: The "decisive scientific break" wording revised to ground the claim empirically in the CFD solver time vs MIMONet inference latency comparison stated in the Introduction (hundreds of seconds for CFD versus millisecond-scale MIMONet inference) and quantified in Section 2.12 (Table 3).

7. *R2 comment: Time does not enter the picture, since the whole solution is provided as an operator on the whole input signal, not a prediction using data up to a particular time.*

Authors' Response: This is accurate and by design. The steady-state subchannel and heat exchanger problems target steady-state spatial field reconstruction - temporal recursion is neither required nor appropriate for these physical scenarios. For the lid-driven cavity, the complete lid-velocity history $V(t)$ is supplied as an input function and the target is the spatial field at the prescribed final simulation time $t = T$. This is consistent with standard operator-learning formulations in which an input function, such as a forcing history or initial condition, is mapped to a terminal or spatial solution field, as exemplified by DeepONet and Fourier Neural Operator studies. The reviewer is correct that this work is not a causal filter or predictor. In this study, 'real-time' refers strictly to the measured wall-clock latency of a forward evaluation after the required input descriptor has become available. It does not denote causal filtering, online temporal prediction, or closed-loop observer performance. Any comparison with physical response times is restricted to the specific systems and operating regimes evaluated in the manuscript. The subchannel and heat-exchanger cases are steady-state mappings; in the lid-driven-cavity case, the full lid-velocity history is an input function and the target is the final-time spatial field. We have

added this definition to the manuscript and revised the abstract and Discussion to prevent the latency claim from being read as a temporal observer. We have added a clarifying paragraph in the new Section 2.1 distinguishing between temporal prediction tasks (where time-recursive observers are appropriate) and steady-state field reconstruction tasks (where operator learning is appropriate).

Revision: New Section 2.1 paragraph added; clarification note inserted near the lid-driven cavity formulation. (Revision confirmed in the revised manuscript at the start of Section 2.1 and in the lid-driven cavity formulation paragraph)

8. *R2 comment: There is a lot of similar word salad such as, Line 234: Because $\mathcal{G}\theta(\boldsymbol{u})$ is defined for any query set, evaluation on inaccessible coordinates is obtained by replacing Pi_Y with arbitrary point sets. Line 243: This multiplicative fusion preserves permutation invariance across modalities and yields a compact, modality-agnostic summary of the inputs. Physically, the product structure serves as a low-rank surrogate for multiplicative couplings in the governing PDEs, e.g., convective transport, encouraging a shared latent psi that supports joint decoding of all output channels. The absence of any defined glossary just makes assertive.*

Authors' Response: We accept this criticism. The original manuscript used physical interpretations of mathematical objects without adequate prior definition. We have made two additions:

First, a Notation and Glossary Table (new Table S1 in Supplementary Material) defining all mathematical symbols, their dimensions, and their physical interpretations.

Second, Lines 234 and 243 have been revised to provide mathematical justification where physical interpretation was previously asserted:

For Line 234, the statement now reads: Because the spatial coordinate $\boldsymbol{r}$ is an explicit input to the trunk decoder, the learned representation defines a coordinate-conditioned prediction $\mathcal{G}\theta(\boldsymbol{u})(\mathbf{r})$ and can therefore be evaluated at arbitrary query coordinates within the represented domain. This architectural property enables evaluation on coordinate sets different from those used during training, although accuracy at previously unsampled coordinates remains an empirical generalization question rather than a consequence of the L^2 formulation alone.

For Line 243, the multiplicative fusion is now described as follows. For output channel o, the prediction has the form

$\hat{s}_o(\mathbf{r}, \{u_i\}_{i=1}^n) = \sum_{j=1}^I \tau_{j,o}(\mathbf{r}) \prod_{i=1}^n \varphi_{i,j}(u_i) + \beta_o$, where $\prod_{i=1}^n \varphi_{i,j}(u_i) = \psi_j$.

Each summand is separable across the modality-specific encoded features and the coordinate-dependent decoder. The product fusion introduces explicit cross-modal interactions before coordinate decoding. It does not by itself enforce the governing equations, incompressibility, momentum balance, or energy conservation. Because the encoders are modality-specific, permuting the input modalities changes the encoded features; the elementwise product is commutative only after modality-specific encoding, while the complete mapping remains

modality-aware. The permutation-invariance claim and Supplementary Note 1 have therefore been removed.

Revision: Notation/Glossary Table added (Table S1); Lines 234 and 243 revised with the CP-type factorization description above; permutation-invariance claim and Supplementary Note 1 removed.

9. *R2 comment:* ***Summary:*** *I cannot recommend this paper for publication because its contribution is obscure at best and undemonstrated at worst. It makes inadequate and imprecise contact with state estimation methods for PDEs. The conclusions are asserted but not supported by mathematical argument.*

Authors' Response: We appreciate that the original manuscript did not make sufficiently precise contact with PDE observer theory and contained several statements that exceeded what the architecture and experiments established. The revised manuscript corrects this directly. The revised formulation identifies MIMONet as a nonrecursive, learned boundary-to-field map for steady or terminal reconstruction and distinguishes it from model-based dynamic observers through their assumptions, inputs, temporal roles, outputs, and guarantees. Empirically, MIMONet achieves mean relative L^2 errors from 0.27% to 5.0% across the three experiments and outperforms the three evaluated operator baselines (GeoFNO, NOMAD, and KCN) on the reported test sets. The empirical local sensitivity statistic stays below 1.02 across all output channels over the sampled perturbation set (Section 2.9), the data-scaling study shows monotone error reduction over five training-set fractions (Section 2.8), and an exploratory test on 50 cases placed 15% outside the training ranges produced a mean error increase of approximately 1.8× relative to the in-distribution test condition (Section 2.11). The revised Section 2.1 presents MIMONet and PDE observers as complementary formulations with distinct assumptions and guarantees, ensuring that readers from the state-estimation community can accurately position this contribution relative to classical observer theory.

10. *R2 comment: I provide below some recent references on PDE observers, which get the estimation problem right without looking for inserting machine learning too early. Note that these are state estimators with a sense of time, e.g., predictors or smoothers. To yield function estimators, you need to propose a basis and estimate coordinates. References: H. Yu et al. (2021), J. Wu et al. (2024), and F. Ferrante et al. (2020)*

Authors' Response: We thank the reviewer for providing these specific references. All three works are now cited in the revised manuscript, and we use them to clarify the distinction between model-based PDE observers and the operator-based virtual-sensing formulation studied here. These papers demonstrate that PDE observers can estimate spatially distributed states from boundary measurements when the governing dynamics, boundary conditions, and observation model are specified. We fully acknowledge this capability and present these methods as a complementary reference class rather than as methods that cannot estimate hidden states.

The distinction is the deployment formulation. The cited observer works are time-domain estimators: they propagate a state estimate through a specified dynamical model and analyse convergence through observer-error dynamics. MIMONet instead learns an offline, nonrecursive

boundary-to-field map for steady or terminal multichannel field reconstruction and evaluates that map with a single forward pass without integrating the governing PDE online. To obtain function estimates, observer formulations require a prescribed spatial model, basis, grid, or discretization whose coordinates are estimated through the observer. MIMONet uses a coordinate-conditioned trunk decoder so that the learned field can be queried at arbitrary coordinates within the represented reconstruction domain.

We have revised Section 2.1 to cite Yu et al. (2021), Wu et al. (2024), and Ferrante et al. (2020), and to state explicitly that these observer methods are appropriate when an online governing model is available and the target is temporal state tracking. The present contribution targets the complementary regime of amortized spatial field reconstruction from sparse boundary or point measurements when real-time PDE integration is not part of the online inference loop.

RESPONSE TO REVIEWER #3 (R3)

We thank Reviewer #3 for identifying important weaknesses in the comparative framing, efficiency analysis, validation scope, and presentation of the original manuscript. We agree that these issues were not addressed adequately in the submitted version. The revised manuscript responds through new controlled comparisons, additional robustness and out-of-distribution experiments, a clearer limitation statement concerning simulation-only validation, and substantial restructuring of the presentation.

Reviewer #3 (Remarks to the Author): The manuscript proposes a neural network model for real-time virtual sensing, with special focus on thermal and fluid dynamics. I find the paper contributions to be narrow for the Nature Communications readership, and therefore I do not recommend the paper for publication.

Authors' Response: We thank the reviewer for raising this concern and address it directly. The original manuscript's nuclear-focused framing did not sufficiently communicate the cross-domain scope of the contribution, and the revision addresses this directly. The revised Abstract now opens with a framework-level statement before introducing nuclear thermal-fluid systems as the primary motivating application. The Introduction's first paragraph frames inaccessible-field inference as a general challenge shared across energy, environmental, and industrial systems. The Discussion closing paragraph names all four validated domains and their span of six orders of magnitude in spatial scale.

The revised manuscript validates the framework beyond the high-fidelity flow configurations through three independent real-world experiments, each governed by distinct physics. The PEMFC experiment tests internal current-density and temperature reconstruction in an electrochemical energy device governed by electrochemical kinetics and Joule heating. The IEA-Wind experiment tests hub-height wind-speed recovery from lower-level in-situ tower measurements under atmospheric boundary-layer conditions. The GLORYS12 experiment tests multimodal ocean-state reconstruction from disjoint sparse temperature and salinity networks, including recovery of sea-surface height from zero direct SSH sensors, under thermohaline oceanographic dynamics. Together with the three engineering-grade flow configurations, these four validated domains cover spatial scales from a laboratory-scale fuel cell to the North Atlantic basin, and four distinct physical regimes that are not reducible to a single governing equation, scale, or sensing modality. The unifying structure is not a shared governing equation or a single application domain, but a shared inference problem: sparse accessible measurements must substitute for physically inaccessible or unmeasurable field observations. This problem recurs across many high-consequence scientific and engineering domains, including nuclear engineering, electrochemical energy systems, wind-energy monitoring, geophysical observation, battery thermal management, cardiovascular hemodynamics, structural health monitoring, and aerospace thermal protection. The revised Discussion explicitly enumerates these connections. The cross-disciplinary character of the inference problem and the demonstrated breadth of the solution are precisely what motivate Nature Communications as the appropriate venue. Across the three real-world experiments, MIMONet advances over practical classical baselines by 72% to 84% on real-world field data. The sea-surface-height result is particularly important for establishing the cross-disciplinary scope: MIMONet recovers a physically coupled geophysical

field at 9.11% error from zero direct SSH sensors, compared with 37.7% for climatology, a 4.1-fold lower error corresponding to a 76% error reduction. This is a cross-modal inference setting in which classical spatial interpolation cannot operate directly under the stated sparse-sensor protocol because the target channel carries no direct observations in the input. The locality-based learned baseline KCN collapses to 37.84% on SSH, essentially identical to climatology, confirming that the advance is architectural rather than a generic property of machine learning. Fourier-based spectral operators require either a uniform computational representation or, in the GeoFNO setting, a learned diffeomorphic correspondence from the physical domain to a latent uniform computational domain; they are therefore not native sparse-sensor comparators under the stated protocol without an additional representation step. The revised manuscript defines operator-based virtual sensing as a general methodology for inaccessible-field observability, instantiates it in a demanding safety-critical energy-system setting, and validates the same sparse-to-field reconstruction principle across independent physical sensing regimes spanning electrochemistry, atmospheric science, and physical oceanography. The 72% to 84% advances over practical classical baselines on real-world physical sensing data, including cross-modal recovery of a geophysical field with zero direct sensor observations across six orders of magnitude in spatial scale, constitute a contribution whose breadth and demonstrated impact are commensurate with the Nature Communications readership.

1. *R3 comment: It is not clear what fundamental challenge is solved by the proposed method that cannot already be handled by existing approaches. There is a wide variety of virtual sensing methods in the literature, from classical ones like Kalman filters, MHE, and basic machine learning tools like SVMs, to physics-informed neural networks or identification models like SINDy. Although some of these methods are acknowledged in the Introduction, the authors do not benchmark the performance of their method against them, neither in computational time nor in performance analysis. For instance, the noise robustness assessment is made exclusively within the context of the proposed method (and its variations). For the considered case studies, it is not clear why other physics-based ML models could not be readily deployed to solve these problems.*

Authors' Response:

We thank the reviewer for raising five interrelated concerns that we have addressed through coordinated changes across the revised manuscript: (i) the fundamental challenge solved by this work that is not addressed by existing approaches; (ii) the absence of a structured argument for why classical and physics-based ML alternatives cannot be deployed for this task; (iii) the lack of computational-time benchmarking against alternative architectures; (iv) the lack of performance benchmarking; and (v) the noise-robustness assessment being a self-comparison. The revised manuscript addresses each of these explicitly. *Before responding to the individual sub-concerns, we wish to clarify an overarching point that motivates our approach to the comparisons requested.*

Scope and interpretation of the requested comparisons. We agree that the original manuscript did not explain sufficiently why the cited method classes were not included in the quantitative tables. Direct benchmarking is not impossible in principle, but it is not straightforward because the methods impose different assumptions, consume different forms of information, and return different types of estimates. Classical observers require an explicit dynamical and observation

model and sequential measurements; standard PINNs solve individual PDE instances through optimization; SINDy identifies governing dynamics from time-resolved state data; and conventional soft-sensing regressors are commonly trained for prescribed output quantities or locations. The present task instead evaluates an offline-learned, coordinate-conditioned map from boundary and operating inputs to steady or terminal multichannel fields. We therefore added two complementary forms of comparison: a structured capability analysis for methods whose assumptions differ from the present formulation, and controlled quantitative comparisons for operator architectures that can be trained on the same boundary-to-field datasets and evaluated under identical output requirements. We do not claim that the other method classes are incapable of being adapted. Rather, such adaptations would change their input assumptions, online computational requirements, or target formulation and would require a separate study. We elaborate on each sub-concern below.

(i) The fundamental challenge solved by this work. The revised manuscript introduces a new subsection - **Problem Class Distinction: Operator Learning vs. State Estimation** (Section 2.1) - that formalizes how the present boundary-to-field reconstruction setting differs from the cited methods in its model assumptions, temporal role, online computation, coordinate dependence, and target output. Classical state observers perform recursive estimation under an explicit dynamical and observation model and can estimate unmeasured finite-dimensional or distributed states, depending on the underlying formulation and spatial representation. The present work addresses a different deployment setting: an offline-learned, nonrecursive boundary-to-field map that returns steady or terminal multichannel fields through coordinate-conditioned evaluation without integrating the governing equations during online inference. The target of this work is fundamentally different: to learn a nonlinear operator $\mathcal{G}: \mathcal{U} \rightarrow L^2(\mathcal{Y}; \mathbb{R}^m)$ that maps an admissible boundary functional $\boldsymbol{u}$ to a continuous interior field $\mathbf{s}(\mathbf{r})$, evaluable at any coordinate $\mathbf{r} \in \mathcal{Y}$, including locations that are physically inaccessible to sensors and spatially disjoint from the input measurement region, $X \cap \mathcal{Y} = \emptyset$. Classical observer formulations require an explicit dynamical model and perform recursive temporal estimation, whereas the present approach learns an offline, nonrecursive boundary-to-field map from simulation pairs. This deployment-regime difference, not an output-dimensionality difference, motivates the operator-learning formulation. Section 2.1 develops this distinction with formal definitions, and Section 2.2 - **Method-Class Landscape and Architectural Requirements for Field-Level Virtual Sensing** - formalises the implied capability requirements in a six-dimension matrix (Table 1).

(ii) How the cited classical and physics-based ML methods differ from the present inference setting. Section 2.2 of the revised manuscript provides a method-class-by-method-class logical argument. Model-based observers, including Kalman-filter and moving-horizon formulations, require an explicit state-evolution (transition model) and observation model and perform recursive estimation from sequential measurements. Depending on the chosen state representation, they may estimate distributed states, but arbitrary-coordinate reconstruction on an irregular mesh is not obtained automatically and depends on the spatial model and observer design. Conventional SVM, RNN, GNN, and kernel-based soft sensors are commonly configured for prescribed output variables or locations; they can be redesigned for dense output, but the cited implementations were not developed or validated for coordinate-conditioned multichannel reconstruction on disjoint sensing and target regions. Standard PINNs typically solve each boundary-condition instance through test-time optimization (training), although parameterized and operator-learning extensions

can amortize part of this cost. SINDy and related equation-discovery methods generally require time-resolved state observations, derivative estimates, and a specified candidate library, and their primary output is an identified dynamical model rather than a directly evaluable boundary-to-field map. These differences explain why the methods are discussed through their assumptions and capabilities rather than treated as interchangeable implementations of one benchmark. The revised Section 2.2 develops these arguments in dedicated paragraphs and Table 1 records the resulting capability shortfalls in a unified matrix. We emphasize that this is not a critique of the named methods as methodologies, each is powerful in its intended regime, but an analysis of why their standard formulations are not directly commensurate with the boundary-to-field evaluation protocol used in this work.

Following Response Figure 1 summarizes their capabilities:

- Coordinate-input FNN formulations, including standard instance-specific PINNs, occupy the upper-left quadrant because they accept spatial coordinates but do not ordinarily amortize inference across an input-function space. Parameterized PINNs and related extensions may alter this classification. Representative SVM and regression-based virtual sensors occupy the lower-right quadrant because their outputs are commonly defined at locations fixed during model construction. Coordinate-conditioned variants could be designed, but the cited implementations do not provide that capability.
- Standard Kalman/MHE formulations and SINDy are shown outside the lattice because the schematic organizes amortized coordinate-conditioned predictors, whereas these methods primarily address recursive state assimilation and equation discovery, respectively.

Among the method classes examined in the revised manuscript, coordinate-conditioned neural operators provide direct support for both amortization across input functions and evaluation on alternative query sets. The schematic is intended to organize the assumptions of representative formulations, not to establish an impossibility theorem for all potential adaptations of other method classes.

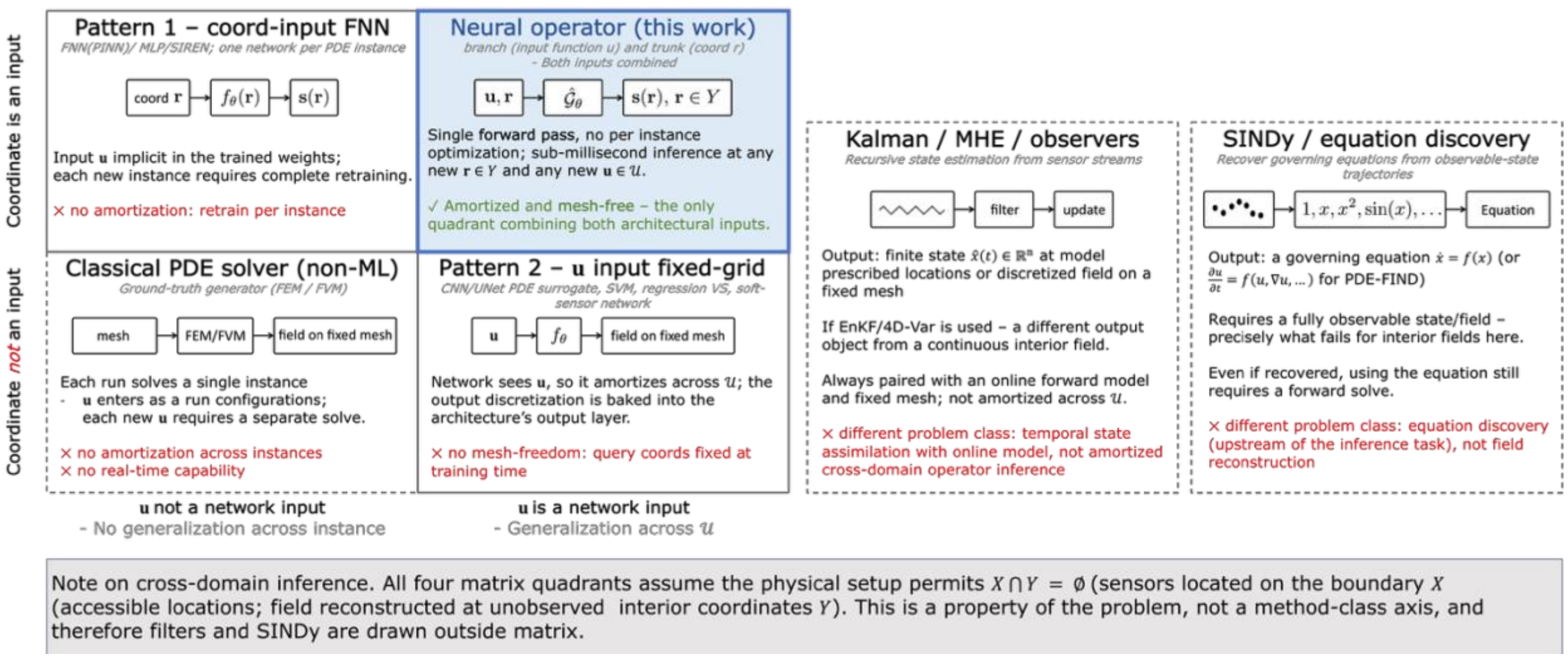

Response Figure 1. Inference-regime landscape for field reconstruction. The 2 × 2 matrix in the LHS is organized by the two architectural inputs a learned network may receive: the input function $\boldsymbol{u}$*, horizontal axis: amortization across the input space* $\mathcal{U}$*, and the query coordinate* $\boldsymbol{r}$*, vertical axis: mesh-freedom. The four resulting architectural patterns are: classical PDE solver, non-ML reference, lower-left; coordinate-input FNN/MLP/SIREN, including PINN as a training-time variant, Pattern 1, upper-left;* $\boldsymbol{u}$*-input fixed-grid networks, including CNN/U-Net surrogates, SCMs, and regression-based virtual sensors, Pattern 2, lower-right; and neural operator, this work, upper-right, highlighted. Kalman/MHE-class state estimators and SINDy-class equation-discovery methods are drawn outside the matrix because they target structurally distinct inference regimes: recursive state assimilation with an online model and upstream equation discovery, respectively. Direct accuracy comparisons across all method classes would require method-specific reformulations and additional assumptions to make their inputs and outputs commensurate. The main-text quantitative comparisons therefore focus on architectures that can be trained and evaluated under the same boundary-to-field protocol, while Table 1 documents the assumptions and intended outputs of the broader method landscape.*

(iii) Computational-time benchmarking. The revised manuscript now contains an architecture-level efficiency comparison on identical hardware (Section 2.12, Table 3). All four operator architectures (MIMONet, GeoFNO, NOMAD, KCN) are profiled on the same GPU under the identical workload (B=1, P = 3,977 query points), reporting parameter count, profiler-estimated FLOPs, synchronized wall-clock latency, and energy per inference. Energy was measured over sustained windows of repeated forward passes by integrating energy per inference computed per pass as mean board power × measured latency ($E_i = P_i t_i$), averaged over the N = 1,000 passes — total board draw, no idle-baseline subtraction.

The original cross-GPU figure (A40 to GH200) is retained as Supplementary Fig. S1 to characterize hardware portability separately from architectural efficiency, addressing the reviewer's distinct concern that the original energy claim conflated GPU-generation effects with model-architecture effects. We do not report a full test-set PINN benchmark because a standard PINN would require a separate optimization for each boundary-condition instance and therefore would not share the same amortized inference protocol. We now state this exclusion explicitly and

avoid presenting literature-reported PINN timings as if they were measured under our workload. Parameterized PINNs and physics-informed operators are recognized as relevant alternatives for future controlled comparison.

(iv) Performance benchmarking. Per-case quantitative comparisons against the neural-operator family, GeoFNO, NOMAD, and KCN, the three baselines that target a compatible inference regime, are reported in Table 2 panel (a), (b) and (c) of the revised manuscript for the lid-driven cavity, PWR subchannel and heat exchanger problems respectively. MIMONet outperforms each baseline by a substantial margin across all output channels and all three case studies on the evaluated datasets and training protocol. This comparison establishes performance relative to the three evaluated operator implementations; isolating the contribution of individual architectural components would require parameter-matched ablation experiments, which we identify as future work. For the method classes that target an incompatible regime, the capability matrix (Table 1) records the architectural shortfalls; running these classes on tasks they were not designed for would not constitute a fair comparison, and Section 2.2 explains the structural reasons for their non-inclusion in the per-case accuracy tables.

(v) Noise robustness as a cross-architecture benchmark. We agree that the original noise-robustness experiment compared only MIMONet's deterministic and MC-Dropout variants and was therefore a self-comparison. In this revised version, all four architectures (MIMONet, GeoFNO, NOMAD, KCN) were evaluated deterministically (single forward pass at each noise level) under identical test inputs and normalisation, so the comparison isolates the robustness of the trained deterministic mappings across architectures as shown in Fig. 9. MC-Dropout MIMONet $N = 20$ passes) is reported alongside the deterministic architectures as a separate uncertainty-aware variant; it is not used to claim architectural superiority over single-pass baselines.

In summary, the revised manuscript now: (i) defines the boundary-to-field problem and distinguishes it from recursive state estimation; (ii) compares method classes through their assumptions and intended outputs; (iii) evaluates four operator implementations for accuracy and efficiency under common conditions; and (iv) extends robustness evaluation across architectures and structured perturbations. These revisions clarify the contribution without claiming that other method classes are incapable of adaptation.

2. *R3 comment: I find the claims/analysis on energy efficiency to be even more lacking, given that the energy comparison is made across GPUs rather than across model architectures. Thus, the authors are actually evaluating the neural network inference efficiency of a GPU rather than the efficiency of their model relative to others.*

Authors' Response: The reviewer is precisely correct. The original GPU-progression comparison characterized hardware portability across NVIDIA data-center generations under a fixed MIMONet model; it does not constitute an architectural comparison. We accept this criticism without reservation.

The revised manuscript now contains a dedicated **architecture-level efficiency analysis in new Section 2.12 (Table 3, "Architecture-level inference efficiency comparison")**, reporting

parameter count, FLOPs per inference (via torch.profiler), wall-clock latency (measured after warm-up with explicit CUDA synchronization), energy per inference (computed per pass as the mean NVML board power times the measured latency, $E_i = P_i t_i$, averaged over 1,000 passes; total board draw, no idle-baseline subtraction), and mean relative L^2 error for all four operator architectures (MIMONet, GeoFNO, NOMAD, KCN) on a single NVIDIA H200 GPU under identical workload conditions (B = 1, P = 3,977 query points, N = 1,000 timed passes, float32 precision).

Table 3 of the revised manuscript (Section 2.12)

Model	Params	FLOPs	Latency (ms)	Power (W)	Energy (J/iter)	Mean L2 (%)
MIMONet	1.76 M	3.14 G	0.35	129.2	0.046	**0.95**
GeoFNO	2.70 M	1.58 G	3.08	149.6	0.460	5.50
NOMAD	113 K	0.53 G	**0.23**	157.2	**0.036**	16.4
KCN	12.9 K	0.06 G	1.75	123.6	0.216	16.3

The revised comparison treats accuracy, latency, and energy as separate axes rather than collapsing them into a single dominance claim. Under the common H200 protocol, MIMONet achieves the lowest mean relative L^2 error among the four evaluated architectures. GeoFNO is the closest accuracy competitor in this benchmark but exhibits higher measured latency and energy per inference under the identical H200 workload. NOMAD shows lower measured latency and energy than MIMONet under this workload, reflecting its smaller parameter footprint, but its mean reconstruction error is substantially higher. KCN is the most compact model at 12.9K parameters but has the highest mean reconstruction error among the four evaluated implementations. These measurements reflect the complete implementations used in this study, including all encoding, decoding, coordinate handling, and memory operations, and we report them without attributing the differences to any single architectural component. The branch-trunk contraction has complexity $O(BPIm)$ with respect to the contraction dimensions, whereas the measured end-to-end cost also includes branch encoding, trunk evaluation, memory operations, and implementation overhead. The revised manuscript therefore emphasizes measured end-to-end latency, FLOPs, and energy rather than claiming an unconditional asymptotic advantage.

The original cross-GPU progression figure (A40 → A100 → H200 → GH200) has been moved to **Supplementary Figure S1** under the explicit heading "Hardware portability characterisation," and clarifying text states that this panel isolates the hardware-generation contribution under a fixed model rather than making any architectural-efficiency claim. The full architecture × GPU × case efficiency matrix (4 architectures × 4 GPUs × 3 case studies) is reported in **Supplementary Table S8**; the observed cross-architecture ranking in main-text Table 3 is preserved across the tested GPUs, indicating that the measured ordering is not unique to the H200 under the evaluated workloads.

Revision: New main-text **Section 2.12** with **Table 3** (architecture-level efficiency on identical H200 hardware) reporting FLOPs, latency, power, and energy per inference; cross-GPU

progression figure moved to **Supplementary Figure S1**; full architecture × GPU × case matrix added as **Supplementary Table S8**.

3. *R3 comment: Given the overemphasized motivation of the paper on the need for real-time virtual sensing methods for critical applications, I find the lack of model validation on real-world data to be a critical limitation of the work. The authors go through the effort of measuring the inference time and energy consumption of their model computations, but none of this matters if the model cannot be validated beyond simulated (highly smooth) data.*

Authors' Response: We thank the reviewer for raising this critical point precisely and for identifying the logical gap between the inference-time measurements and the absence of real-world validation. The reviewer is correct that the inference latency and energy measurements were not sufficient by themselves, and that the original manuscript had not been validated beyond solver-generated data. Both issues are addressed directly in the revised manuscript.

First, we have narrowed the nuclear CFD claims. We now state explicitly that the three nuclear CFD studies establish simulation-based feasibility within the evaluated operating envelopes for reactor-relevant inaccessible geometries. They do not by themselves constitute experimental validation, and performance on RANS-generated fields does not establish robustness to experimental artifacts, sensor-to-simulation domain shift, model-form error, or geometry mismatch. A revised Limitations paragraph states that experimental ground-truth interior thermal-hydraulic data from operating nuclear systems were not available for this study, and that scaled transparent-facility measurements, particle image velocimetry in non-nuclear geometries, and cross-solver validation against independently generated datasets remain necessary before operational nuclear deployment.

Second, a new Section 3 reports results on three independent real-world validation experiments.

The PEMFC experiment tests the same physical scenario as the nuclear motivation: an internal field that is measurable in the laboratory but cannot be obtained from any deployable sensor. MIMONet achieves 1.90 ± 0.51% channel-mean relative L^2 error from 10% sensor coverage, against 11.67% for the best classical baseline (IDW), an advance of +84%. The primary unmeasurable field, current density, reconstructs to 3.77 ± 1.03% versus 23.10% for IDW.

The IEA-Wind experiment provides validation on purely in-situ physical anemometry with no model or reanalysis component. MIMONet achieves 4.71 ± 0.15% at hub height (80 m) from lower-level sensors, against 27.86% for the best classical baseline, an advance of +83%. This experiment tests a real deployment regime in which the target location is not observed by the input sensor network and fixed-form laws fail under stratification. At 100 m, the power law reaches 95.92% error while MIMONet remains at 14.70 ± 0.59%.

The GLORYS12 ocean experiment tests the hardest architectural case: cross-modal recovery of a field with zero direct input sensors. MIMONet achieves 4.22 ± 0.06% channel-mean error, a +72% advance over the best classical baseline. SSH, which carries no sensors in the input, is recovered at 9.11 ± 0.17% against 37.7% for climatology, a 4.1-fold lower error corresponding to a 76% error reduction.

Third, we clarified the input-interface distinction for several existing physics-based and operator-learning methods. Some Fourier-based operator models can be applied to scattered

real-world sensor data only after the observations are represented on a uniform computational domain, or, in the GeoFNO setting, after learning or specifying a suitable diffeomorphic correspondence from the physical domain to a latent uniform computational domain. This does not make those methods incapable; rather, it means they are not direct end-to-end comparators under the native sparse-sensor input setting, because the required gridding step is itself a classical interpolation procedure. We evaluated these methods where architecturally applicable and documented why grid-based methods are not direct baselines on scattered-sensor tasks.

FNO is a Fourier-based spectral operator whose FFT computation is naturally defined on a uniform computational grid. GeoFNO extends this idea to irregular geometries by learning a deformation from the physical domain, which may be represented by point clouds, meshes, or design parameters, into a latent uniform computational domain on which the Fourier operator is applied. Thus, GeoFNO is not limited to regular grids in physical space. Its relevant assumption is that a suitable diffeomorphic correspondence between the physical domain and the uniform computational domain exists or can be learned for the task. This is a different input-interface assumption from the native sparse-sensor setting considered here, where the model is given scattered sensor values and coordinates and must reconstruct fields directly without first constructing a grid-valued input field. Applying FNO or GeoFNO to scattered PEMFC or ocean sensor inputs under our protocol would therefore require an additional representation step, such as mapping sparse observations into a computational grid or latent uniform domain. That representation step is not a neutral operation; it introduces an interpolation or deformation front end whose existence and accuracy are part of the problem. FNO and GeoFNO are therefore not treated as independent end-to-end sparse-sensor baselines on the fuel-cell and ocean tasks.

On the wind task, where the input is a natural one-dimensional vertical sequence indexed by height, FNO is a direct baseline and achieves 7.30 ± 1.16%, worse than MIMONet at 4.71 ± 0.15%. Even in the most architecturally favorable case for spectral operators, MIMONet retains a clear advantage. We also note that the IDW-plus-FNO pipeline referenced for transparency in the ocean section achieves approximately 4.4% channel-mean error over temperature and salinity only: because IDW requires direct sensor observations in the target channel and SSH carries none, the pipeline cannot produce any SSH prediction. MIMONet's 4.22 ± 0.06% channel-mean is therefore a three-channel mean that includes SSH at 9.11 ± 0.17%, a channel the two-stage pipeline cannot address. The channel-mean figures are not directly comparable: MIMONet achieves a lower mean over a strictly harder task.

KCN, a locality-based learned kriging network, can directly ingest scattered sensor inputs and was evaluated on all three tasks under identical conditions. The results provide the most important control experiment in the paper. On ocean temperature and salinity, channels with direct sensor coverage, KCN achieves 5.77% and 0.86% respectively, improving meaningfully over classical interpolation and confirming that the data are learnable and that KCN has adequate capacity. On SSH, however, which carries zero sensors in the input, KCN collapses to 37.84%, essentially identical to climatology at 37.7%. This result is decisive: learning capacity alone is not sufficient for cross-modal inference on an unobserved target channel. The limitation is structural and architectural, not merely a matter of training, tuning, or dataset size. MIMONet's coordinate-conditioned, cross-modal operator design is the enabling property. The revised manuscript does not rely only on smooth simulated data, and it documents why methods compatible with gridded or locality-based reconstruction do not automatically become deployable real-world virtual sensors.

Fourth, we connected the latency measurements to real-world accuracy. Methods that are compatible with smooth gridded CFD data do not automatically become direct native sparse-sensor virtual sensors under real-world sensing protocols. Fourier-based spectral operators require either a uniform computational representation or, in the GeoFNO setting, a learned diffeomorphic correspondence from the physical domain to a latent uniform computational domain. Locality-based learned interpolation collapses when the target channel is absent from the input. MIMONet succeeds on real-world field data because its branch-trunk formulation treats sparse sensor values and coordinates as the native input representation. The operator that achieves 0.35 ms inference at 46 mJ per field reconstruction on an NVIDIA H200 is the same operator that outperforms classical virtual sensing by +84% on fuel-cell internal-field reconstruction, +83% on hub-height wind reconstruction across international tower sites, and +72% on North Atlantic ocean-state reconstruction, all on real-world physical sensing data, with MIMONet using sparse sensor values and coordinates directly as the operator input. The efficiency result is therefore tied to demonstrated real-world accuracy rather than being an isolated hardware benchmark.

In summary, the revised manuscript now separates two distinct claims. The nuclear CFD studies establish simulation-based feasibility for reactor-relevant inaccessible geometries. The PEMFC, wind-tower, and ocean experiments establish significant performance advances over practical virtual-sensing baselines on real-world physical sensing data. This directly resolves the reviewer's concern that the original manuscript relied on smooth simulated data while emphasizing real-time virtual sensing for critical applications. Table R3-1 summarizes the three real-world experiments and the corresponding framework advances.

Table R3-1. Real-world experiment summary: MIMONet versus the best classical virtual-sensing baseline on each task.

All results are based on real measured data. Framework gain is computed as:

Framework gain = (Classical error − MIMONet error) / Classical error × 100%

Results are reported in Section 3 and in the real-world validation tables of the revised manuscript, including the per-domain result tables and the cross-domain synthesis tables. Final table numbers will be confirmed after compilation.

**Domain**	**Target field**	**MIMONet relative L^2 error (%)**	**Best classical relative L^2 error (%)**	**Framework gain**	**Virtual-sensing regime**
PEMFC fuel cell (R3 and Editor)	{J, T}, 10% sensor coverage	1.90 ± 0.51	11.67, IDW	+84%	CDM probe cannot be deployed in an operating cell
PEMFC fuel cell	J, current density, primary unmeasurable field	3.77 ± 1.03	23.10, IDW	6.1×	Direct energy-device analogue of the nuclear use case

IEA-Wind tall tower (R3 and Editor)	U at 80 m, in-situ anemometry	4.71 ± 0.15	27.86, nearest	+83%	Hub height absent from the input sensor network
IEA-Wind tall tower	U at 100 m, power-law collapse regime	14.70 ± 0.59	95.92, power law	6.5×	Fixed-form laws fail under stratification
GLORYS12 North Atlantic (R3 and Editor)	{T, S, SSH}, multi-modal	4.22 ± 0.06	15.16, kNN	+72%	Disjoint sparse sensor networks; SSH inferred from zero direct sensors
GLORYS12 North Atlantic	SSH channel, zero direct sensors in input	9.11 ± 0.17	37.7, climatology	4.1×	Cross-modal recovery; classical interpolation cannot operate
**Range across three domains**				**+72% to +84%**	

4. *R3 comment: Moreover, the paper presentation is exaggerated. The introduction is unnecessarily long, with repetitive arguments or in-depth discussion of the literature that is not directly related to the main contribution of the paper. The methodological details on the simulations are also distracting from the main results in the paper and could be moved to the methods or supplement.*

Authors' Response: Thanks for this comment. The revised manuscript implements the following structural changes:

The Introduction has been reduced by approximately 30% by: (a) removing repetitive motivational statements that appeared in multiple paragraphs; (b) consolidating the literature review to methods directly relevant to operator-based virtual sensing; (c) removing in-depth discussion of the AI energy crisis framing (Paragraphs 1) which has been condensed to two sentences; (d) concise contribution paragraph added at the end of the Introduction.

Detailed solver settings, convergence criteria, material-property handling, and extended parameter-generation details have been moved to the Methods and Supplementary Methods. The Results section retains only the problem-defining information needed to interpret each experiment, including the input variables, output fields, dataset split, and the specific operator configuration used for the reported comparisons.

The Introduction has been restructured around four functions: (1) the inaccessible-field observability problem; (2) why existing virtual-sensing, observer, and regression methods address different inference regimes; (3) the operator-learning formulation; and (4) the two-layer validation strategy combining nuclear CFD feasibility with real-world validation.

Revision: Introduction reduced by approximately 30%; detailed solver settings, convergence criteria, material-property handling, and extended parameter-generation details moved to the Methods and Supplementary Methods; Results retained only experiment-defining information needed to interpret the comparisons; two-layer validation strategy clarified at the end of the Introduction.

Review of Nature Communications Submission 26-12559 “Virtual Sensing to enable real-time monitoring of inaccessible locations & unmeasurable parameters” by Kazuma Kobayashi, Farid Ahmed, Subhankar Sarkar, Souvik Chakraborty, and Syed Bahauddin Alam

The paper presents an approach to using machine learning to develop state estimators for systems described by partial differential equations (PDEs). This is an active and lively subject within control systems and might or might not lend itself to machine learning. That would depend on many factors including the nature of the PDEs (hyperbolicity, continuity, etc), of the boundary conditions, the complexity of the data/simulation search set, system observability, etc, etc. While mentioning system observers such as the Kalman filter and Luenberger estimators, they do not appear to appreciate what these systems do. They are dynamic system which take the (multiple) measurements and yield estimates of the full systems state, perhaps at different times. The system state is not pointwise the same as the measurements. Line 73 states;

> *Existing virtual-sensing models are intrinsically localized: they estimate only where sensors are present, failing precisely where measurements are absent or infeasible.*

This indicates a fundamental misunderstanding and misrepresentation of state estimation. The state is normally a finite-dimensional vector and the system models have time as a free variable, hence the central role of time in delivering predictions or smoothed results. If one were to remove time from picture and consider vector functions of time over a fixed interval, then one could equally estimate the coordinates of these functions in any given basis set and use a fixed interval smoother to do this. Line 84 says;

> *Consequently, no existing virtual-sensing method satisfies the central requirement of real-world diagnostics: reconstructing complete, physically coherent fields from sparse, heterogeneous measurements.*

The approach proposed in the paper seeks to simulate the PDE system and to use machine learning to fit finitely parametrized models, which are presumed to be universally applicable for changing operating conditions. Line 99;

> *Moreover, neural operators trained across a range of possible operational conditions relax the requirement for continuous retraining when new operating regimes are encountered.*

There is no analysis of how the number of experiments might relate to the accuracy of the fits and the features of the underlying problem.

There is no requirement of stability, be it the dynamics of the system or the numerics of the simulator, stated.

Line 120 goes on;

> *The shift from “solve once per boundary condition” to “learn the operator itself” is therefore the decisive scientific break that makes real-time inference in inaccessible regions physically plausible.*

This is never demonstrated other than by simple smooth examples chosen by the authors. Such uncritical statements abound in the paper. Further, apart from liberal use of words like “real-time,” the method uses

functional expansions of variables as functions of time on a fixed finite interval. Time does not enter the picture, since the whole solution is provided as an operator on the whole input signal, not a prediction using data up to a particular time.

There is a lot of similar word salad such as, Line 234;

> *Because $\hat{\mathcal{G}}_\theta(u)$ is defined for any query set, evaluation on inaccessible coordinates is obtained by replacing Π_Y with arbitrary point sets.*

Line 243;

> *This multiplicative fusion preserves permutation invariance across modalities and yields a compact, modality-agnostic summary of the inputs. Physically, the product structure serves as a low-rank surrogate for multiplicative couplings in the governing PDEs (e.g., convective transport), encouraging a shared latent ψ that supports joint decoding of all output channels.*

The absence of any defined glossary just makes assertive.

Summary

I cannot recommend this paper for publication because its contribution is obscure at best and undemonstratedat worst. It makes inadequate and imprecise contact with state estimation methods for PDEs. The conclusions are asserted but not supported by mathematical argument. I provide below some recent references on PDE observers, which get the estimation problem right without looking for inserting machine learning too early. Note that these are state estimators with a sense of time, e.g. predictors or smoothers. To yield function estimators, you need to propose a basis and estimate coordinates.

H. Yu, Q. Gan, A. M. Bayen, and M. Krstic, “PDE traffic observer validated on freeway data,” IEEE Trans. Control Syst. Technol., vol. 29, no. 3, pp. 1048-1060, May 2021.

J. Wu, J. Zhan and L. Zhang, “Adaptive Boundary Observers for Hyperbolic PDEs With Application to Traffic Flow Estimation,” IEEE Transactions on Automatic Control, vol. 69, no. 1, pp. 651-658, Jan. 2024.

F. Ferrante, A. Cristofaro, and C. Prieur, “Boundary observer design for cascaded ODE-hyperbolic PDE systems: A matrix inequalities approach,” Automatica, vol. 119, 2020, Art. no. 109027.
